# Novel insights into systemic sclerosis using a sensitive computational method to analyze whole-genome bisulfite sequencing data

**DOI:** 10.1186/s13148-023-01513-w

**Published:** 2023-06-03

**Authors:** Jeffrey C. Y. Yu, Yixiao Zeng, Kaiqiong Zhao, Tianyuan Lu, Kathleen Oros Klein, Inés Colmegna, Maximilien Lora, Sahir R. Bhatnagar, Andrew Leask, Celia M. T. Greenwood, Marie Hudson

**Affiliations:** 1 McGill University, 845 Sherbrooke St W, Montreal, H3A 0G4 Canada; 2 Lady Davis Institute for Medical Research, Jewish General Hospital, 3755 Côte Sainte Catherine, Montreal, H3T 1E2 Canada; 3 Research Institute of the McGill University Health Center, Montreal, Canada; 4 University of Saskatchewan, Saskatoon, Canada

**Keywords:** Scleroderma, Systemic sclerosis, DNA methylation, Whole genome bisulfite sequencing, Differentially methylated regions, Smoothing

## Abstract

**Background:**

Abnormal DNA methylation is thought to contribute to the onset and progression of systemic sclerosis. Currently, the most comprehensive assay for profiling DNA methylation is whole-genome bisulfite sequencing (WGBS), but its precision depends on read depth and it may be subject to sequencing errors. *SOMNiBUS*, a method for *regional* analysis, attempts to overcome some of these limitations. Using *SOMNiBUS,* we re-analyzed WGBS data previously analyzed using *bumphunter*, an approach that initially fits *single* CpG associations, to contrast DNA methylation estimates by both methods.

**Methods:**

Purified CD4+ T lymphocytes of 9 SSc and 4 control females were sequenced using WGBS. We separated the resulting sequencing data into regions with dense CpG data, and differentially methylated regions (DMRs) were inferred with the *SOMNiBUS* region-level test, adjusted for age. Pathway enrichment analysis was performed with ingenuity pathway analysis (IPA). We compared the results obtained by *SOMNiBUS* and *bumphunter*.

**Results:**

Of 8268 CpG regions of ≥ 60 CpGs eligible for analysis with *SOMNiBUS*, we identified 131 DMRs and 125 differentially methylated genes (DMGs; *p*-values less than Bonferroni-corrected threshold of 6.05–06 controlling family-wise error rate at 0.05; 1.6% of the regions). In comparison, *bumphunter* identified 821,929 CpG regions, 599 DMRs (of which none had ≥ 60 CpGs) and 340 DMGs (*q*-value of 0.05; 0.04% of all regions). The top ranked gene identified by *SOMNiBUS* was *FLT4*, a lymphangiogenic orchestrator, and the top ranked gene on chromosome X was *CHST7*, known to catalyze the sulfation of glycosaminoglycans in the extracellular matrix. The top networks identified by *IPA* included connective tissue disorders.

**Conclusions:**

*SOMNiBUS* is a complementary method of analyzing WGBS data that enhances biological insights into SSc and provides novel avenues of investigation into its pathogenesis.

**Supplementary Information:**

The online version contains supplementary material available at 10.1186/s13148-023-01513-w.

## Background

Systemic sclerosis (SSc) is a rare autoimmune connective tissue disorder characterized by immune dysregulation, vasculopathy and fibrosis. It is associated with the highest mortality among rheumatic diseases [2, 39]. As with other autoimmune disorders, SSc disease pathogenesis and progression are poorly understood due to the complex contributions of genetic and environmental factors. Consequently, identification of effective therapeutic targets for SSc is limited by both understanding of altered cell and tissue functions, as well as how these depend on interactions with the genome and epigenome. Genetic and epigenetic studies of SSc can provide important insights into disease pathogenesis.

Epigenetic modifications including DNA methylation play a pivotal role in gene expression and are thus plausible factors in the onset and progression of SSc. Most DNA methylation studies to date have been restricted to a limited subset of cytosines in the genome, such as those on the Illumina EPIC array [35]. These approaches provide incomplete information about methylation profiles of the genome [12]. Whole-genome bisulfite sequencing (WGBS) is a more comprehensive assay for profiling DNA methylation, providing data at the single nucleotide level [19]. However, precision of WGBS depends on read depth and it may be subject to sequencing errors. *SOMNiBUS*, a method that we developed for *regional* analysis of DNA methylation across the genome [44], attempts to overcome some of these limitations. By combining information across nearby cytosines with a smooth spline model built onto a quasi-binomial distribution of methylated counts, *SOMNiBUS* uses all available reads and ignores those that are missing. Furthermore, additional parameters allow for potential sequencing errors and adjustments of confounding variables. This platform has the potential to identify previously unobserved regions of differential methylation and lead to greater understanding of biological pathways of disease.

Here, we compare methylation patterns between CD4+ T-cells from 9 women with SSc and 4 healthy female controls previously analyzed [27] using *bumphunter* [1, 17], a two-stage analysis approach that fits *single* CpG associations first, followed by smoothing the association coefficient estimates for nearby cytosines. In contrast, our analysis performed using *SOMNiBUS* [44] consists of a single-stage *regional* analysis method that infers smooth covariate effects across regions while accounting for variable read depth, sequencing errors, missing data patterns and confounders such as age. Our analysis uncovers new loci of differential methylation associated with SSc.

## Results

### Differential methylation of CpG regions using *SOMNiBUS*

**Fig. 1:**
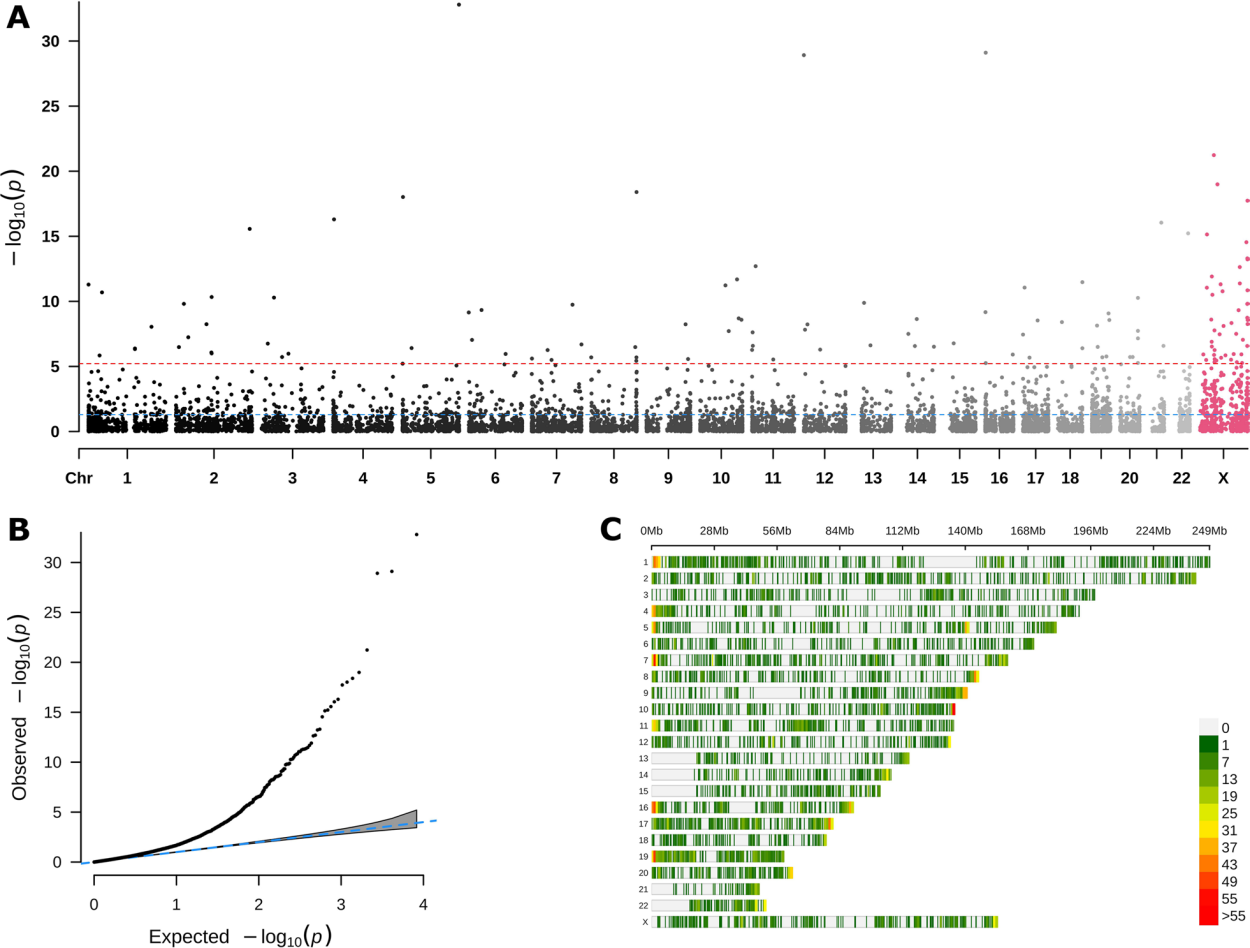
*SOMNiBUS* region-level results. **A** Manhattan plot *SOMNiBUS* *p*-values in CpG regions with ≥ 60 CpGs; the *x*-axis indicates the starting positions of CpG regions; *p*-values are plotted on the − log10 scale. Blue and red dashed lines indicate the significance thresholds used in our study (red: Bonferroni corrected threshold of 6.05e−06; blue: 0.05). **B** Quantile–quantile (QQ) plot of *SOMNiBUS* region-wide *p*-values for 8268 CpG regions containing ≥ 60 CpG sites. The confidence interval under the null hypothesis is shown as a gray shaded area. **C** CpG regions within 1 Mbp density plot, stratified by chromosome

After WGBS, data processing and filtering, we were able to estimate methylation levels for two or more individuals at 6,849,298 CpG dinucleotides. The median read depths of CpGs retained for analysis ranged from 34 to 39 across the autosomal chromosomes, and 35 for chromosome X. No data were available for chromosome Y since this study was comprised of only female subjects. After partitioning the CpG sites into disjoint regions based on CpG site spacing, we obtained 8268 CpG regions of 60 CpGs or more eligible for analysis with *SOMNiBUS*. *SOMNiBUS* identified 131 of these CpG regions as differentially methylated between SSc patients and controls at a significance level below the Bonferroni-corrected *p*-value threshold (Fig. 1A), adjusting for the number of regions analyzed. Quantile–quantile plots of CpG region *p*-values are shown in Fig. 1B, clearly demonstrating that many regions show differential methylation between SSc and controls.

### Genes and networks impacted by differential methylation

By overlaying our results against gene positions from the UCSC genome browser (see Methods), we identified 125 genes associated with our 131 DMRs, which we refer to as differentially methylated genes (DMGs; Table 1 and Additional file 1). Figure 1C displays the locations of the analyzed regions by chromosome. Then, we examined differentially methylated CpG regions (DMRs) at the single-nucleotide level. The estimated smoothed regional coefficients, also known as *β*(*t*), for the association between SSc and methylation (provided on the logit scale) are shown in Additional file 2 for each of the DMGs; these plots also show the corresponding gene region annotations [18]. There is one image per DMR/DMG pair. There were also 24 DMRs which did not overlap with any gene region annotations; no figures are shown in Additional file 2 for these 24 DMRs. Some DMRs may be annotated to more than one gene in the case of a sense/antisense gene-pair, and likewise, one DMG may be associated with multiple DMRs. For our 125 DMGs, 116 DMGs were mapped to a single DMR, 7 DMGs mapped to 2 DMRs, and the sense/antisense gene-pair *GNAS* and *GNAS-AS1* (a lncRNA) mapped to 3 and 4 DMRs, respectively.

**Table 1:** DMGs identified by *SOMNiBUS*

Range	Genes	Count
Chromosome	(Ordered by significance)	
1–22 (Autosomal)	*FLT4, TMEM204, IFT140, WDR97, SHARPIN, SDHAP3, SH3BP2, VPS26C, ARL4C, GALNT18, GFRA1, PARD6G-AS1, KLHL17, NOC2L, ZMIZ1, ZNF232, USP6, MPL, PAX8-AS1, PAX8, MOBP, GNAS-AS1, GNAS, URAD, MEST, MESTIT1, MIR335, ADCY10P1, NFYA, PRR25, DUSP22, ZNF808, FGFR2, ABHD12B, EPS8L1, HOXB3, ANKRD23, LINC01252, KLF2, NAV1, PARP11, LINC00865, H19, DHRS4L1, DHRS4L2, TMEM14C, OTUD7A, RB1, KCNQ1OT1, KCNQ1, TMEM121, SSBP4, IAH1, TRAPPC9, LSP1P3, PARD6G, FIGNL2, GRB10, RGPD8, PSD4, CADM2, DCBLD1, GOPC, SLC7A5, TFAP2E, PSMB2, SIX5, MAGI1, ACTL10, NECAB3, ANKRD27, RGS9BP, ERICH1, FAM83H, ADAP1, UNC93B1, GLI4, ZFP41*	78
X	*CHST7, ZXDA, EMD, FLNA, PDK3, HMGB3, TMEM187, HCFC1, PLXNA3, BCOR, RAB33A, AIFM1, EFNB1, EIF2S3, NEXMIF, CASK, IKBKG, G6PD, DUSP9, DCAF12L2, SLC6A8, PNCK, RPGR, BRCC3, CMC4, MTCP1, BEX2, DKC1, SNORA36A, PGK1, PORCN, DOCK11, MSN, CCNQ, OTUD5, AMMECR1, SMIM10L2B, PRPS2, CDK16, RPS6KA6, DLG3, MAGIX, ZMAT1, MBTPS2, FGD1, CCDC120, C1GALT1C1*	47

### Comparison to *bumphunter*

For the analysis using *bumphunter* in [27], the genome was partitioned into 821,929 CpG regions. *Bumphunter* first identified regions where the regional methylation difference was 0.2 or greater; this quantity is defined by the average of the individual CpG |*β* values| across all CpG sites in a CpG region. *Bumphunter* then retained CpG sites with a false discovery rate-adjusted *q*-value ≤ 0.05; the analysis pipeline retained 8425 CpG regions (7831 CpG regions on autosomal chromosomes and 437 CpG regions on chromosome X). The retained regions had a mean of 1.1 CpG sites per region. Among these 8425 regions, 599 were found to be differentially methylated (584 DMRs on autosomal chromosomes and 15 DMRs on chromosome X), containing an average of 2.5 CpG sites per DMR; with DMRs being defined as a CpG region with *q*-value below 0.05.

It is not straightforward to compare the results obtained here with *SOMNiBUS* to those we obtained with *bumphunter* [27] since the regions are constructed differently, and the statistical significance is not estimated in the same way. As stated above, *bumphunter* uses a genome-wide false discovery rate for significance, combined with a minimum regional methylation difference (analysis details are described in Methods). In contrast, *SOMNiBUS* estimates *p*-values for each analyzed region. We then applied a threshold (6.05e−06) to control the family-wise error rate (FWER) at 0.05 by using a Bonferroni correction for the number of regions analyzed. Nevertheless, with these two very different definitions of significance, *SOMNiBUS* identified a comparable number of DMRs and DMGs when compared to *bumphunter* (Table 2), while starting from 8268 CpG regions—all with ≥ 60 CpG sites per region by definition—with a mean of 108.3 CpG sites per region. Of these 8268 regions, there were 131 DMRs with a mean of 113.2 CpG sites per DMR. In some additional analyses, we also examined the number of DMGs identified by *SOMNiBUS* when a liberal significance of *p*-value < 0.05 was applied; *SOMNiBUS* then identified 1228 DMRs which annotated to 1183 DMGs.

**Table 2:** Comparison of numbers of regions identified by the two methods used for DMR and DMG detection

Method used	# of CpG Regions	# of CpG regions with ≥ 60 CpGs	# of DMRs	# of DMRs with ≥ 60 CpGs	# of DMGs	Mean # of CpG sites per CpG Region	Mean # of CpG sites per DMR
*SOMNiBUS* (*p* < 6.05e−06; FWER < 0.05)	8268	8268	131	131	125	108.3	124.5
*SOMNiBUS* (*p* < 0.05)	8268	8268	1228	1228	1183	108.3	113.2
*Bumphunter* (*q*-value < 0.05)	821,929	0	599	0	340	1.1	2.5

For *SOMNiBUS*, the FWER of 0.05 was estimated by a Bonferroni correction for the number of regions analyzed: 0.05/8268 = 6.05e−06. For *bumphunter*, the software uses permutations to estimate genome-wide false discovery rates, and [27] reported results for *q*-values < 0.05

Hence, to examine the agreement, we compared the set of annotated DMGs identified by both methods. There were 125 DMGs for *SOMNiBUS* (FWER < 0.05; 1.6% of the regions) compared to 340 DMGs for *bumphunter* (*q*-value of 0.05; 0.04% of all regions)*;* no DMRs overlapped between *SOMNiBUS* and *bumphunter* with FWER < 0.05 for *SOMNiBUS*. Therefore, to examine the agreement between *SOMNiBUS* and *bumphunter*, we relaxed the threshold for *SOMNiBUS* to *p* < 0.05, and using this relaxed criterion, we find that 69 genes were identified by both methods. Of these 69 common genes, 30 contained CpG sites in common that were identified as differentially methylated by both methods—overlap of at least 1 CpG site; in this situation, the smoothed *β* values for the 30 *SOMNiBUS* DMRs that overlapped with *bumphunter* DMRs are displayed graphically in Additional file 3. Evidently, no regions with less than 60 CpG sites will be identified by *SOMNiBUS* due to our definition of a CpG region; *SOMNiBUS* requires fairly large regions to estimate all parameters and achieve convergence.

**Fig. 2:**
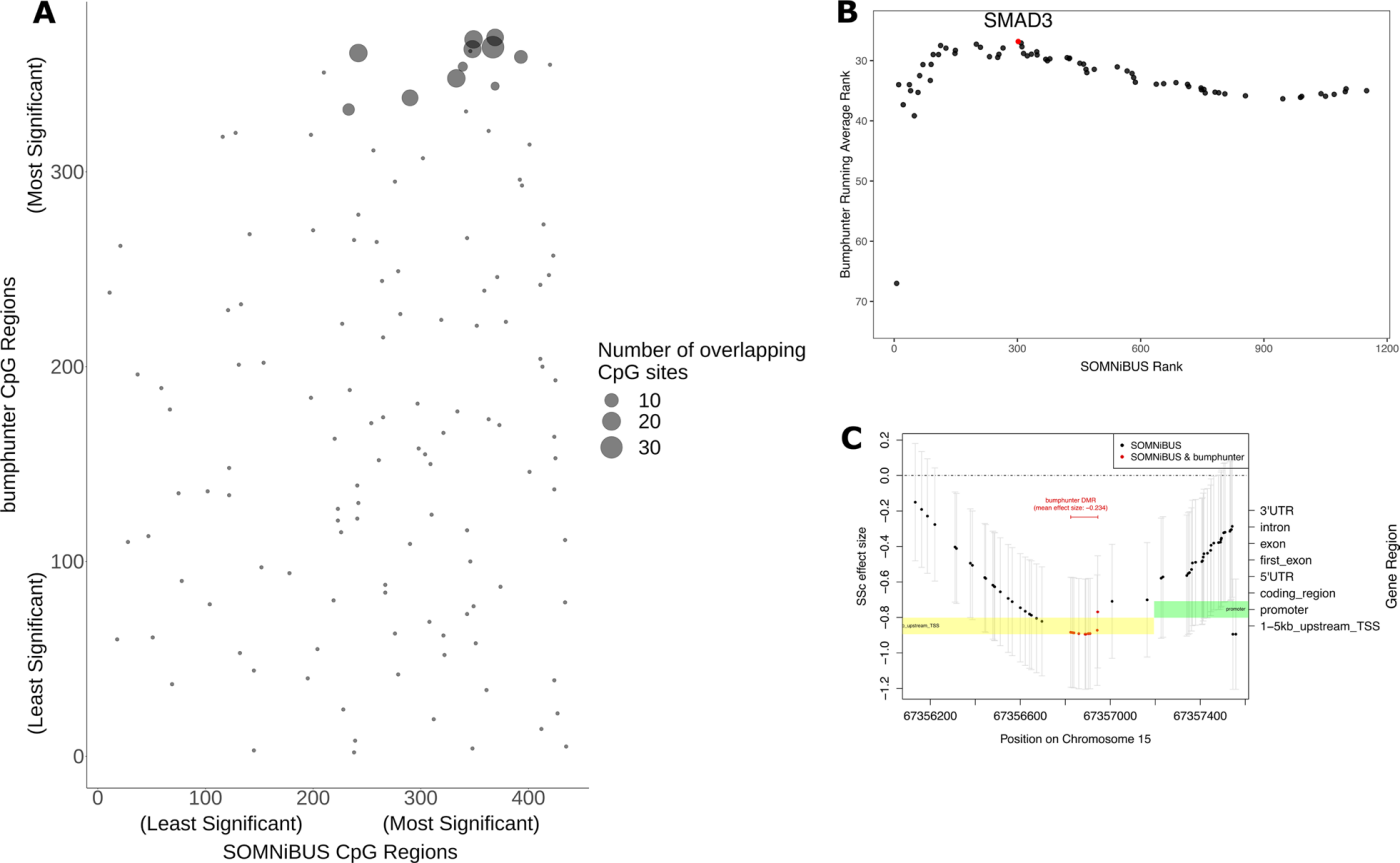
Comparisons of *bumphunter* and *SOMNiBUS* results. **A** Rankings by significance for *SOMNiBUS* and *bumphunter* are shown for 127 CpG regions on chromosome X containing at least one overlapping CpG. The size of a point represents the number of overlapping CpG sites. **B** Average rank score curve of overlapping DMGs on any chromosome identified by both methods. The horizontal axis is the minimum *p*-value for regions assigned to each of 1183 genes with *p* < 0.05 by *SOMNiBUS*. For the 69 genes that were also identified by *bumphunter*, the vertical axis shows the cumulative average ranks of the *bumphunter* *p*-values among their 340 DMGs. When a gene does not match, there is no point shown. The highest average rank was achieved when adding *SMAD3,* which is colored in red. **C** Estimated SSc disease effect on CpG methylation (on the logit scale) for the overlapping DMRs identified by both methods at *SMAD3*. Points (left *y*-axis) indicate the estimated smoothed coefficient at each individual CpG with intersected positions in red. Pointwise confidence intervals are shown in light gray. Structural gene annotations (right *y*-axis) are shown in shaded boxes: green (promoters and first exons; both of which are linked to transcriptional silencing), and yellow (1–5 kb upstream of promoters). The width of *bumphunter’s* DMR is displayed with a red horizontal line, with the average methylation difference in that region indicated just above the line

For a complementary way to look at the agreement between the two methods, we selected the subset of 1249 CpG regions that overlapped by at least one CpG site. Then, in this subset of regions, the significance of each region was ranked by *SOMNiBUS* and *bumphunter.* Overlapping pairs of CpG regions ranked by significance were plotted separately for each chromosome (Additional file 4). The overlap of results for the 127 overlapping CpG regions on chromosome X is shown in Fig. 2A, where the size of the points indicates the number of overlapping CpG sites. The number of overlapping CpG sites was higher (larger points) for the subset of CpG regions that were identified as highly significant in both methods (top right quadrant of the plots).

Next, we examined agreement in *p*-value rankings. We focused on the 1228 *SOMNiBUS* regions with a more relaxed significance threshold of *p* < 0.05 (see Table 2) which corresponds to 1183 genes. *Bumphunter*’s 340 significant DMGs included 69 genes overlapping with the 1183 identified by *SOMNiBUS*. In Fig. 2B, we show the cumulative average rank of these overlapping *bumphunter* results. High proportions of overlap were observed among the genes with ranks between 150 and 300 for *SOMNiBUS*, reaching a maximum when we added *SMAD3* to the cumulative average ranks. Previously in [27], we identified *SMAD3* as a DMG in our *bumphunter* analysis; 1–5 kb upstream of the transcription start site, the average methylation proportion was lower in SSc by 23.4% compared to controls.

*SMAD3* is a signal transducer involved in TGF-*β* signaling [32, 40, 41], a signaling pathway which drives the progression of SSc, [21, 24]. Here, *SMAD3* was identified as a CpG region with a *p*-value of 4.6e−04 which, although small, did not meet our threshold of significance controlling the family-wise error rate (6.05e−06). Nevertheless, both *bumphunter* and *SOMNiBUS* identified hypomethylation 1–5 kbp upstream of the transcription start site in SSc*.* Smoothed *β* values reported by both methods for the overlapping DMRs are shown in Fig. 2C; the larger region analyzed by *SOMNiBUS* provides greater power to detect the differential methylation. The model-derived methylation proportions estimated by *SOMNiBUS,* averaged over the 10 CpG sites identified by both methods, are 0.427 for SSc individuals and 0.639 for controls (lower by 21.2% in SSc; similar to *bumphunter*’s estimate of 23.4%). Differential methylation at *SMAD3* was also detected by Li et al. [24] in CD4+ T lymphocytes, although their hypomethylated CpG is downstream of our region.

Further comparison of DMRs between *bumphunter* and *SOMNiBUS* is difficult due to the differing sizes of their partitioned CpG regions (see Discussion, Methods). Frequency distributions of the number of CpG sites per CpG region, partitioned by method, are shown in Additional file 5.

### Gene annotations for *SOMNiBUS* results

**Fig. 3:**
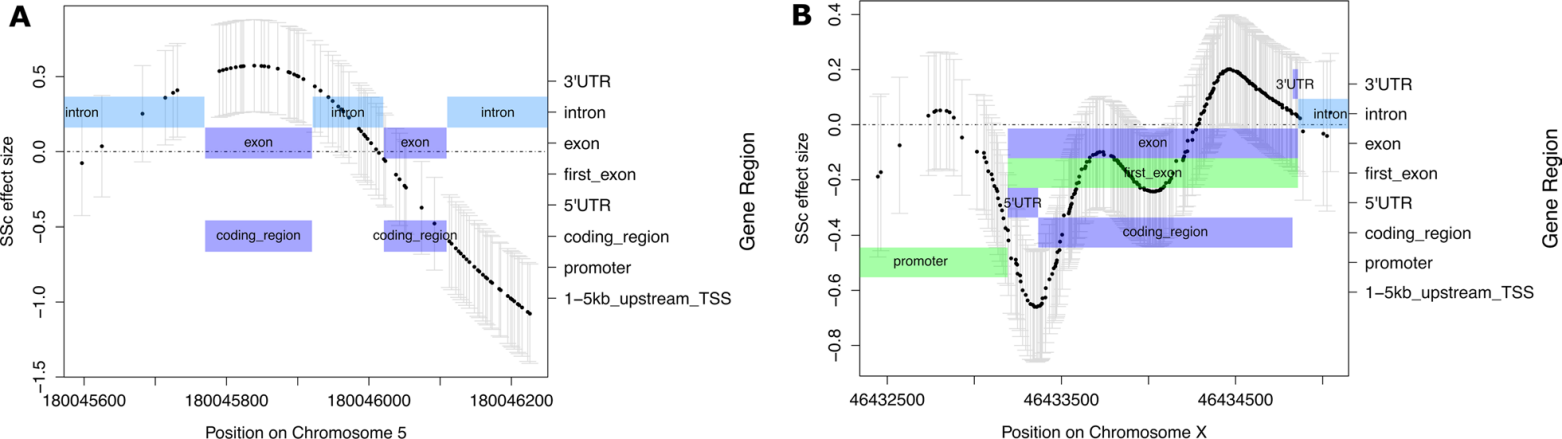
*SOMNiBUS-*estimated smooth association parameters associated with methylation differences between SSc disease and controls. Parameters are shown on the logit scale for DMRs annotated to **A** FLT4, the top ranked autosomal DMG, located on chromosome 5; and **B** CHST7, the top ranked DMG located on chromosome X. Points (left *y*-axis) indicate the estimated smoothed coefficient value (vertical axis) at each individual CpG in the region (*x*-axis), and pointwise confidence intervals are shown in light gray. Structural gene annotations (right *y*-axis) are shown in shaded boxes: light blue (introns), lilac (exons), green (promoters and first exons; both of which are linked to transcriptional silencing)

Of the DMGs we identified with *SOMNiBUS*, the top-ranked gene was *FLT4*. Differential methylation of *FLT4* primarily occurred in the gene body, with hypermethylation of a coding exon (Fig. 3A). *FLT4* encodes for vascular endothelial growth factor receptor 3 (VEGFR-3), which regulates the development and maintenance of the lymphatic system. Abnormal levels of VEGFR-3 and lymphangiogenesis have been reported in SSc [6, 7, 15, 29], and we previously reported its phenotypic presentation on the fingers of an SSc patient [14]. Two differentially methylated CpG sites annotated to *FLT4,* located downstream of *SOMNiBUS’* DMR, were also detected in an epigenetic study of SSc CD4+ T lymphocytes [24].

The top ranked gene on chromosome X—the chromosome with the most DMRs—was *CHST7*, which catalyzes the sulfation of glycosaminoglycans (GAGs) in the extracellular matrix (ECM). *CHST7* was previously characterized as being differentially expressed at a rate of 3.56-fold in SSc dermal fibroblasts of 15 twin pairs discordant for SSc compared to dermal fibroblasts of 5 controls in a study to discriminate between genetic and nongenetic molecular pathways implicated in SSc pathogenesis [45]. Here, hypomethylation was observed on the first exon of *CHST7* (Fig. 3B). Since methylation in this region is hypothesized to block transcription initiation, our hypomethylation results suggest *CHST7* is being transcribed at higher rates in SSc patients. Thus, the hypo-methylation we observe in the first exon region of *CHST7* could potentially highlight an epigenetic response to SSc-associated fibrosis.

We also annotated DMRs using the hg19 database to long-non-coding functional RNAs (lncRNA), which play an important role in the regulation of many epigenetic processes. LncRNAs are a broad class of RNA genes that act as gene regulators through their involvement in various epigenetic processes such as DNA methylation [31]. RNA sequencing studies have previously revealed differentially expressed lncRNAs in the skin tissue of SSc patients [33, 42]. However, the role lncRNAs in SSc—and in general—is still poorly understood. Of our 125 observed DMGs, the sense-antisense gene pair *GNAS* and *GNAS-AS1* (the corresponding antisense lncRNA) shared 4 distinct DMRs, more than any other DMG identified. Moreover, mutations in the *GNAS* gene have been previously associated with calcinosis cutis, a debilitating feature that can be present in SSc [37].

For functional analysis and detection of networks, we analyzed the 125 identified DMGs with ingenuity pathway analysis (*IPA*)*.* From these genes, *IPA* identified possibly implicated gene networks, with cancer and endocrine system disorders identified as the top-ranked network and connective tissue disorders identified as the third-ranked network. The top 5 networks are shown in Table 3; all enriched networks can be seen in Additional file 6. Enriched networks from analyses only using DMGs on autosomal chromosomes are shown in Additional file 7, and only on chromosome X are shown in Additional file 8. Results of our network analysis were compared with a 2019 WGBS study conducted on twin pairs discordant for SSc; we found evidence of agreement for the following pathways: cancer, gastrointestinal disease, and organismal injury and abnormalities [36].

**Table 3:** Top 5 networks impacted by SSc-associated CpG differential methylation

Top diseases and functions	Molecules in network (alphabetical)	Score	Focus molecules
Cancer, endocrine system disorders, organismal injury and abnormalities	*Akt, ****ARL4C****, ****BEX2****, ****C1GALT1C1****, ****CASK****, ****CCDC120****, ****CDK16****, CG*, Cyclin A, ***DLG3***, estrogen receptor, ***FGFR2****, ****FLT4****, FSH, ****GALNT18****, ****GFRA1****, ****GRB10***, Growth hormone, ***H19****, ****HMGB3****, Lh, ****MAGI1****, ****MEST****, ****mir-335****, ****MPL****, ****NAV1****, ****NFYA****, ****PARD6G****, ****PAX8****, ****PORCN***, Proinsulin, *Rb*, ***TMEM204***, *YAP/TAZ, ****ZNF232***	55	25
Developmental disorder, gastrointestinal disease, organismal injury and abnormalities	***ADAP1****, Alp, BCR* (complex), *Creb, ****DOCK11****, ****DUSP9****, ****EPS8L1****, ERK1/2, ****FGD1****, ****FLNA****, ****G6PD****, GTPase, ****KCNQ1****, ****KCNQ1OT1****, MAP2K1/2,* **MBTPS2**, ***MSN****, ****OTUD5****, ****OTUD7A****, p70 S6k, Pdgf* (complex), *PDGF BB*, phosphatase, *PI3K (family), Pka, PP2A, ****PSMB2****, ****RB1****, ****RPS6KA6****, Rsk, ****SH3BP2****, ****SHARPIN****, Shc, Srebp*, transcription factor	36	18
Connective tissue disorders, hereditary disorder, organismal injury and abnormalities	26 s Proteasome, Actin, caspase, *CD3, ****CHST7****, ****DKC1****, EGLN, ****EIF2S3***, *F Actin, ****FAM83H****, ****GLI4****, ****HCFC1****, HISTONE, Histone h2a,* Histone h3*,* Histone h4*, Hsp70, Hsp90, ****IKBKG****,* Immunoglobulin*, ****KLF2****, ****NECAB3****, NFkB* (complex)*, ****NOC2L****, Notch, ****PARP11****, PI3K* (complex)*, Rnr, TCR, ****TMEM121****, ****TMEM14C****, ****TRAPPC9****, Ubiquitin, ****USP6****, Vegf*	28	15
Cancer, cell death and survival, organismal injury and abnormalities	*ADRB2, BARX2, ****CADM2****, CAMK1G, CIITA, CREB1, CREBBP, DDX39A, FAS, ****GNAS-AS1****, HEIH, ****HOXB3****, IFNG, IL32,* iodine*, ****NEXMIF****, NR4A1, ****PAX8-AS1****, Pde4d, PDHA2, ****PDK3****, ****PRPS2****, ****PSD4****, ****RGS9BP****, SETD7, SMARCA4, ****SMIM10L2B****, ****TFAP2E****, ****TMEM187****, TP53, TRPM1, TULP4, ****ZFP41****, ****ZMIZ1****, ****ZXDA***	28	15
Cardiac dilation, hereditary disorder, organismal injury and abnormalities	***AIFM1****, AMPK, ****BCOR****, ****BRCC3****, Calmodulin,* calpain*, ****CMC4****,* cytokine*, ****DUSP22****, ****EFNB1****, ****EMD****, ERK, ****GNAS****, ****GOPC****, IFN Beta, IgG, Igm,* Insulin, *Interferon alpha, Jnk, Mapk, Mek, Nfat (*family*), P38 MAPK, ****PGK1****, Pkc(s), PTK, Ras* homolog*, Sapk, ****SLC6A8****, ****SLC7A5****, SRC (*family*), ****SSBP4****, STAT5a/b, ****UNC93B1***	26	14

DMGs identified by *SOMNiBUS* are bold

**Fig. 4:**
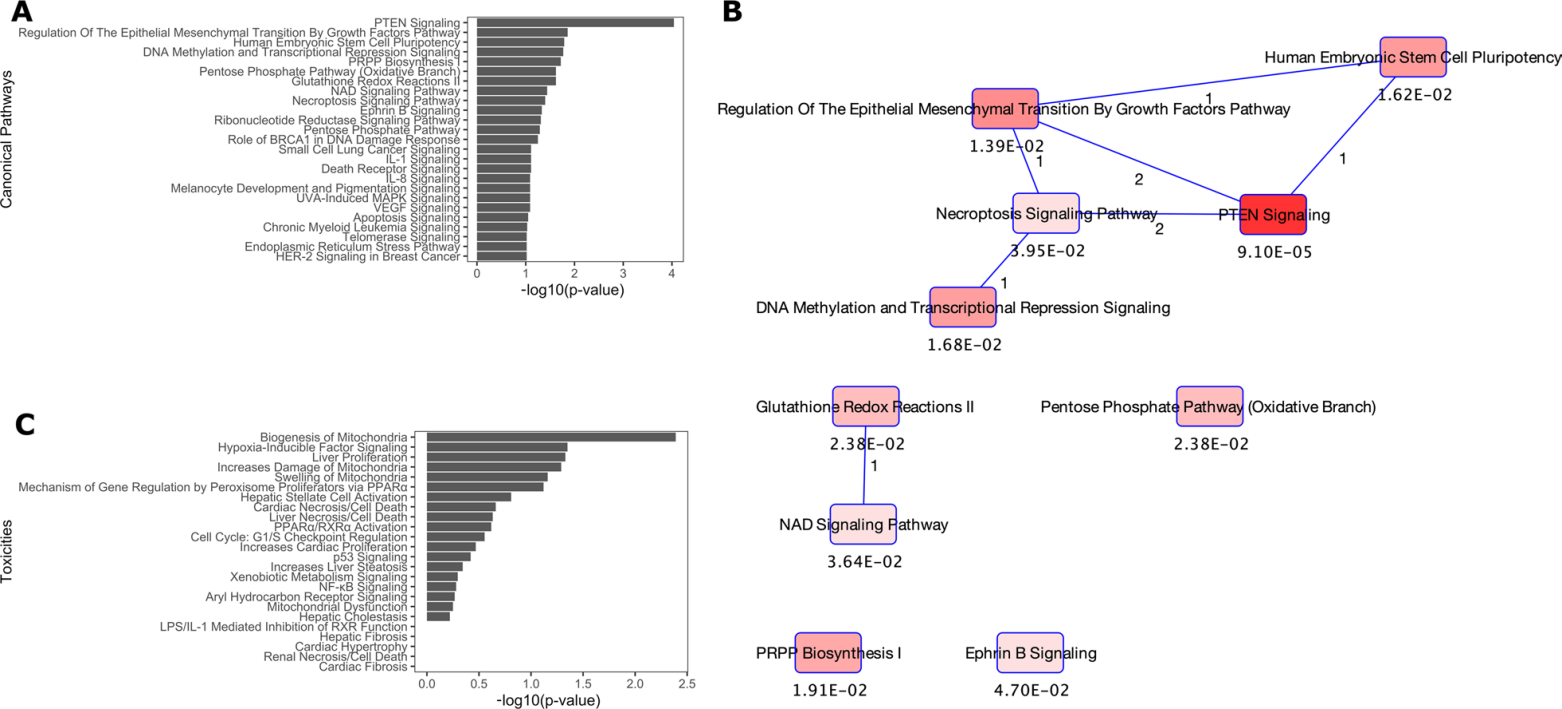
Results of pathway analyses of DMGs identified by *SOMNiBUS*. **A** Top 25 canonical pathways impacted by SSc-associated CpG differential methylation, identified by *IPA*; bars indicate *p*-values on the − log10 scale. **B** Network graph of the top 10 overlapping canonical pathways from panel 3a. **C** Toxicities known to originate from genes identified as SSc-associated DMGs by *SOMNiBUS*; bars indicate *p*-values on the − log10 scale

Our functional analysis with *IPA* was able to detect immune-cell specific pathways in these extracted CD4+ T lymphocytes. Several canonical pathways identified by *IPA* demonstrate strong biological plausibility (Fig. 4A and B), including among others, pathways involved in skin fibrosis, such as PTEN [26, 34] and Ephrin [22, 23, 43], which are particularly interesting pathways to observe in our analyses of immune cells. *IPA* also identified SSc-associated abnormal methylation of genes implicated in the signaling of hypoxia-inducible factors, which lead to hypoxia, a cellular environment with known involvement in the pathogenesis of SSc-associated fibrosis [16], along with other biologically plausible toxicities (Fig. 4C).

## Discussion

### Strengths of *SOMNiBUS*

The way we defined a DMR with *SOMNiBUS* allows our regions to span all parts of the genome containing concentrated and closely positioned CpG sites that were captured by the sequencing library. This includes the detection of differential methylation in CpG islands, common genomic features with a high density of CpG dinucleotides which often occur in promoter regions in mammalian genomes [9]. Previous platforms for measuring methylation such as the Illumina 450 K [3, 30] or EPIC arrays [35] contained a preponderance of CpG islands. *SOMNiBUS* broadens regions beyond islands and includes many other genic or intergenic sections.

Since our analysis was performed using *SOMNiBUS*, a single-stage regional detection method, the number of tests performed corresponded to the number of partitioned CpG regions containing ≥ 60 CpG sites, namely 8268. This enabled us to use a Bonferroni adjusted significance threshold of 6.05e−6 to control family-wise error rate. This is a more lenient threshold than would be required if we tested each CpG separately, and therefore, results in a more sensitive analytic approach. Furthermore, the *SOMNiBUS* algorithm borrows information across nearby CpG sites within a region, which also improves power for detection of differential methylation. For comparison to our earlier work [27], we set a very lenient regional significance threshold of *p* < 0.05 for *SOMNiBUS*, and a permutation-based false discovery rate (FDR) or *q*-value threshold < 0.05—that is, a *p*-value in the 95th percentile of all CpG region *p*-values across 40 genome-wide permutation tests—for our previous analysis using *bumphunter*. The altered *SOMNiBUS* threshold included more regions, thereby making our analysis of overlap more interesting.

### Comparison with *bumphunter*

One of *SOMNiBUS’s* defining features is its ability to detect long-range differential methylation patterns, allowing for nucleotide-level inspection of epigenetic fluctuations across genic regions. This is in contrast to other methods of differential methylation detection, which tend to detect smaller regions, thus, allowing for more regions to be tested but consequently, limiting the amount of information that can be gained from each test. For that reason, we decide to compare *SOMNiBUS* to *bumphunter*, the method that provided the smallest median DMR size of 6 CpG sites per DMR in a comparison between four other supervised DMR detection methods [28]; this comparison consisted of searching for overlapping DMRs and comparing the overlaps in terms of detected effect sizes and peak positions. Together, this study in conjunction with [27]—our two current WGBS studies on SSc—provides a multifaceted view of epigenetic contributions to SSc pathogenesis by focusing on both short-range and long-range differential methylation patterns.

A direct-comparison of the analyses performed using both methods is hindered by fundamental differences in their partitioning of CpG regions, with *SOMNiBUS* requiring pre-specified CpG regions of 60 CpG sites or more, and *bumphunter* automatically partitioning CpG regions of any size based on spacing for analysis and comparing mean estimated disease effect values reported to those obtained through replicate analyses from genome-wide permutations where disease status was randomly reassigned. Our *bumphunter* analysis had also filtered CpG regions by requiring that the mean magnitude of all smoothing coefficients within their bumps be greater than 0.2. We did not apply a similar mean difference filter to our *SOMNiBUS* analyses since *SOMNiBUS* requires larger CpG regions for analysis (Additional file 5) and is thus, representative of long-range methylation patterns which are expected to smoothly fluctuate throughout various genic regions, and potentially consists of both hypo- and hyper-methylated areas. Thus, filtering based on a region-wide average of all CpG site *β* values would not be appropriate.

### Annotation and pathway analysis

We performed pathway analysis on the DMGs linked to our CpG regions with evidence for differential methylation at FWER < 0.05. DMGs identified by *SOMNiBUS* may be associated with multiple DMRs, each of which may contain both hyper- and hypo-methylated subregions (Additional file 2).

Although we contrast the pathways that have been highlighted by the two analyses and we did see overlap, the two analytic strategies are complementary. *Bumphunter* finds small DMRs, whereas *SOMNiBUS* targets much larger DMRs by definition and may capture complex patterns of epigenetic regulation around relevant genes.

### Covariates, sex, and chromosome X

We acknowledge the small sample size of this study and the possible presence of confounding due to demographic differences between cases and controls. Ethnicity and smoking status were not included as covariates due to the small sample size; future validation of findings in patients and controls separately by ancestral origin would be warranted. Hence, our model consisted of SSc disease status as a main effect, with an adjustment for the continuous covariate of age; age adjustments for both *SOMNiBUS* and *bumphunter* were performed within the DMR detection step.

Since we had no male SSc patients with WGBS data, we limited this analysis to only the female participants. On the other hand, the inclusion of women only in our analysis substantially simplified the interpretability of our analysis of chromosome X. In addition to men having only one X chromosome, the expression levels of genes on chromosome X are known to be different in XX cells and XY cells depending on the X-inactivation status. Many immune-related genes are known to reside on chromosome X and its role in the sex-bias of immune-related diseases has been well established [25, 38]. Differing levels of gene expression caused by increased methylation in the paternal X chromosome compared to the maternal X chromosome has been shown to contribute to the varying levels of immune response between men and women [10] and would make interpretation of results more challenging, particularly for studies on immunological diseases such as SSc.

### Runtime

*SOMNiBUS* for 8268 regions in parallel for all chromosomes took a total of 137.3 min to run on a single Xeon E5-2660 v2 processor with ten 3.0 GHz CPU cores.

## Conclusions

Using *SOMNiBUS*, a recently developed computational method, we characterized DNA cytosine methylation patterns across the genome in females with and without SSc. Our method was comprehensive, highlighting pathways and genes known to be of interest in SSc disease pathogenesis, thereby providing biological plausibility for our data, as well as novel pathways and genes providing potential new insights into disease pathogenesis. Our method also generated nucleotide-level information on SSc-associated methylation of genes. The findings of this study can serve as the base for future investigation into genetic and epigenetic targets of interest in SSc pathogenesis.

## Methods

### Study subjects

From an ongoing SSc research cohort based at McGill University, Montreal, Canada, we recruited 9 SSc female patients and 4 female control subjects who provided informed consent. At the time of sampling, none of the 9 SSc patients were on immunosuppressive drugs. Three patients had previously taken methotrexate and mycophenolate, but these medications had been discontinued for over a year prior to enrollment in this study. Disease duration was 10.4 ± 7.0 years for the 9 SSc cases, of which 6 had diffuse and 3 had limited cutaneous skin involvement. Detailed characteristics of study subjects are shown in (Table 4).

**Table 4:** Clinical characteristics of study individuals

	SSc (*N* = 9)		Controls (*N* = 4)	
	Mean or %	SD or N	Mean or %	SD or N
Age, years	52.8	16.2	37.2	19.8
Female, %	100	9	100	4
*Ethnicity, %*				
Caucasian	77.8	7	50	2
Asian	22.2	2	25	1
Other	–	0	25	1
*Smoking, %*				
Current	11.1	1	–	0
Past	22.2	2	25	1
Never	55.6	5	75	3
Unknown	11.1	1	–	0
Disease duration, years	10.4	7.0		
Interstitial lung disease, %	11.1	1		
Arthritis, %	11.1	1		
Myositis, %	22.2	2		
Raynaud’s, %	100	9		
*Anti-nuclear antibodies*				
Titer ≥ 1:80, %	100	9		
Titer ≥ 1:160, %	66.7	6		
Titer ≥ 1:640, %	55.6	5		
*Blood biochemical indices*				
C-reactive protein (CRP), mg/L	29.5	65.3^¶^		
Erythrocyte sedimentation rate, mm/hr	23.7	14.1		
Abs. whole blood cell (WBC), K/μL	8.2	4.6		
Abs. lymphocytes, K/μL	1.6	0.7		
Abs. monocytes, K/μL	0.7	0.7^¶^		
*Disease-specific variables*				
Limited skin disease, %	33.3	3		
Diffuse skin disease, %	66.7	6		
Immunosuppressive medication^§^, %	33.3	3		

^¶^Over-dispersion due to extreme values

^§^Methotrexate or Mycophenolate Mofetil in the past

### Purification and sequencing of cells

As described in [27], each study subject had 40 ml of blood drawn and processed within 4 h. CD4+ T cells [anti-CD4 microbeads (Miltenyi Biotec and auto-MACS] were positively selected, and samples with purity > 95% purity were retained for genomic DNA extraction and sequencing. Samples were subsequently processed with in-house DNA isolation and Illumina HiSeq 4000 PE 100 WGBS workflows at McGill University and the Genome Quebec Innovation Centre. Quality control steps assessed quantity, quality, and purity of genetic material using fluorescence assay quantification, agarose gel electrophoresis and NanoDrop nucleic acid quantification.

### Data processing and filtering

WGBS data were aligned to the human genome GRCh37 (hg19) with *annotatr version 1.22.0*. Coverage by both strands in the paired-end sequencing library was required for each of the cytosine nucleotides retained after alignment. Estimated methylation levels were determined by merging methylated and unmethylated counts from both the forward and reverse cytosines, while requiring at least a read depth of 3 for each, and a difference in the estimated methylation proportions of less than 0.2 between directions.

### Algorithms

The motivation surrounding the construction of both DMR detection algorithms is the assumption that methylation patterns across the genome are smoothly varying in nature, and therefore, that estimates of differential methylation for a given CpG site can benefit from borrowing information provided by neighboring CpG sites.

Previous work investigating short-range methylation correlations showed significant correlation of DNA methylation levels for CpG sites spaced less than 1000 bp apart [8]. Within the context of differential methylation, this amounts to modeling smooth regional coefficients for the SSc effect on methylation (on the logit scale, termed “*β* values”; not to be confused with the commonly used methylation level measure, "Beta values”, which ranges from 0 to 1 and was used to filter CpG sites in *Data Processing and Filtering*). We partitioned CpG regions prior to analysis with *SOMNiBUS*, and prior to the smoothing of effect sizes in *bumphunter’s* analysis. *Bumphunter* required CpG sites within a CpG region to be spaced a maximum of 200 bp apart, allowing for continuous smoothing across all positions within a CpG region. For *SOMNiBUS*, regions were also defined gaps of 200 bp or more, with the additional restriction that they had to contain at least 60 CpGs (details below under DMR detection). Smoothing of the effect sizes was performed independently for all CpG regions partitioned in both analyses. Both methods provide estimates for the smoothed regional coefficients for the SSc effect on methylation (logit scale) for all CpG sites retained after data filtering.

### *Bumphunter*

*Bumphunter* [17] is a two-stage differential methylation detection algorithm which first estimates single CpG site slope estimates using ordinary linear regression models, then smooths the site-specific slope estimates (for a covariate of interest such as the comparison of SSc versus controls) across all CpG sites throughout the genome. The use of a linear model in bumphunter’s algorithm allows for adaptability to different study designs and covariates; however, it would require modifications to appropriately treat count data such as those produced through next-generation sequencing [11], such as the sequencing technology used in our study, Illumina HiSeq, one of the most widely used.

### *SOMNiBUS*

*SOMNiBUS* [44] is a single-stage differential methylation detection algorithm that utilizes the flexibility and interpretability offered by a generalized additive model (GAM) [13] to estimate the coefficients for covariates on methylation levels as smoothed functions. Spline functions model the relationship between a set of covariates and the methylation proportions within a region, adjusting appropriately for the read counts. This approach allows for simultaneous coefficient estimation using regression fitting, smoothing of coefficients through penalization, and estimation of statistical uncertainty for the complete model. Penalty terms added to the log-likelihood function control the smoothness of the curves. In addition, the *SOMNiBUS* algorithm assumes true methylated counts follow an over-dispersed binomial distribution, respecting the count data produced by next-generation-sequencing (NGS).

*SOMNiBUS’s* model in this study, therefore, estimates the smooth relationship between disease status and methylation levels within each region, while simultaneously adjusting for a smooth relationship with age across the region.

### DMR detection

*SOMNiBUS version 1.0.0* [<https://www.bioconductor.org/packages/release/bioc/html/SOMNiBUS.html>] was used to identify differentially methylated regions (DMRs) for the comparison between female SSc cases (*N* = 9) versus female controls (*N* = 4) adjusted for age in years. Since read depth is quite variable across genomic positions, to ensure sufficient sample size for analyses, we required coverage of at least 3 by both strands in the paired-end sequencing library and retained for analysis only cytosines with coverage for at least 6 of 9 SSc cases and at least 3 of 4 controls and a difference in the estimated methylation proportions of less than 0.2 between directions. This criterion matches what was applied in our previous study [27] using *bumphunter* version 3.3 [<https://www.bioconductor.org/packages/release/bioc/html/bumphunter.html>]. We then clustered the retained CpG sites into non-overlapping CpG regions along the genome, by dividing the genome into disjoint regions when CpG sites were spaced 200 bp or more apart. For regional analysis with *SOMNiBUS*, only regions with at least 60 CpG sites were retained, leaving 8268 regions across the genome for analysis. After analysis with *SOMNiBUS*, a region was called differentially methylated if the *SOMNiBUS* region-wide summary *p*-value was smaller than the Bonferroni corrected *p*-value threshold of 6.05e−06 (0.05/8268 tests). Density plots of the locations of DMR start sites, stratified by chromosome, are shown in (Fig. 3C).

### Annotation and functional analysis

DMRs identified were linked to genes using *annotatr* version 1.16.0 [<https://bioconductor.org/packages/release/bioc/html/annotatr.html>] [5] based on human genome annotations from the UCSC genome browser hg19 database [<http://hgdownload.soe.ucsc.edu/goldenPath/hg19/database/> Accessed 02 October 2022]. DMRs were also linked to long-non-coding RNAs (lncRNAs), based on annotations from the GENCODE lncRNA reference database [<https://www.gencodegenes.org/human/release_38lift37.html> Accessed 02 October 2022]. Since methylation counts were merged from forward and reverse sequencing during data processing, DMRs annotated to antisense lncRNAs, which function as co-regulators in conjunction with their sense genes, were reported alongside their sense counterparts and regarded as distinct DMGs. We annotated DMRs to a gene if the differentially methylated region’s start and end sites overlapped any position of one of 9 gene-related structural annotations: 1–5 kb upstream of the transcription start site, promoter, gene coding region, 5′ untranslated region, exon, first exon, intron, 3' untranslated region, or intergenic region. Our choices for gene regions allow for capture of epigenetic dysregulation in both the gene body and the promoter, as well as a larger 4 kb window upstream of the transcription start site. In addition, we separately annotated first exons, as identified in the hg19 database, since methylation levels in first exons have been hypothesized to affect gene transcription in a manner similar to promoter methylation, resulting in gene silencing [4]. Any gene that was linked, through one of these structural annotations, to at least one DMR was termed a differentially methylated gene (DMG). We did not distinguish between hyper- and hypo-methylated DMRs in annotation; as such, DMGs that are associated with multiple DMRs in different genic subregions may not necessarily show a consistent direction for methylation differences across the gene. For each DMG linked to a significant DMR, the patterns of differential methylation are illustrated by graphing the smoothed coefficient estimates (*β*(*t*) for SSc versus controls) from *SOMNiBUS* against chromosomal position, while overlaying the positions of functional regions on the graphic.

Functional analysis was performed using *Ingenuity Pathway Analysis* [*IPA*, QIAGEN Inc.; <https://www.qiagenbioinformatics.com/products/ingenuitypathway-analysis> ; [20] on the genome-wide set of DMGs and separately for DMGs of autosomal chromosomes and of chromosome X. This functional analysis was performed for DMGs identified by *bumphunter* or by *SOMNiBUS*. DMRs that were not linked to any genes were not included in the functional analysis.

We note that CpG region density plots (Fig. 1C) indicated that many of the CpG regions are contained at the start and ends of chromosomes. Alignment is known to be more challenging in telomeric regions, so any DMRs located near them should be interpreted cautiously.

### Comparison of results between *bumphunter* and *SOMNiBUS*

Comparisons between identified regions and genes are difficult due to the differing sizes of CpG regions analyzed. Therefore, we chose to examine a general overlap definition for all CpG regions retained by both methods after data processing and filtering. CpG regions were considered overlapping if they shared any CpG sites. We plotted effect sizes for all overlapping CpG regions between *SOMNiBUS* and *bumphunter* in Additional file 3. The number of overlapping CpG sites for the overlapping regions, stratified by chromosome, is shown in Additional file 4.

Significance thresholds are also difficult to standardize between the two methods as *bumphunter* reports *q*-values and *SOMNiBUS* reports *p*-values. We chose to use the published significance threshold from Lu et al. [27] which corresponds to a *q*-value of 0.05 for *bumphunter*. For *SOMNiBUS*, our primary analysis is based on a family-wise error rate threshold of 6.05e−06 (Bonferroni-corrected for 8268 CpG regions)*.* Characterizations of overlap used a more lenient threshold of *p* < 0.05, simply to be able to identify more overlapping regions.

## Supplementary Information

**Additional file 1**: Annotated differentially methylated regions detected by *SOMNiBUS*

**Additional file 2**: Nucleotide-level smoothed regional disease effect coefficients for all DMGs identified by *SOMNiBUS*

**Additional file 3**: Nucleotide-level smoothed regional disease effect coefficients for all CpG regions identified by *SOMNiBUS* with *p*-value < 0.05 and identified by *bumphunter* with *q*-value < 0.05

**Additional file 4**: Overlapping CpG regions partitioned by *SOMNiBUS* and *bumphunter* ranked by significance, stratified by chromosome

**Additional file 5**: Histogram of number of CpG sites per CpG region partitioned by *bumphunter* and *SOMNiBUS*

**Additional file 6**: Networks impacted by SSc-associated CpG differential methylation identified by IPA for all 125 DMGs in the genome

**Additional file 7**: Networks impacted by SSc-associated CpG differential methylation identified by IPA for the 78 DMGs located on an autosomal chromosome

**Additional file 8**: Networks impacted by SSc-associated CpG differential methylation identified by IPA for the 47 DMGs located on chromosome X

## Data Availability

All code used to generate the analyses and figures for this article are deposited in https://github.com/jeffreycyyu/ssc_methylation_hudson.
