# Supplementary material for: Novel insights into systemic sclerosis using a sensitive computational method to analyze whole-genome bisulfite sequencing data
Source: Clin Epigenetics. 2023 Jun 3;15:96. doi: 10.1186/s13148-023-01513-w (PMC10239181; doi:10.1186/s13148-023-01513-w)

## Slide 1
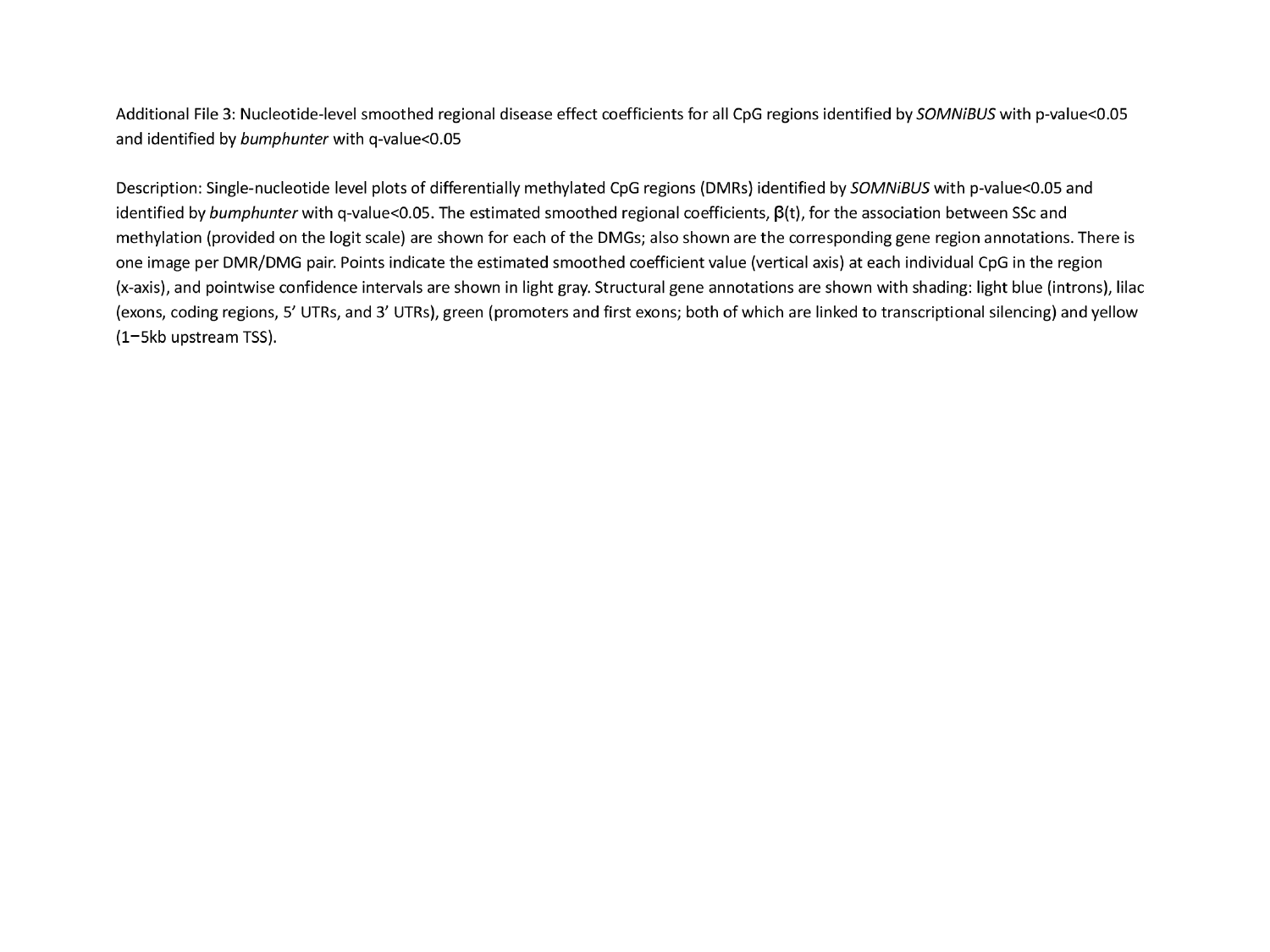

## Slide 2
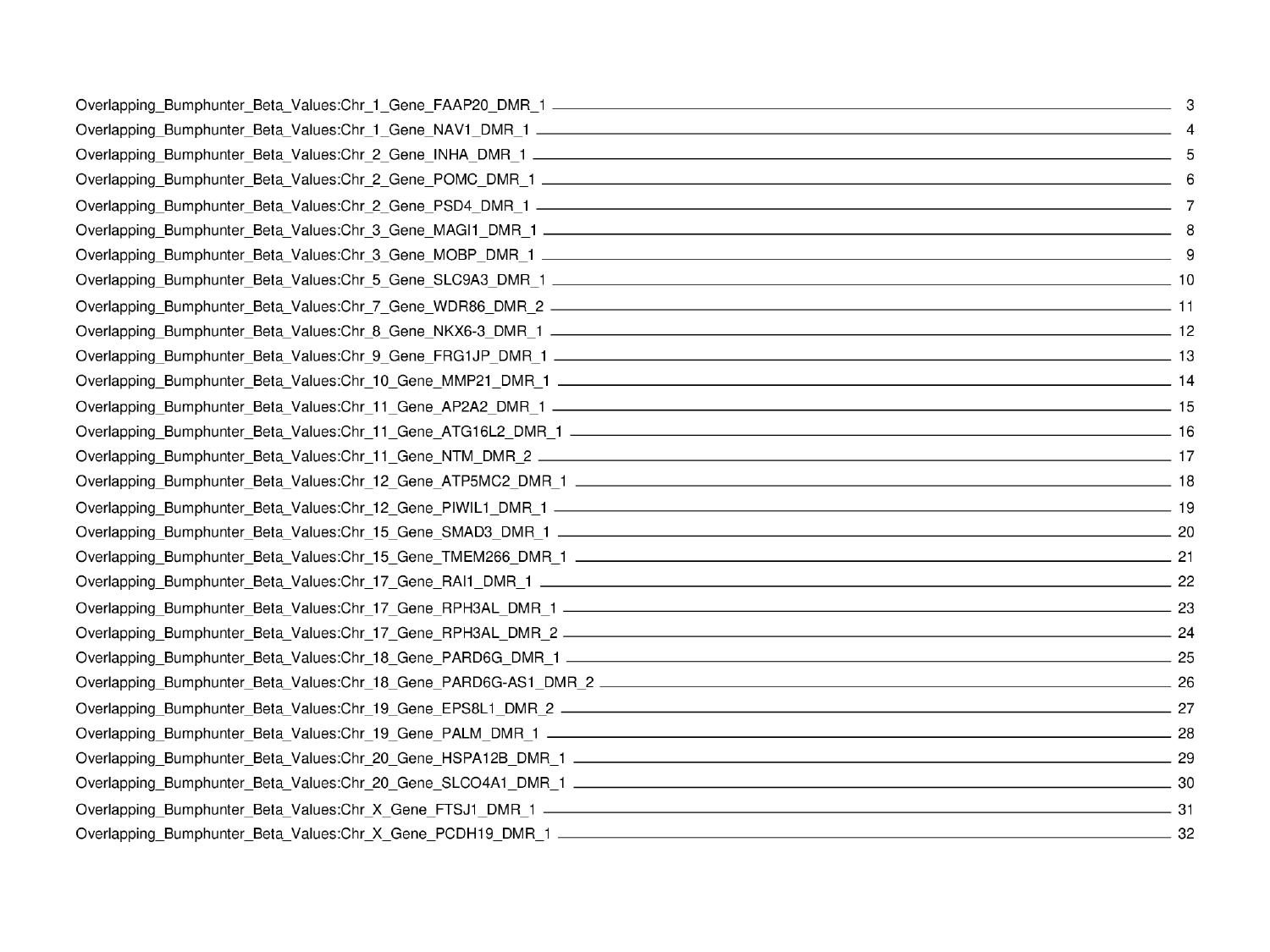

## Slide 3
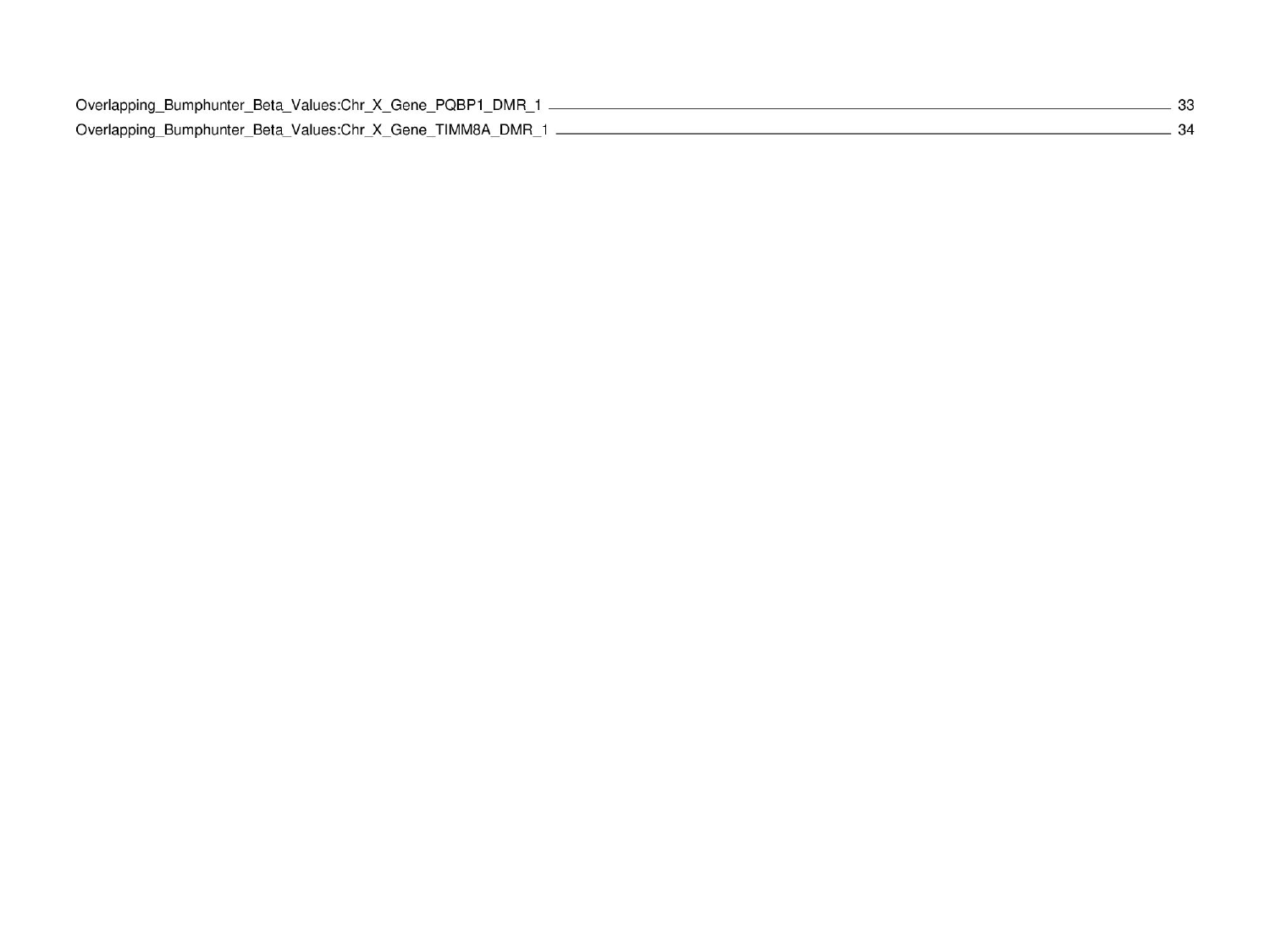

## Slide 4
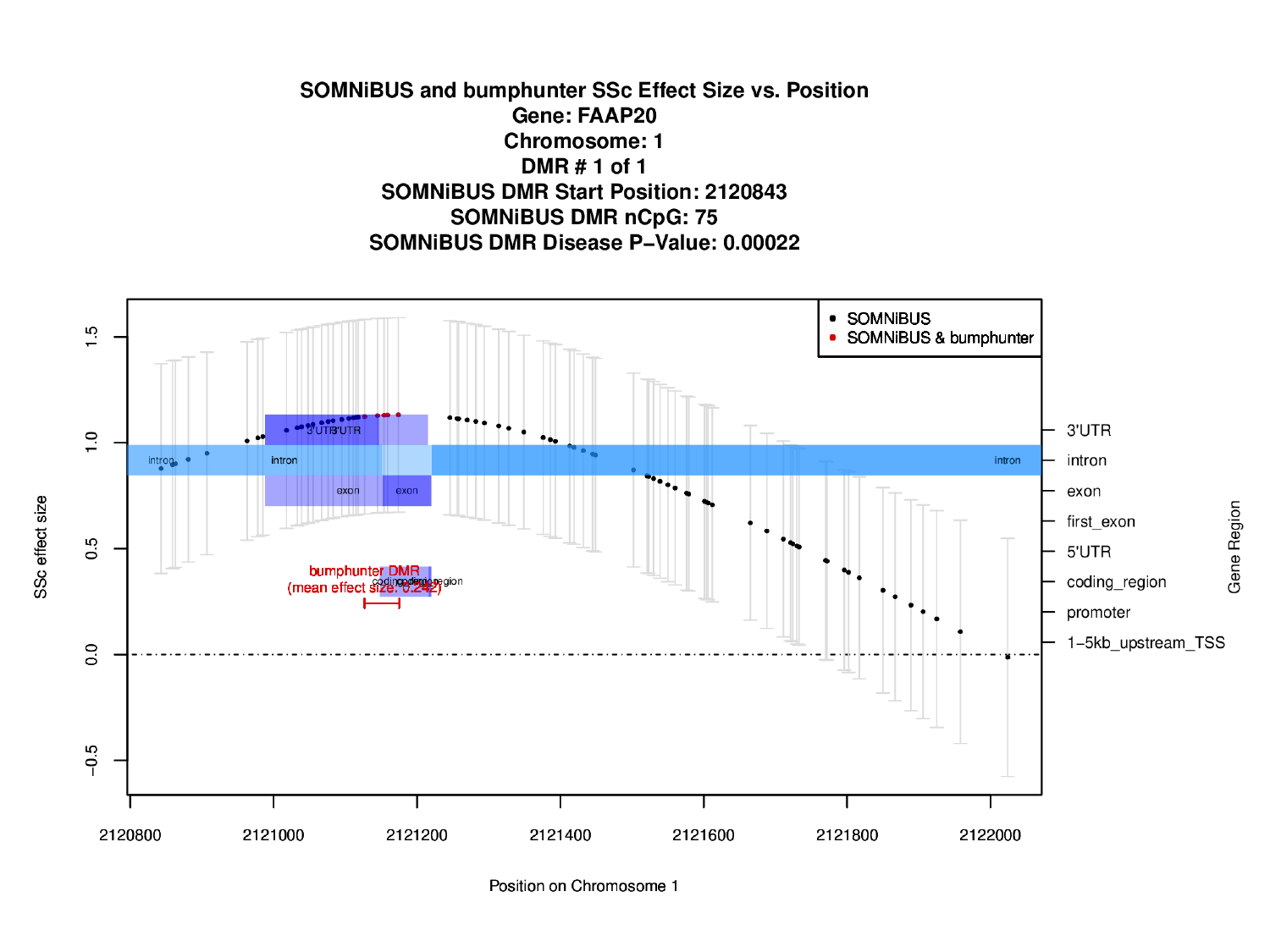

## Slide 5
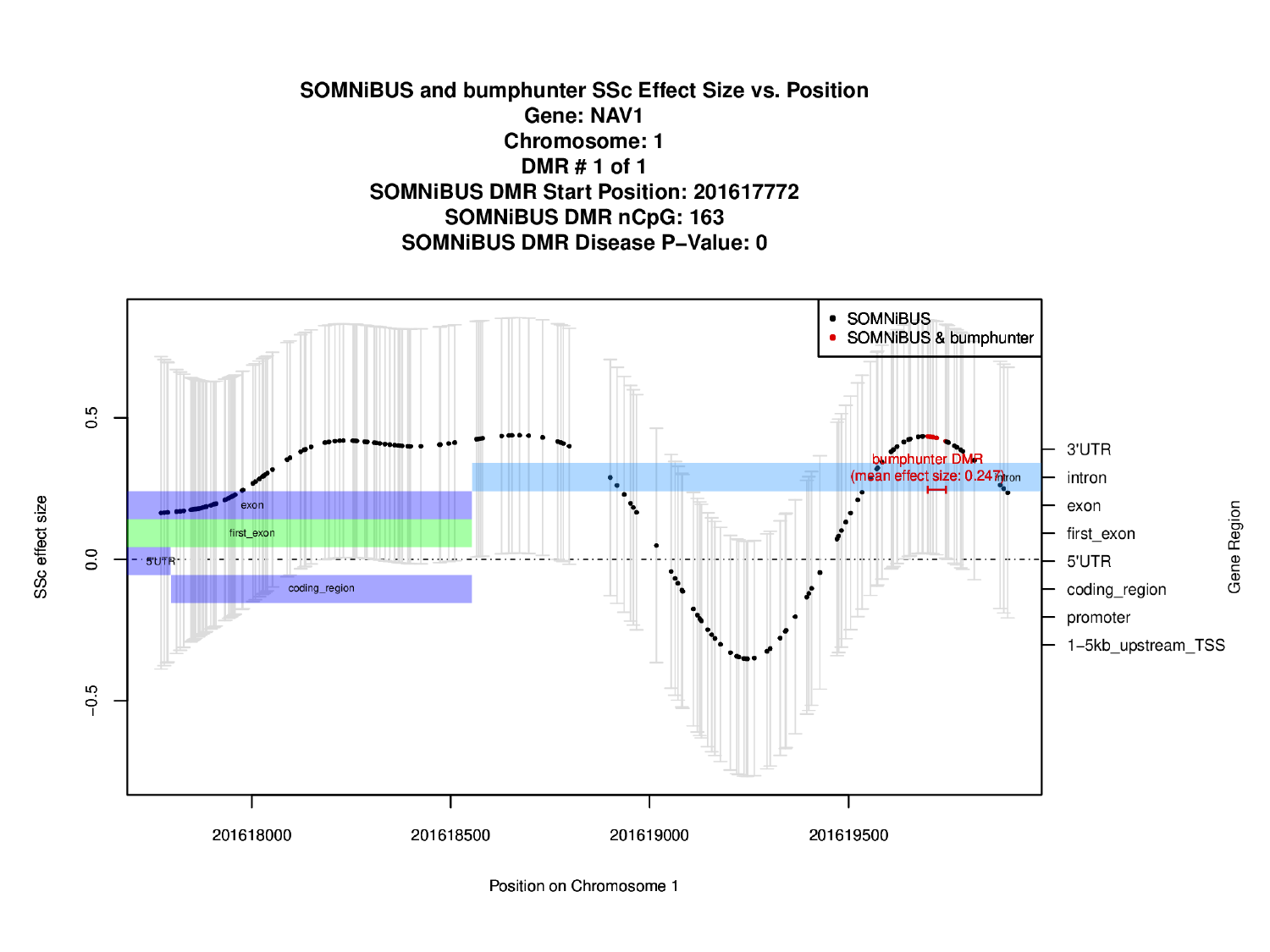

## Slide 6
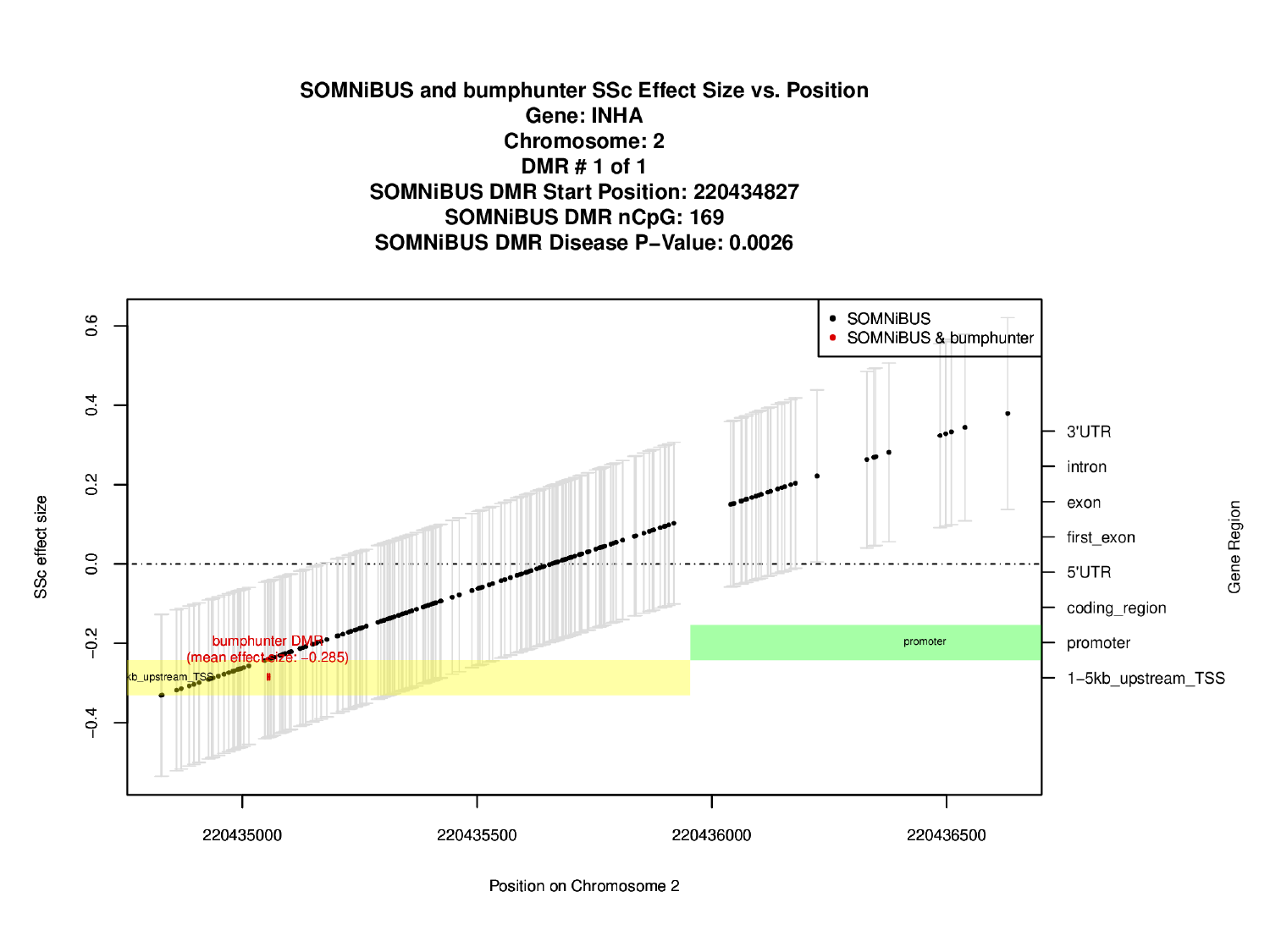

## Slide 7
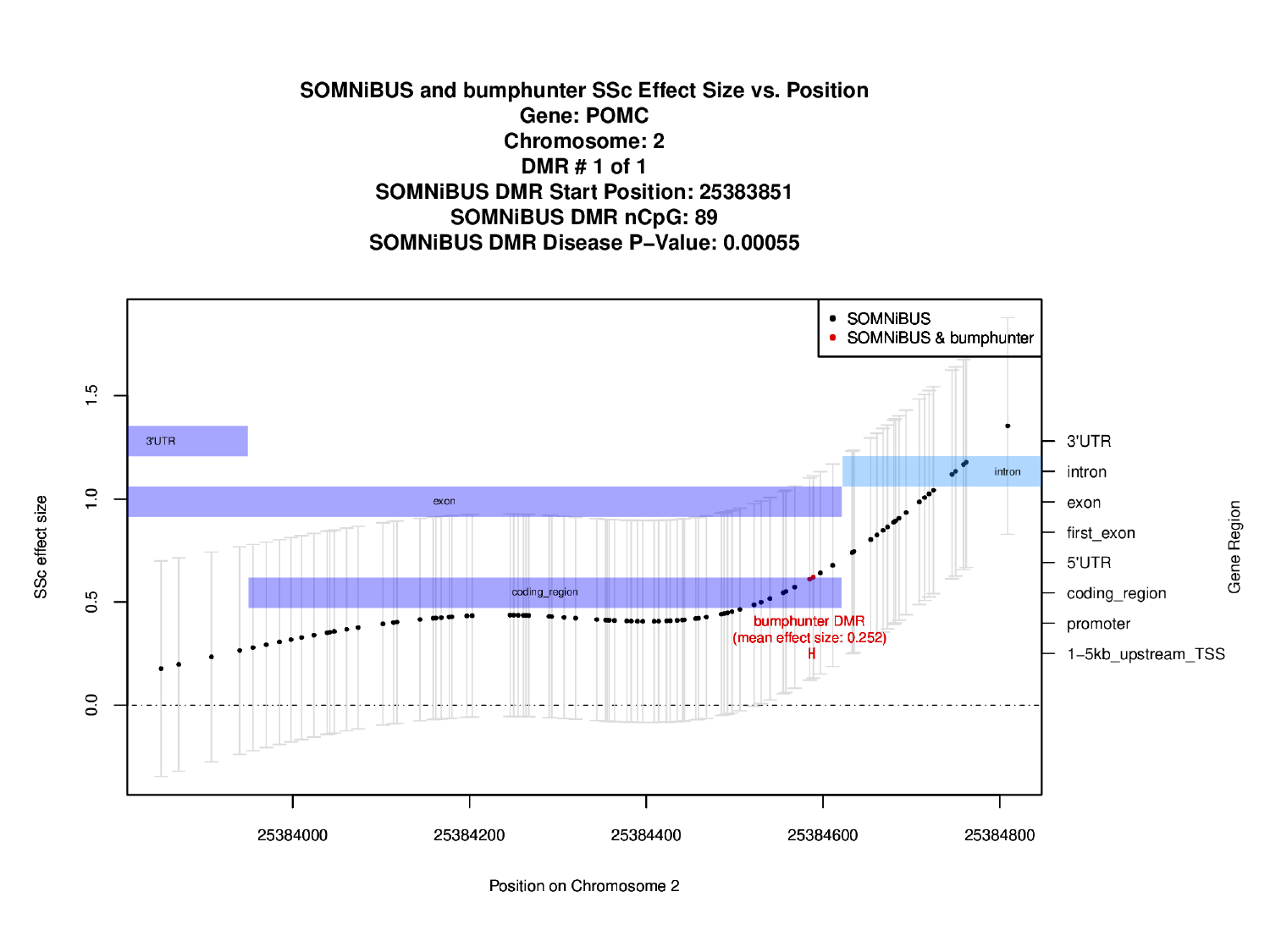

## Slide 8
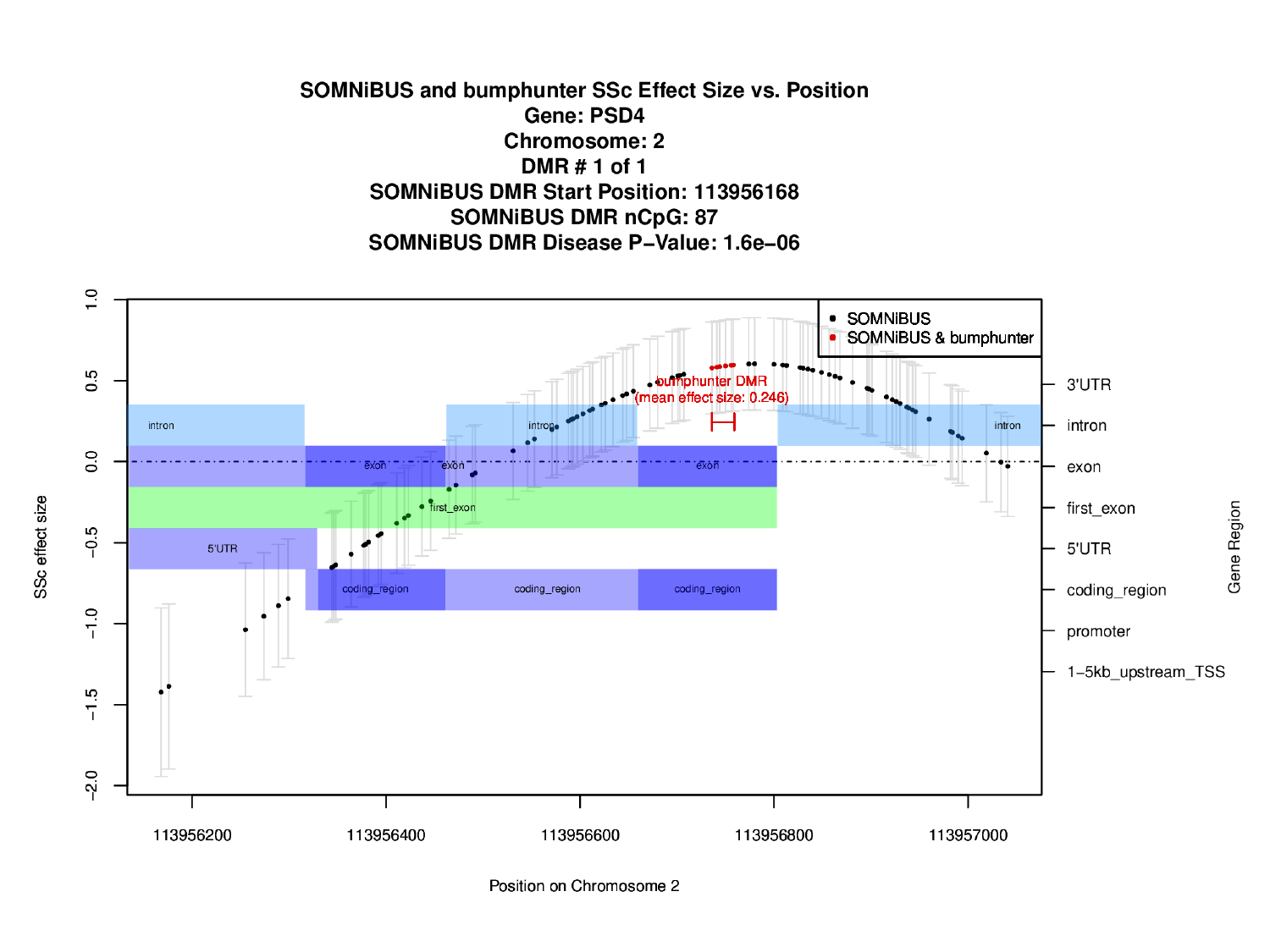

## Slide 9
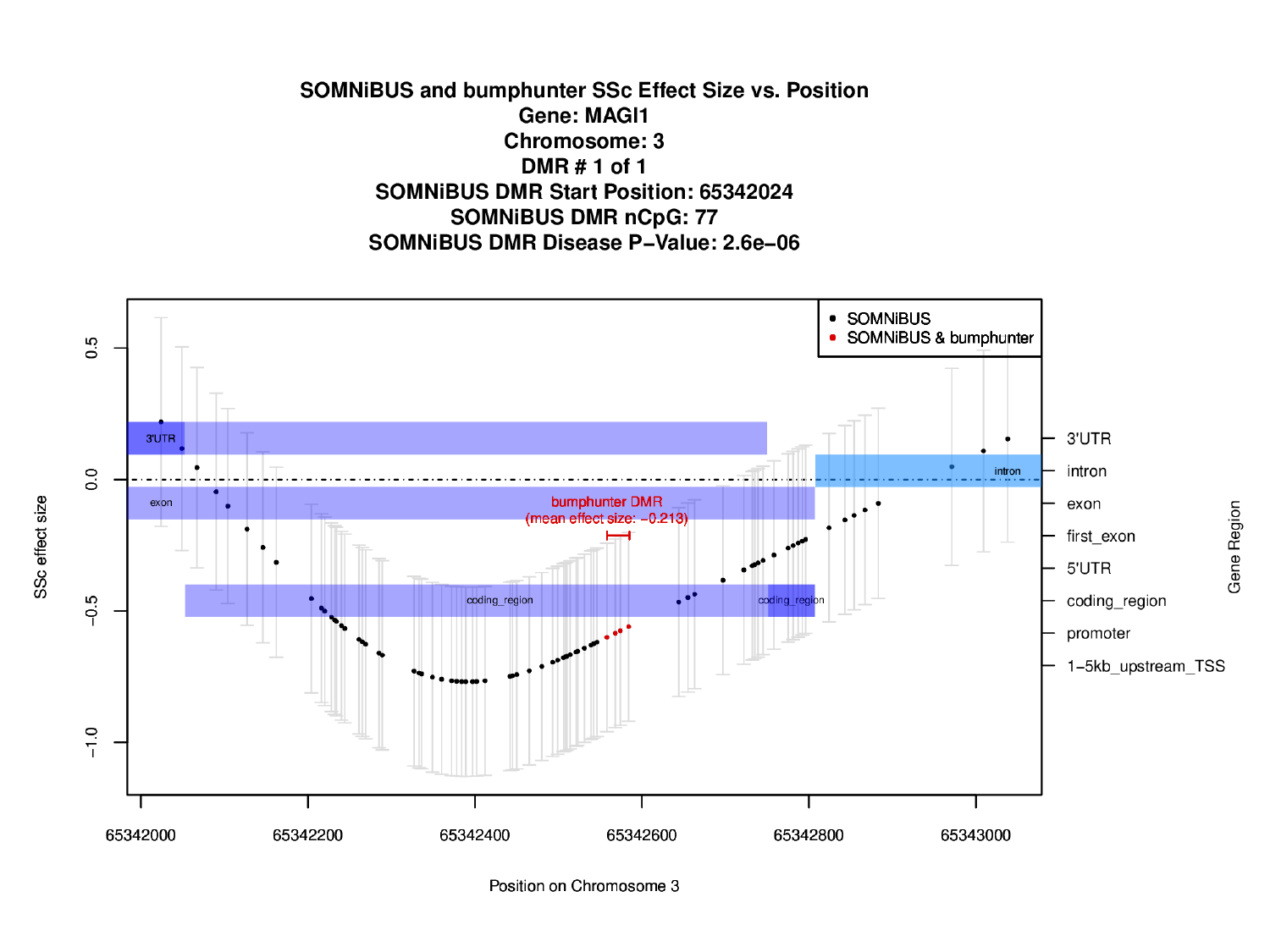

## Slide 10
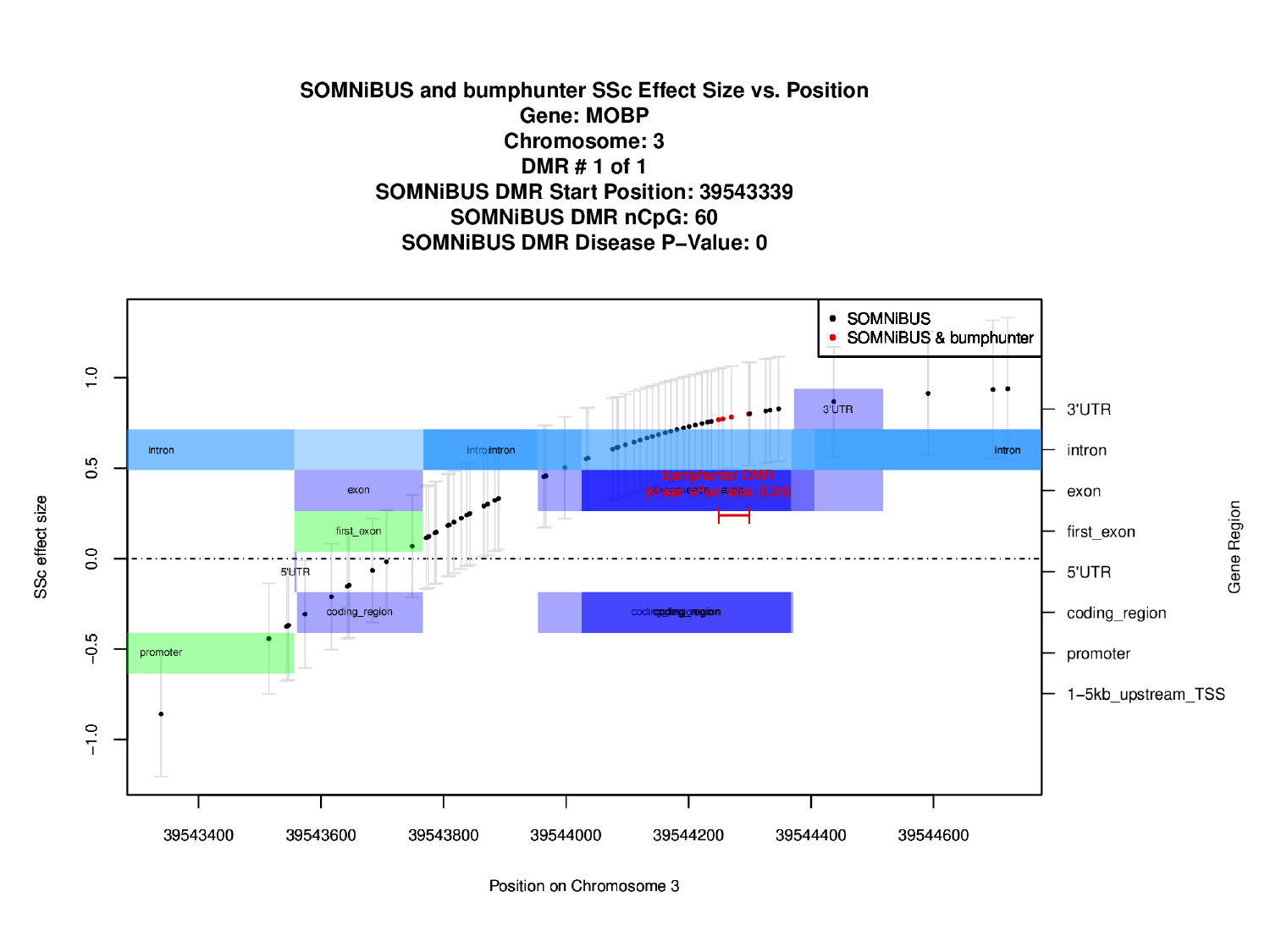

## Slide 11
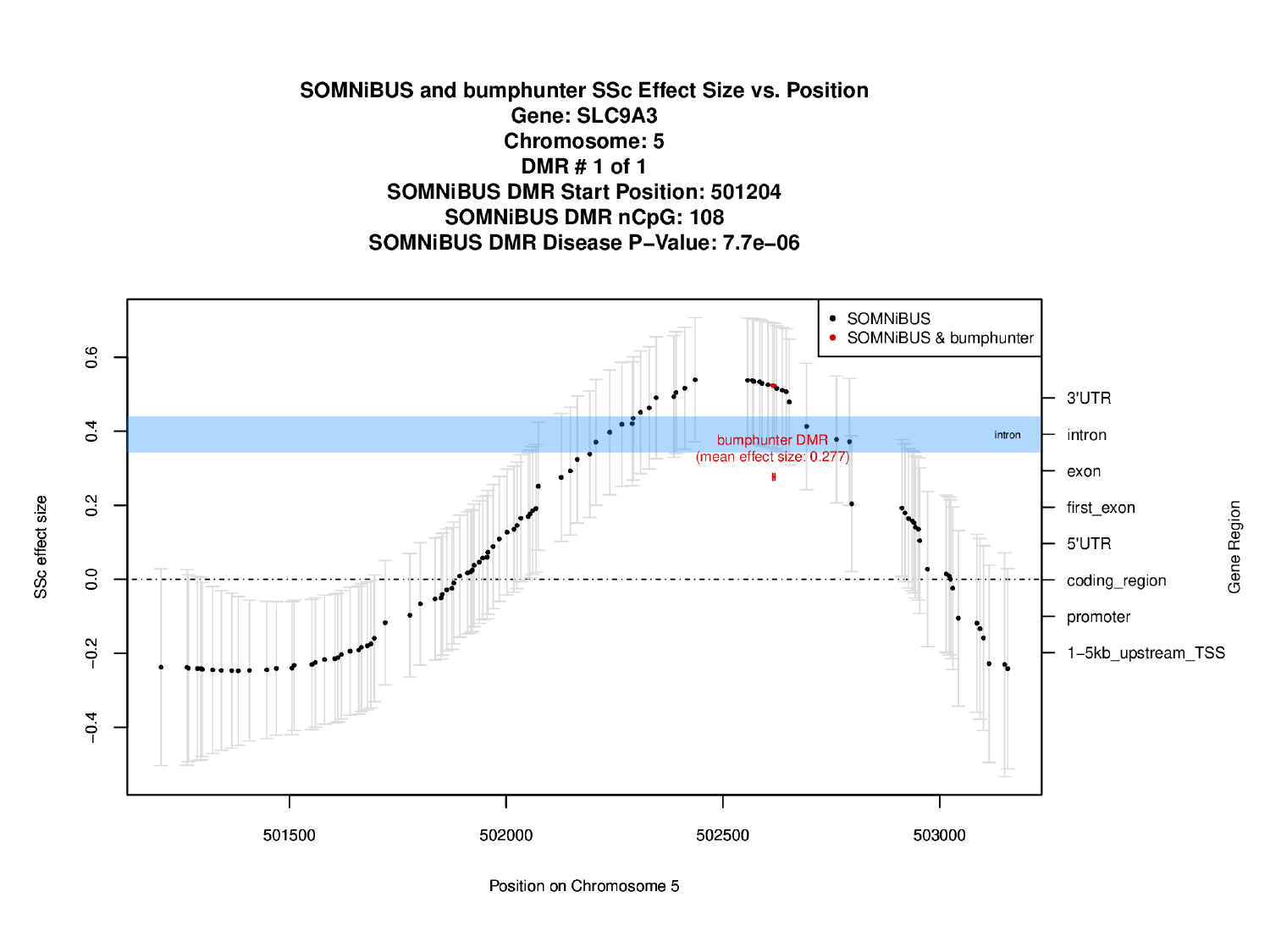

## Slide 12
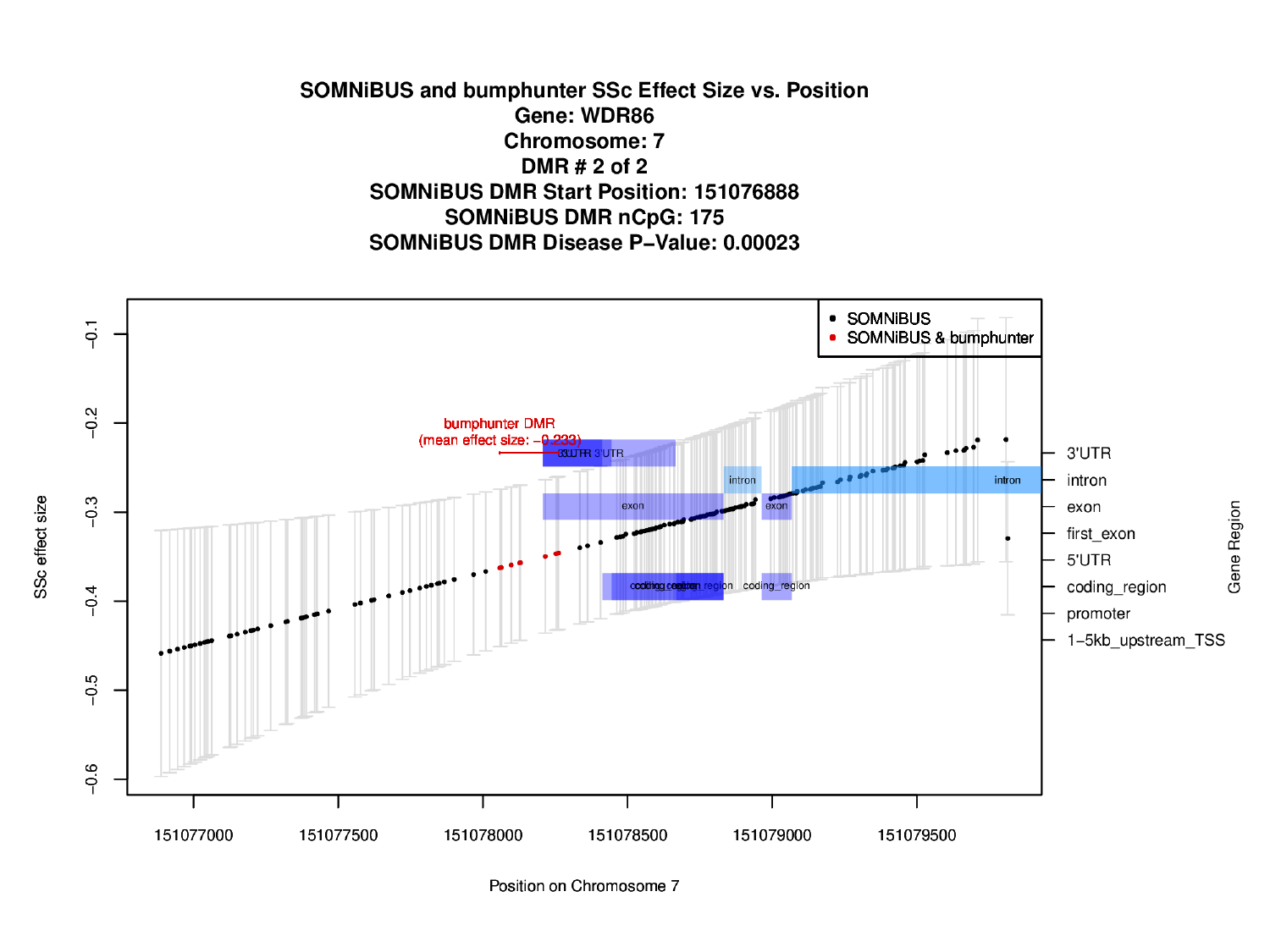

## Slide 13
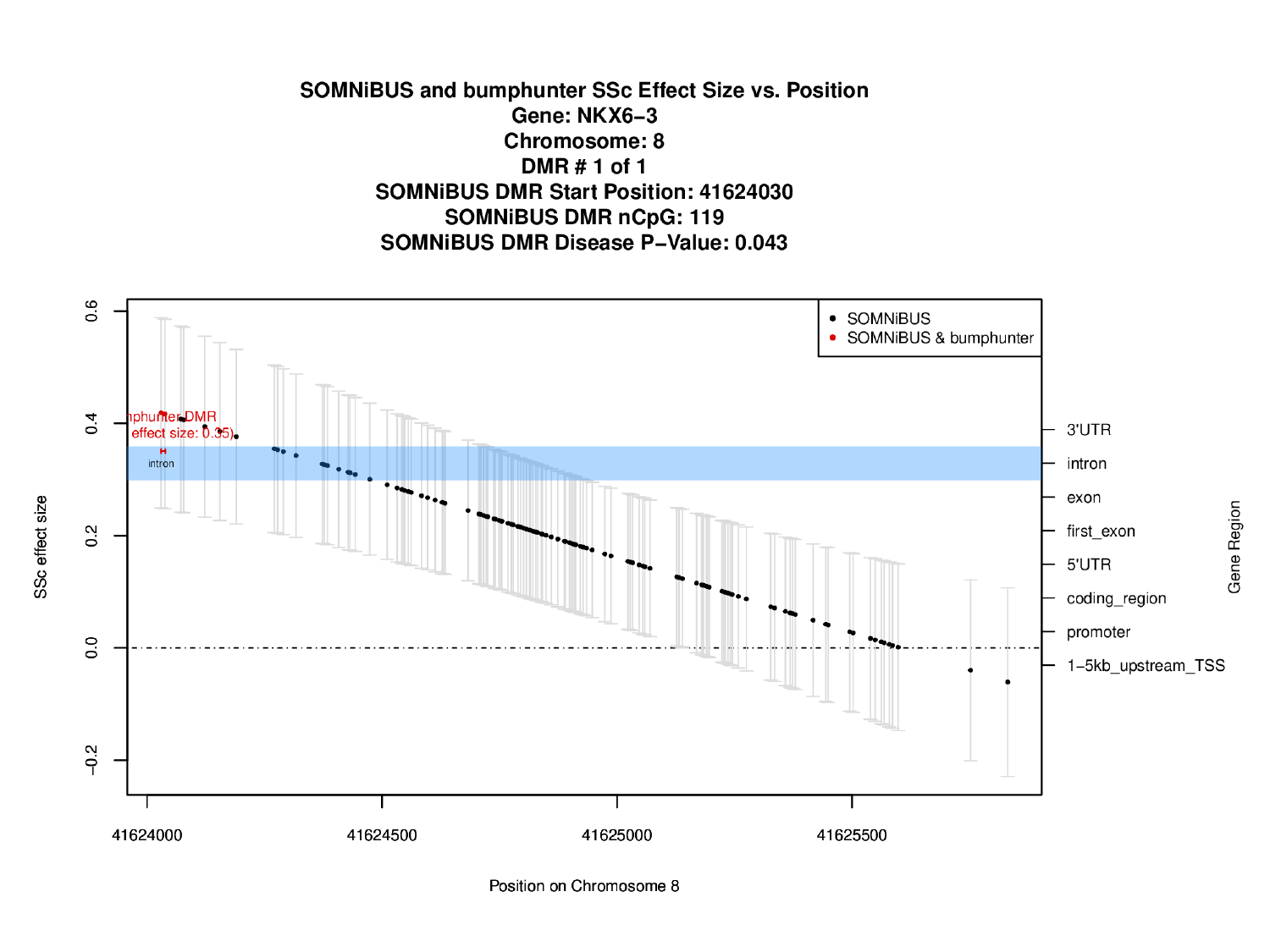

## Slide 14
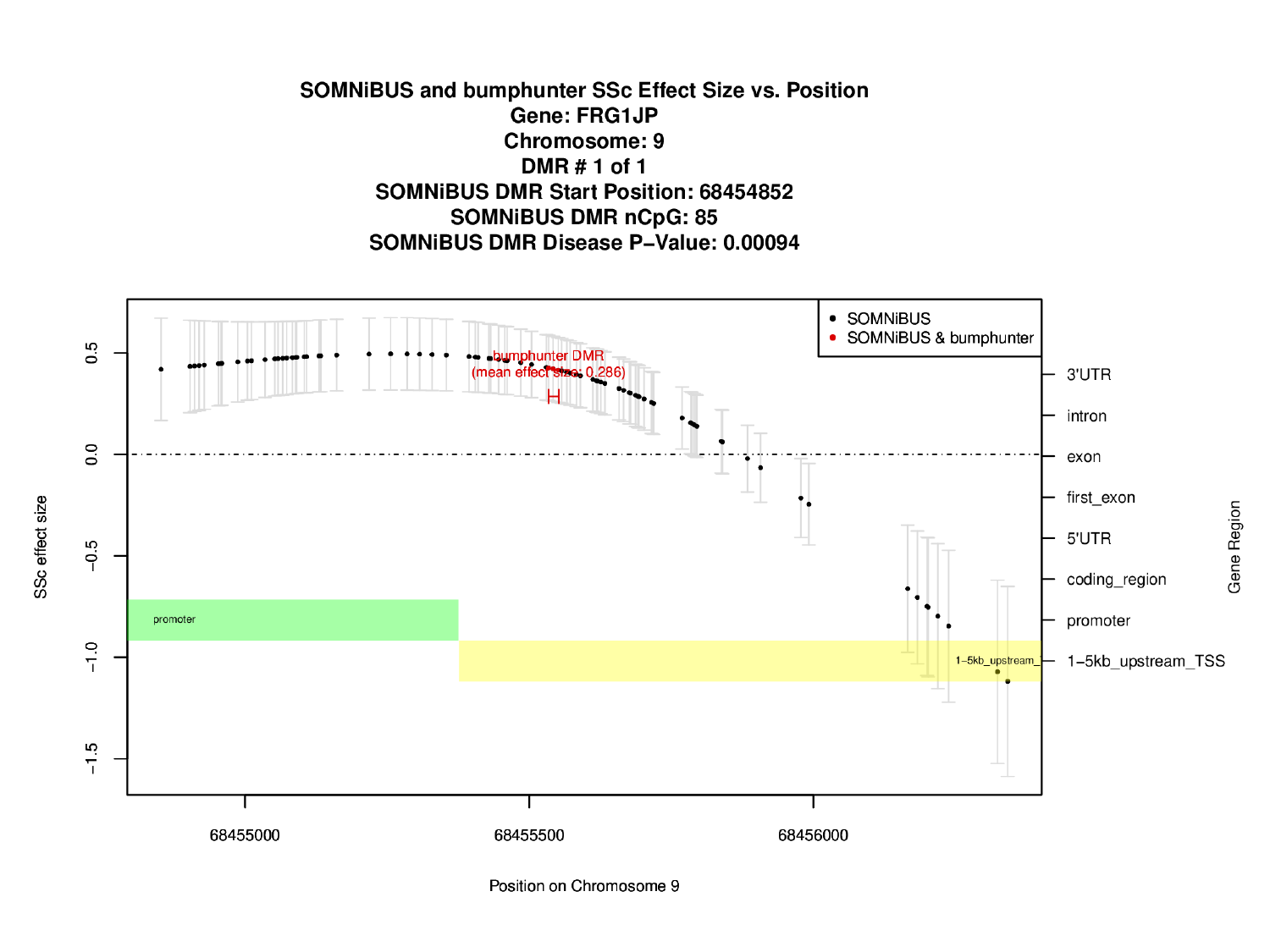

## Slide 15
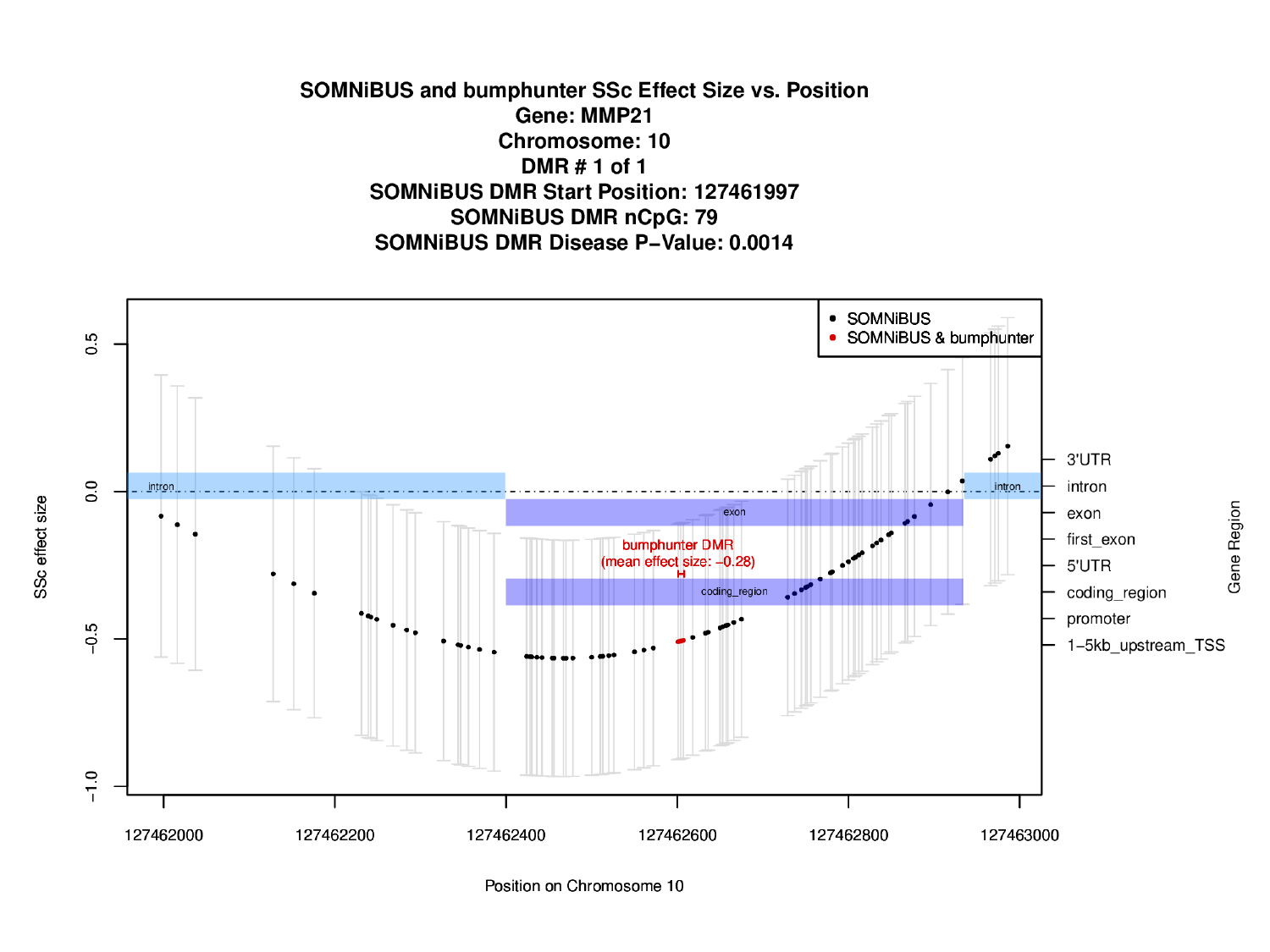

## Slide 16
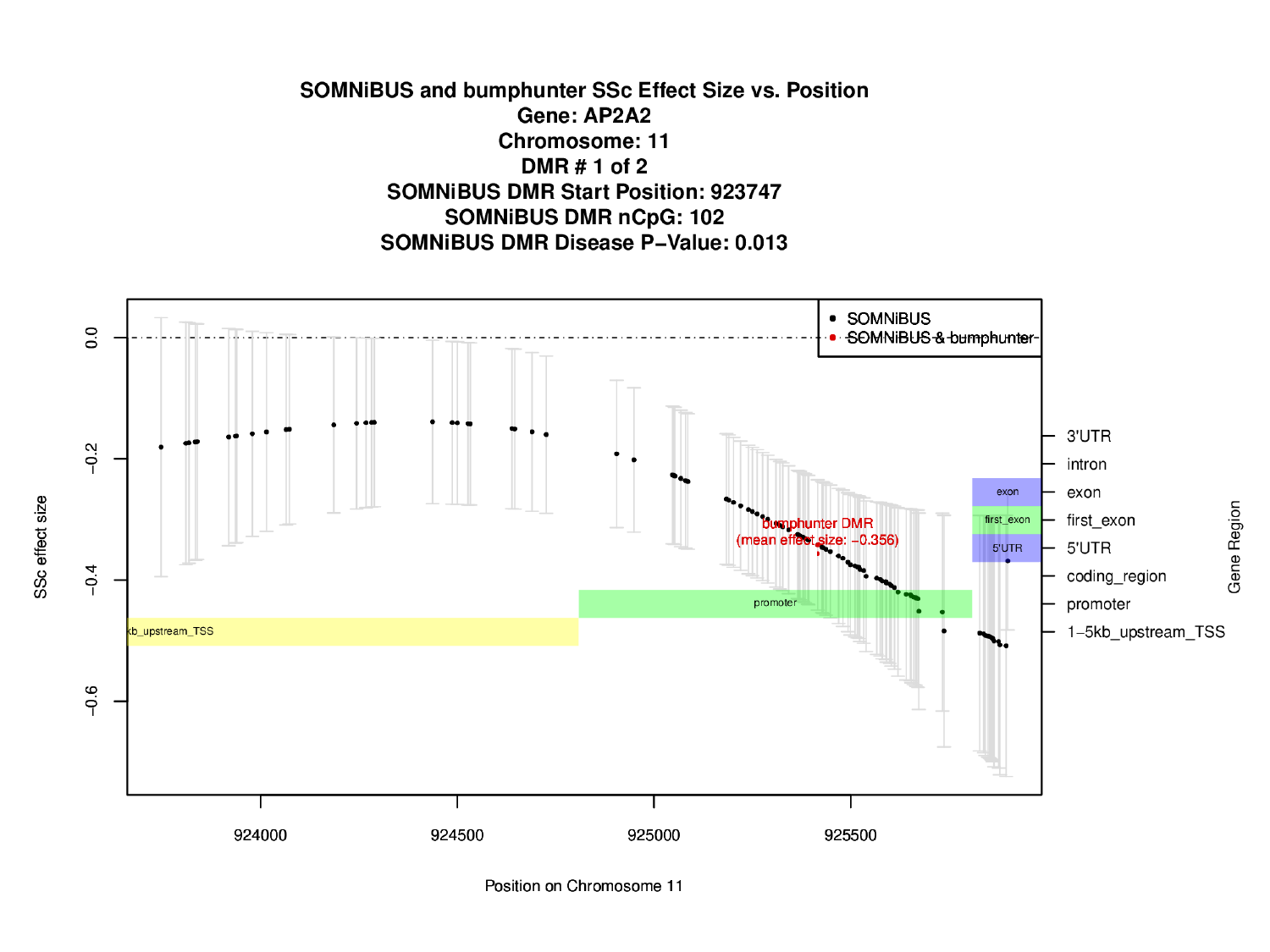

## Slide 17
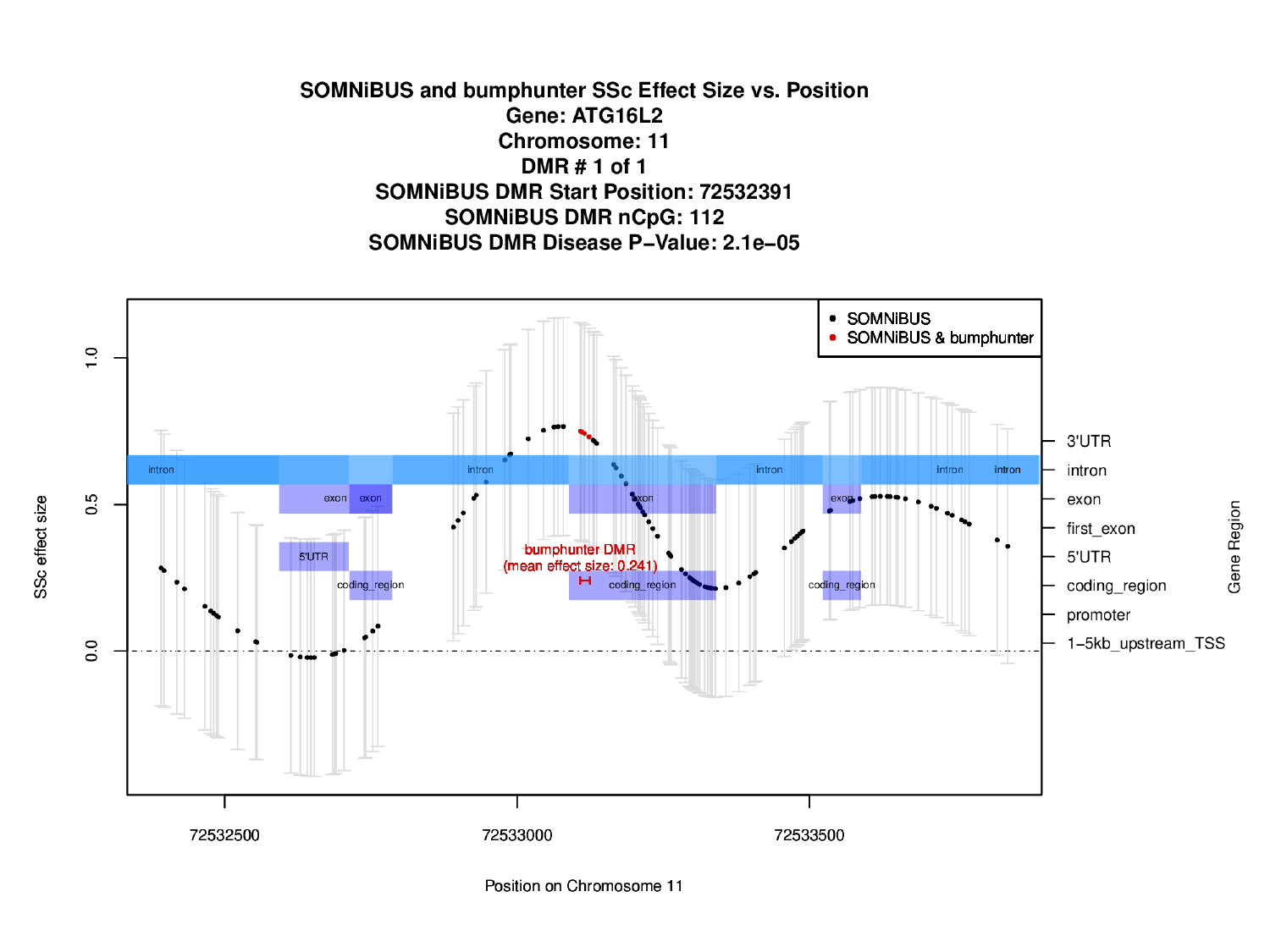

## Slide 18
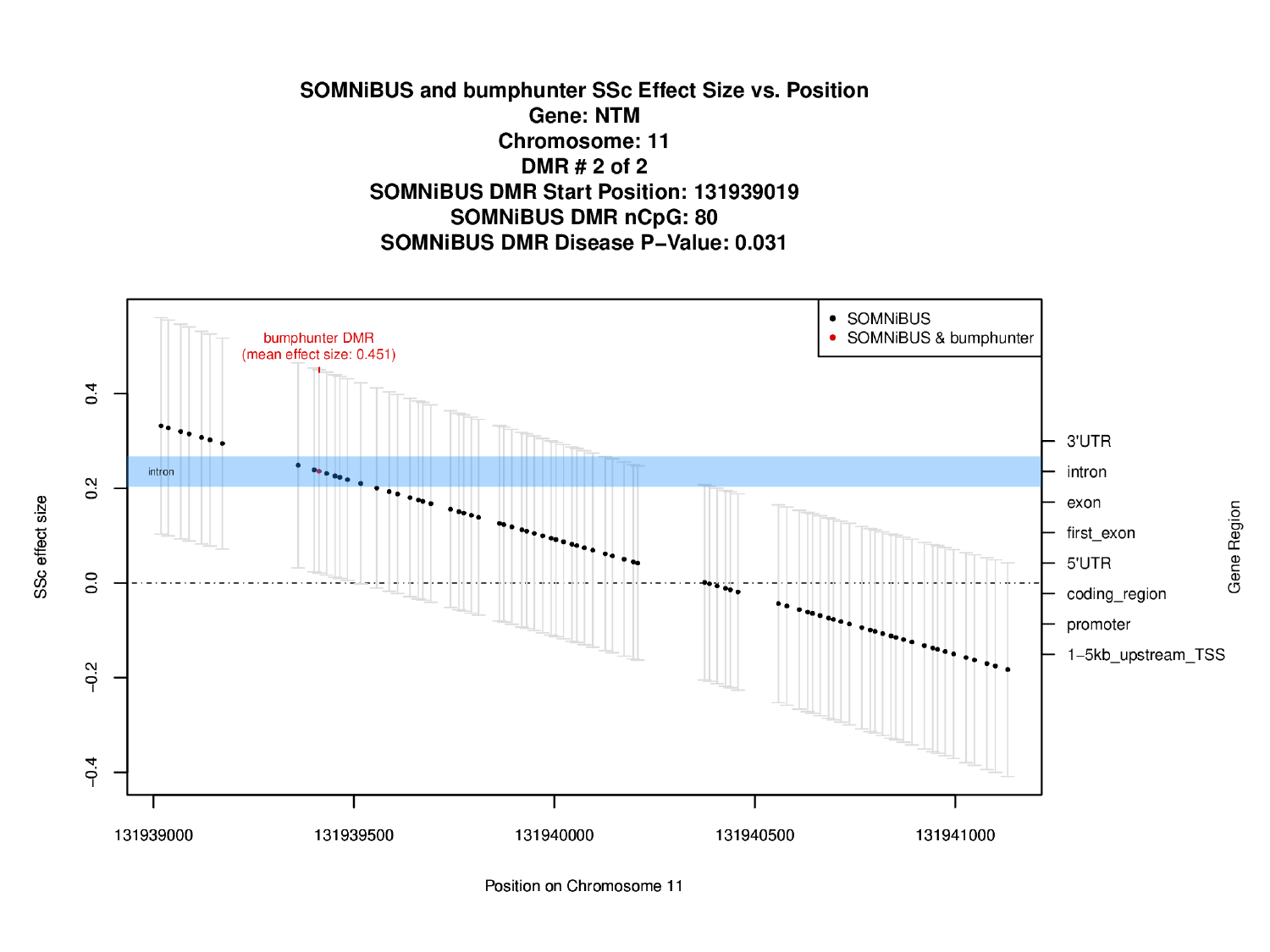

## Slide 19
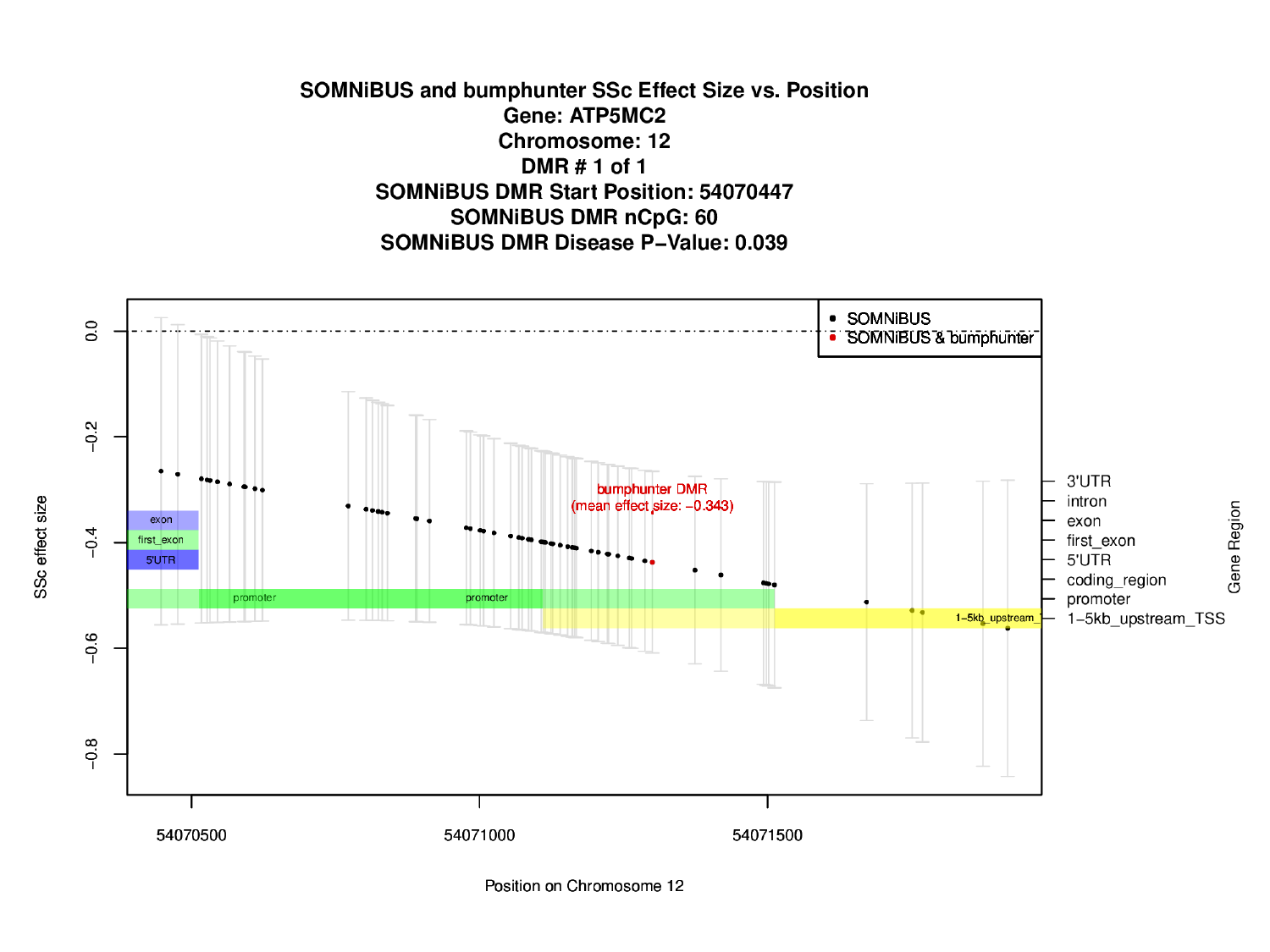

## Slide 20
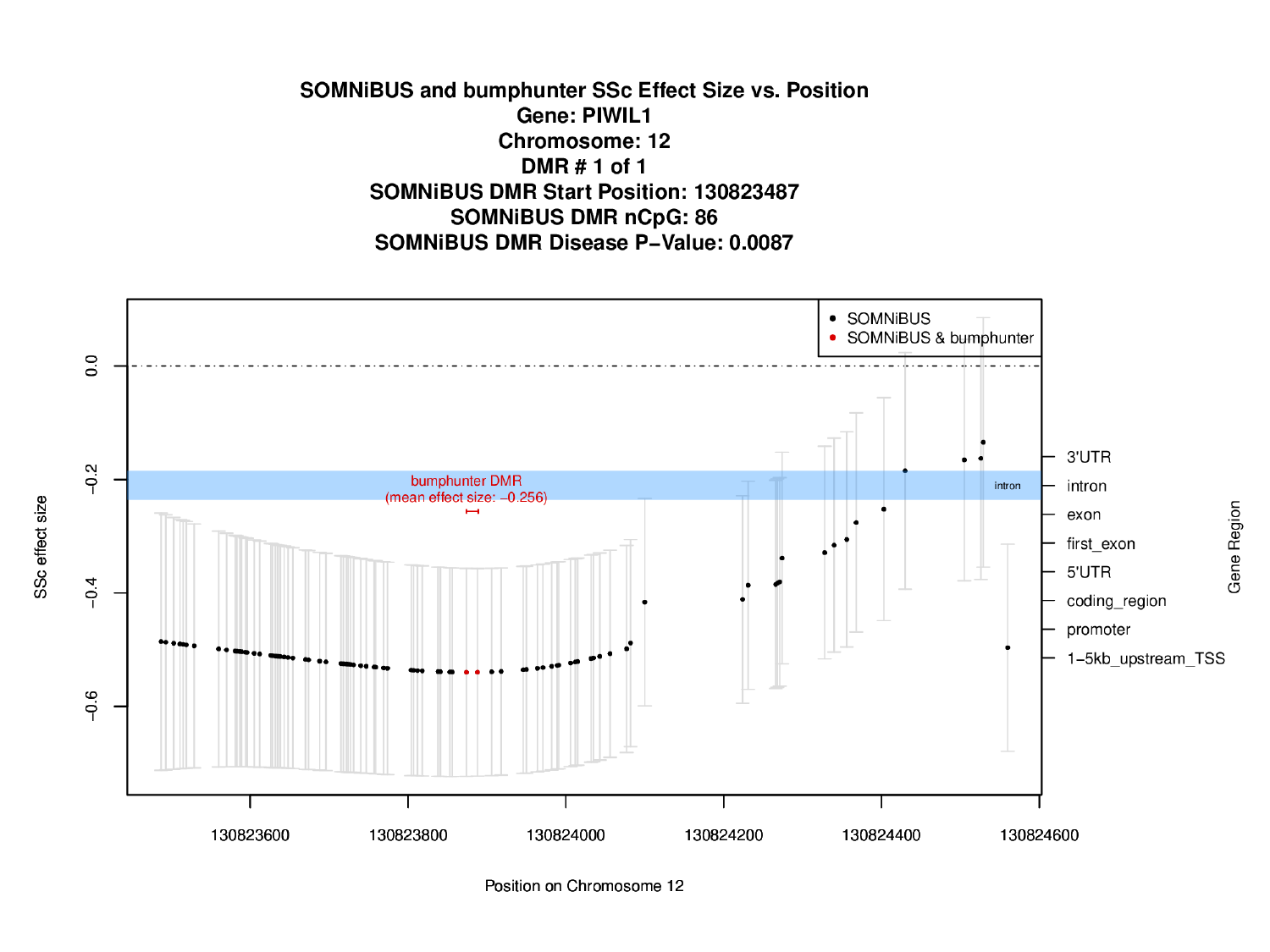

## Slide 21
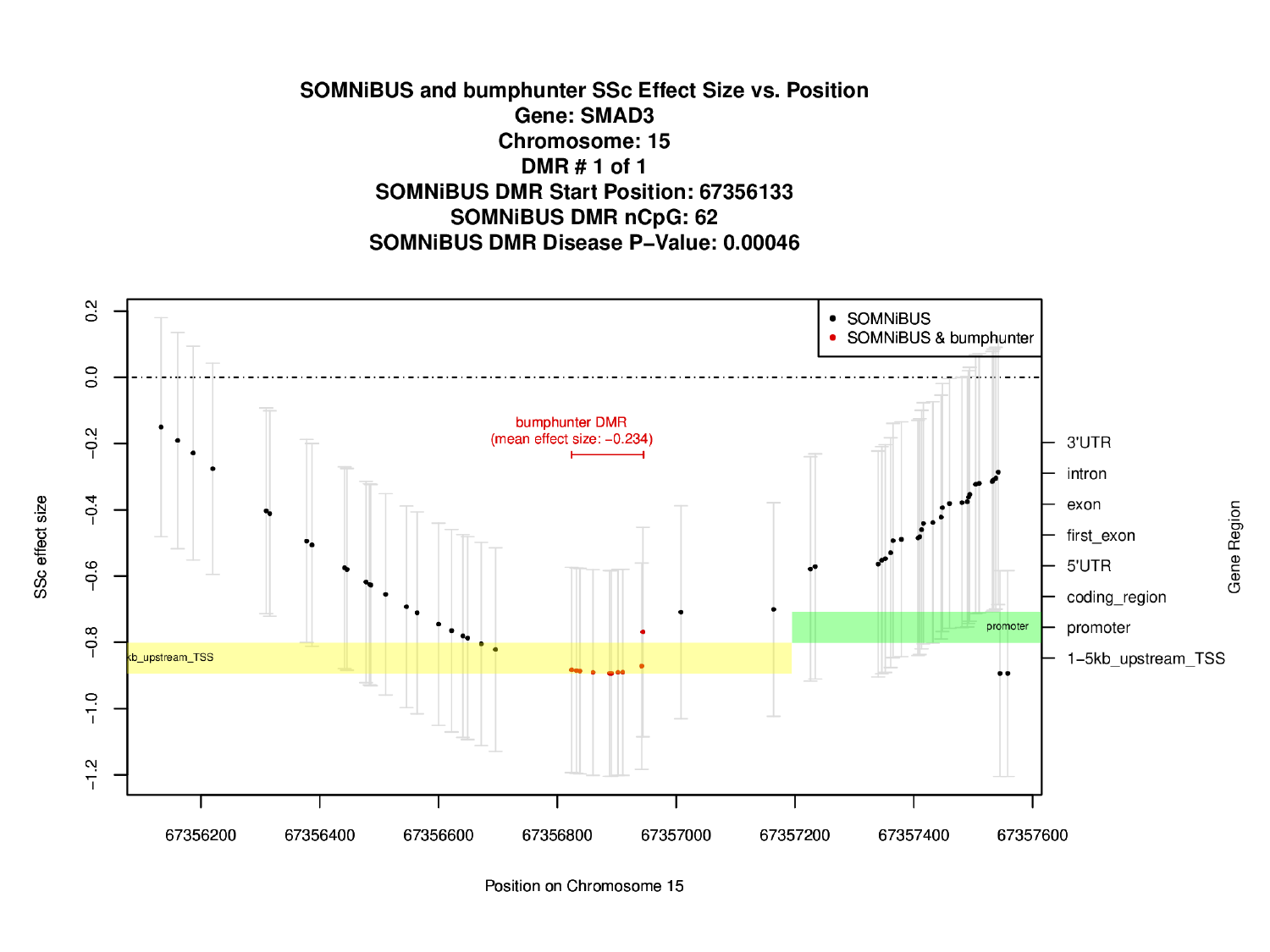

## Slide 22
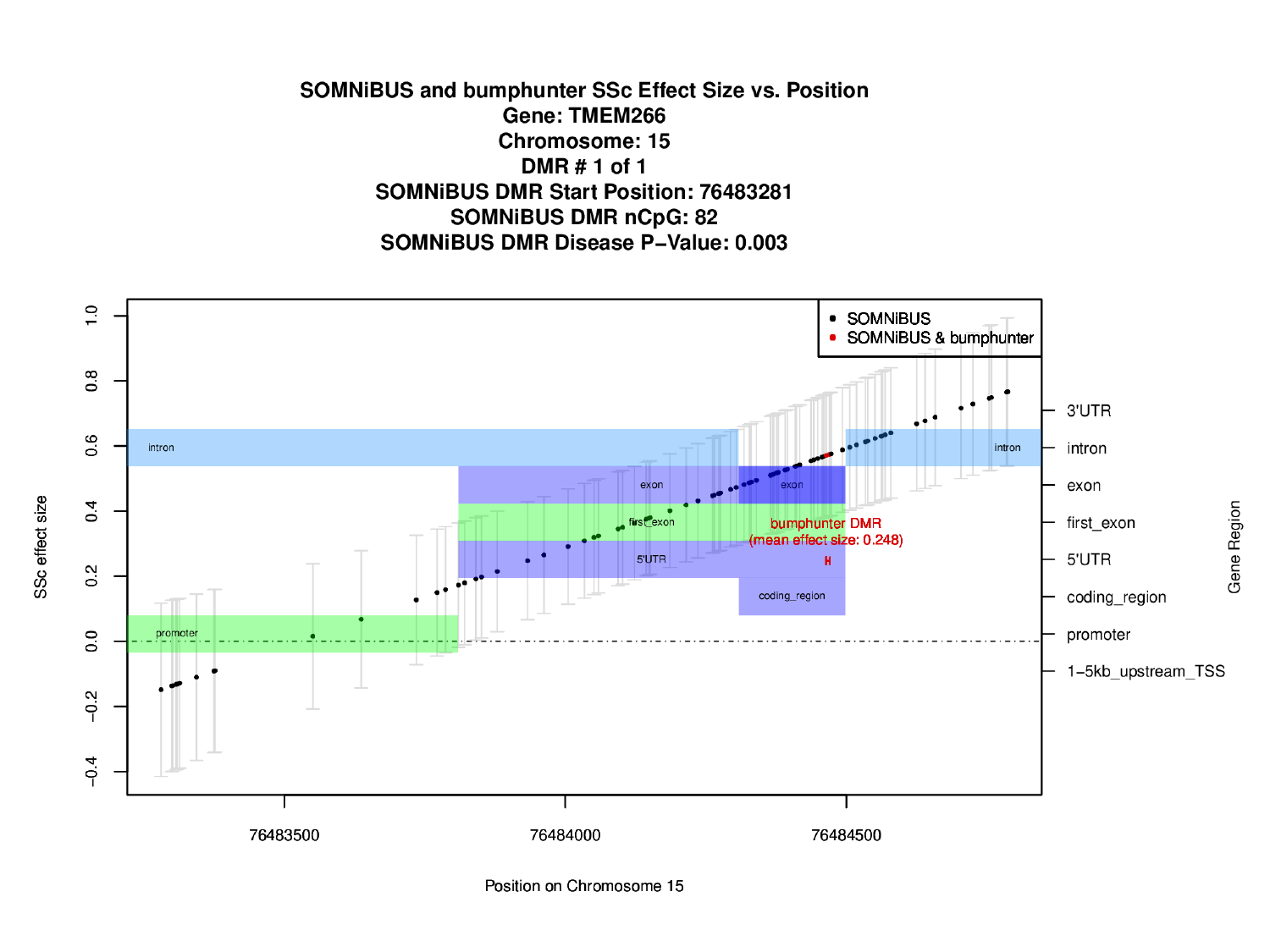

## Slide 23
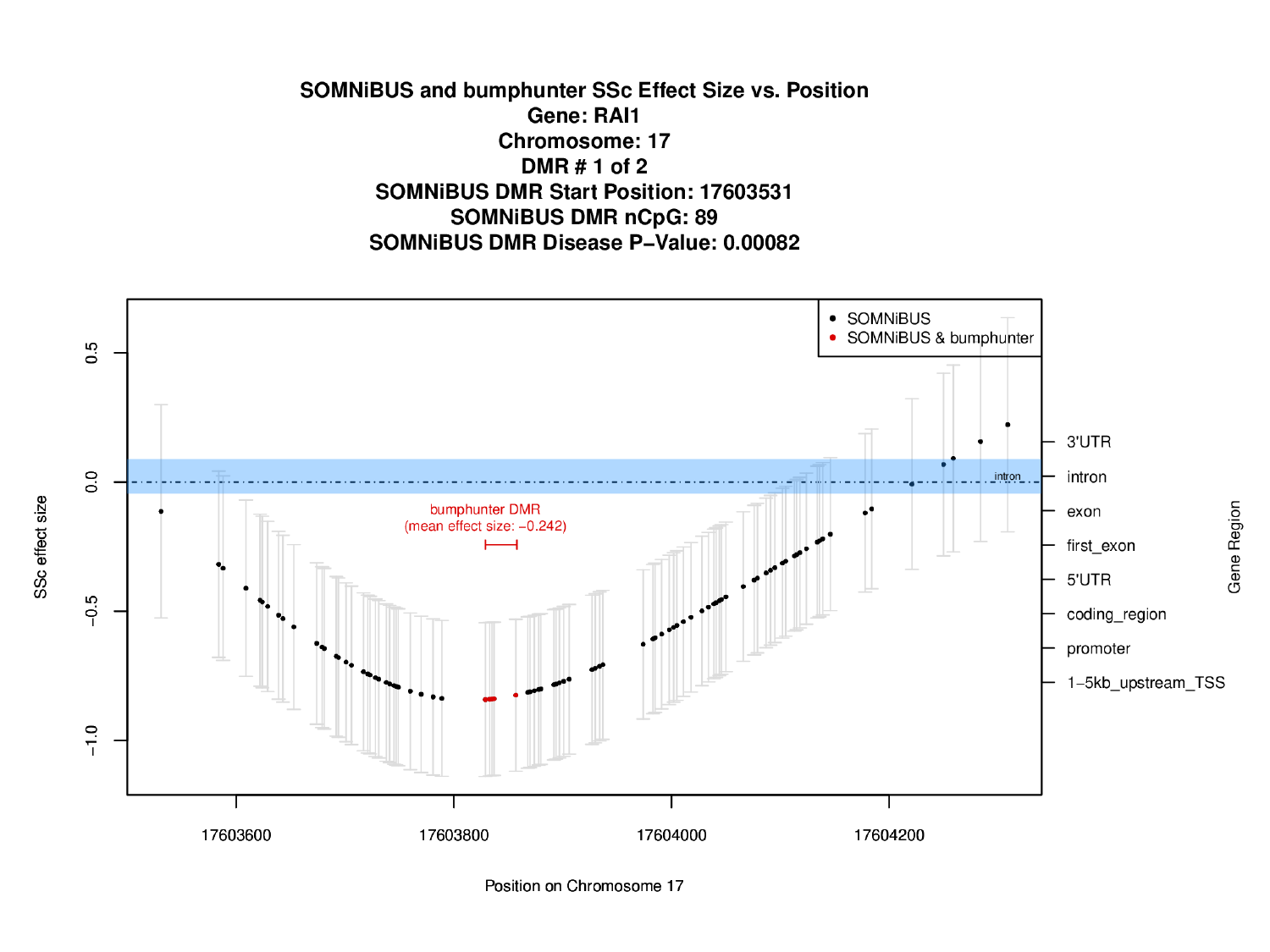

## Slide 24
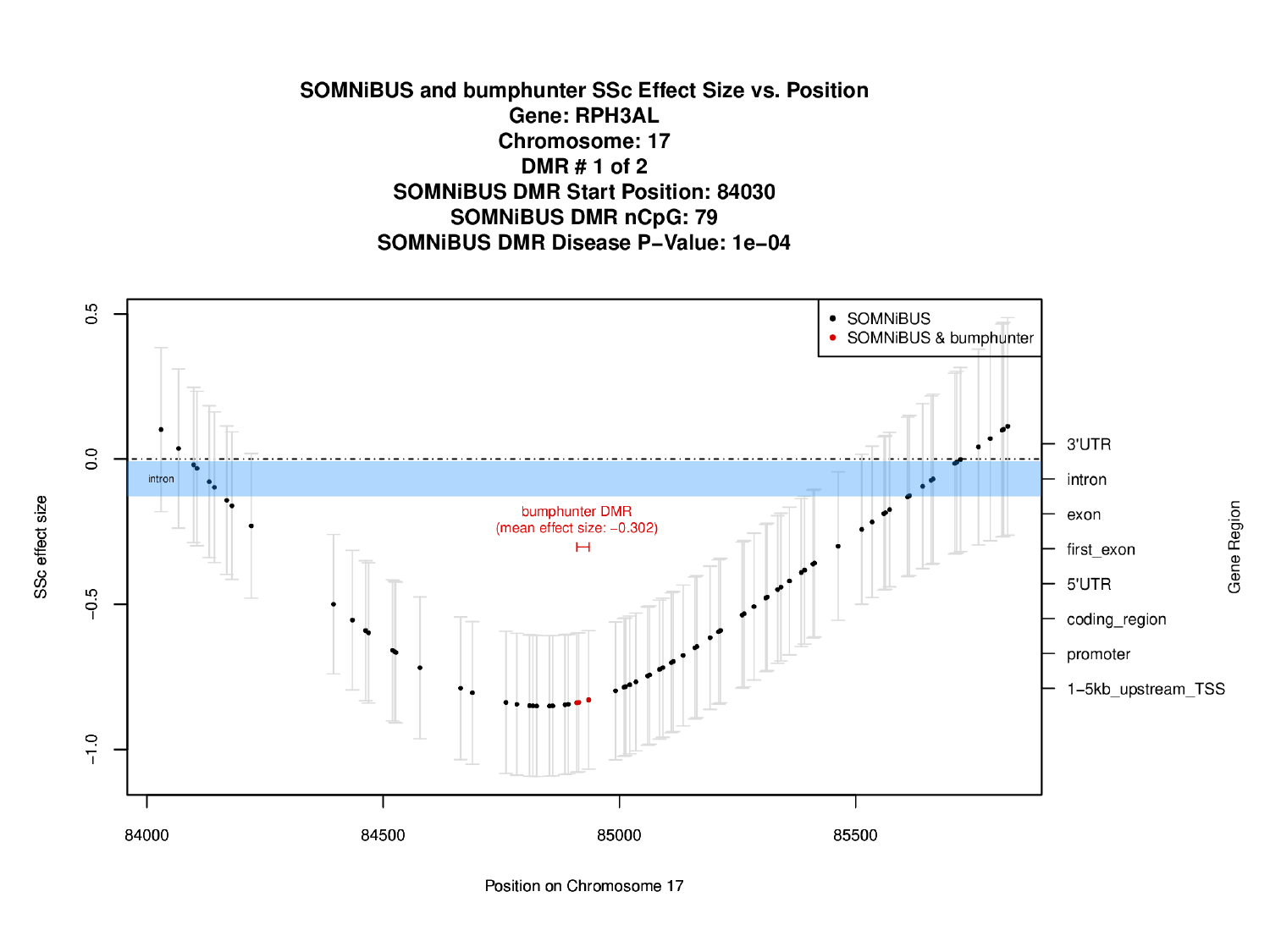

## Slide 25
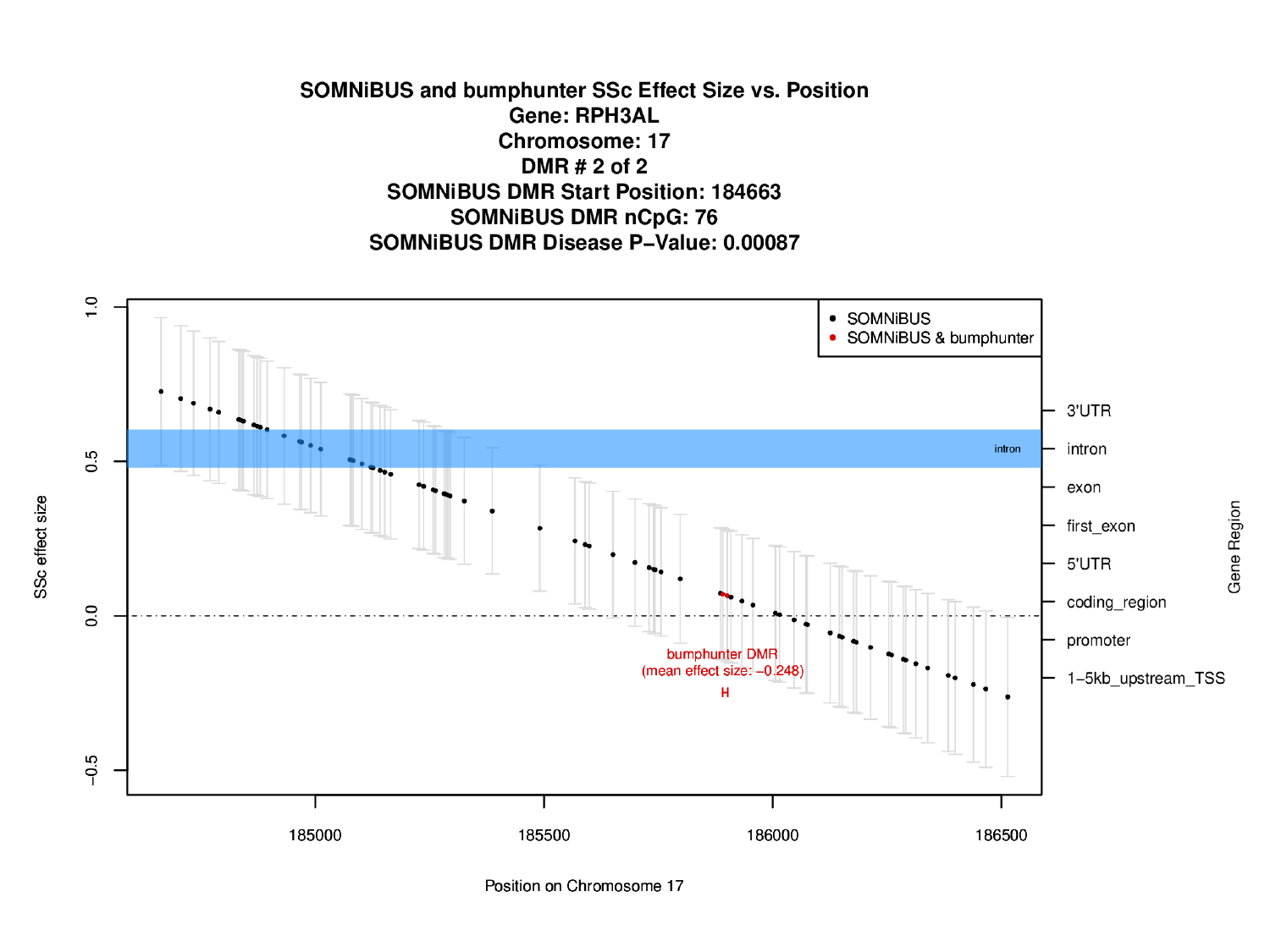

## Slide 26
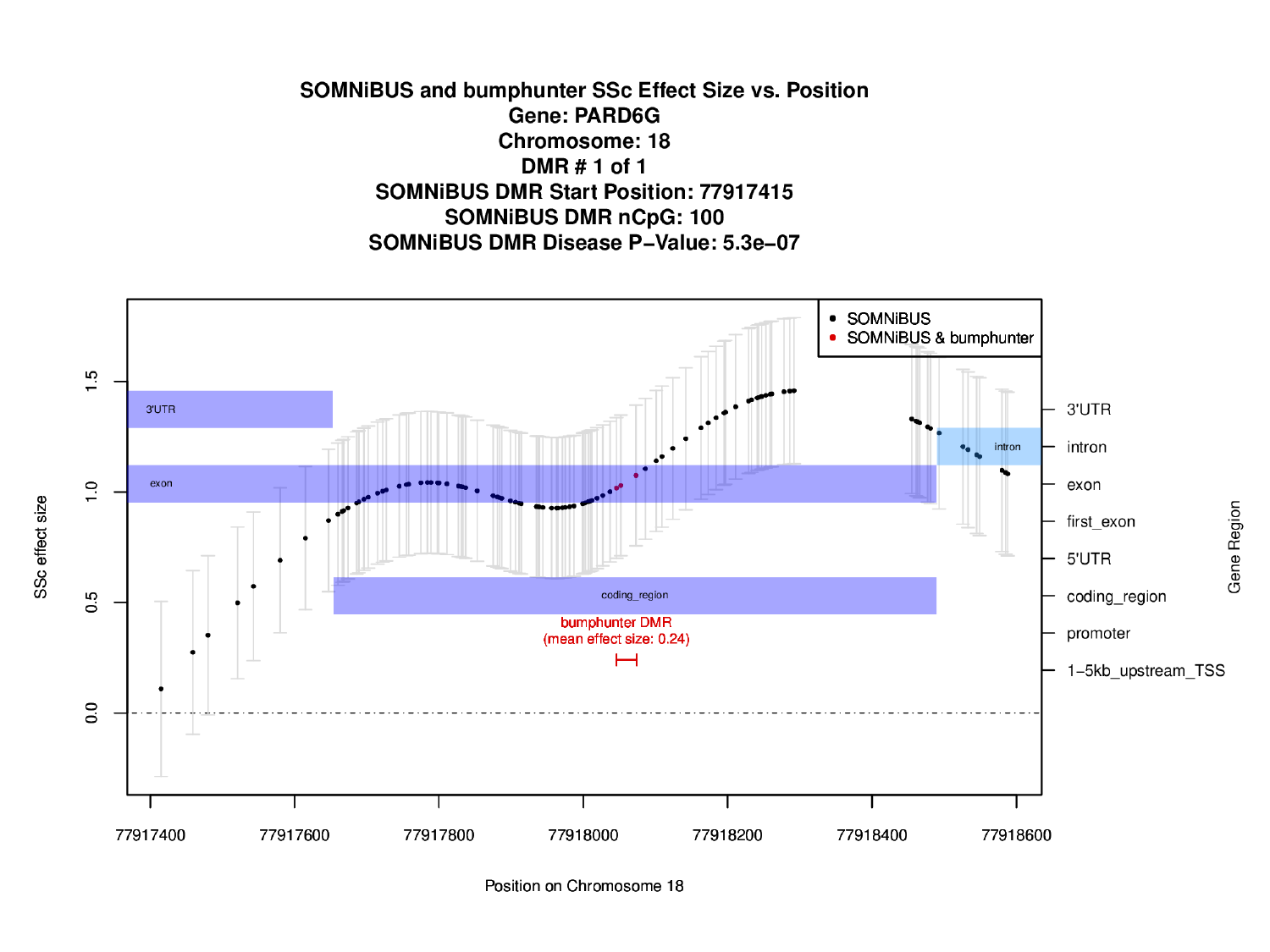

## Slide 27
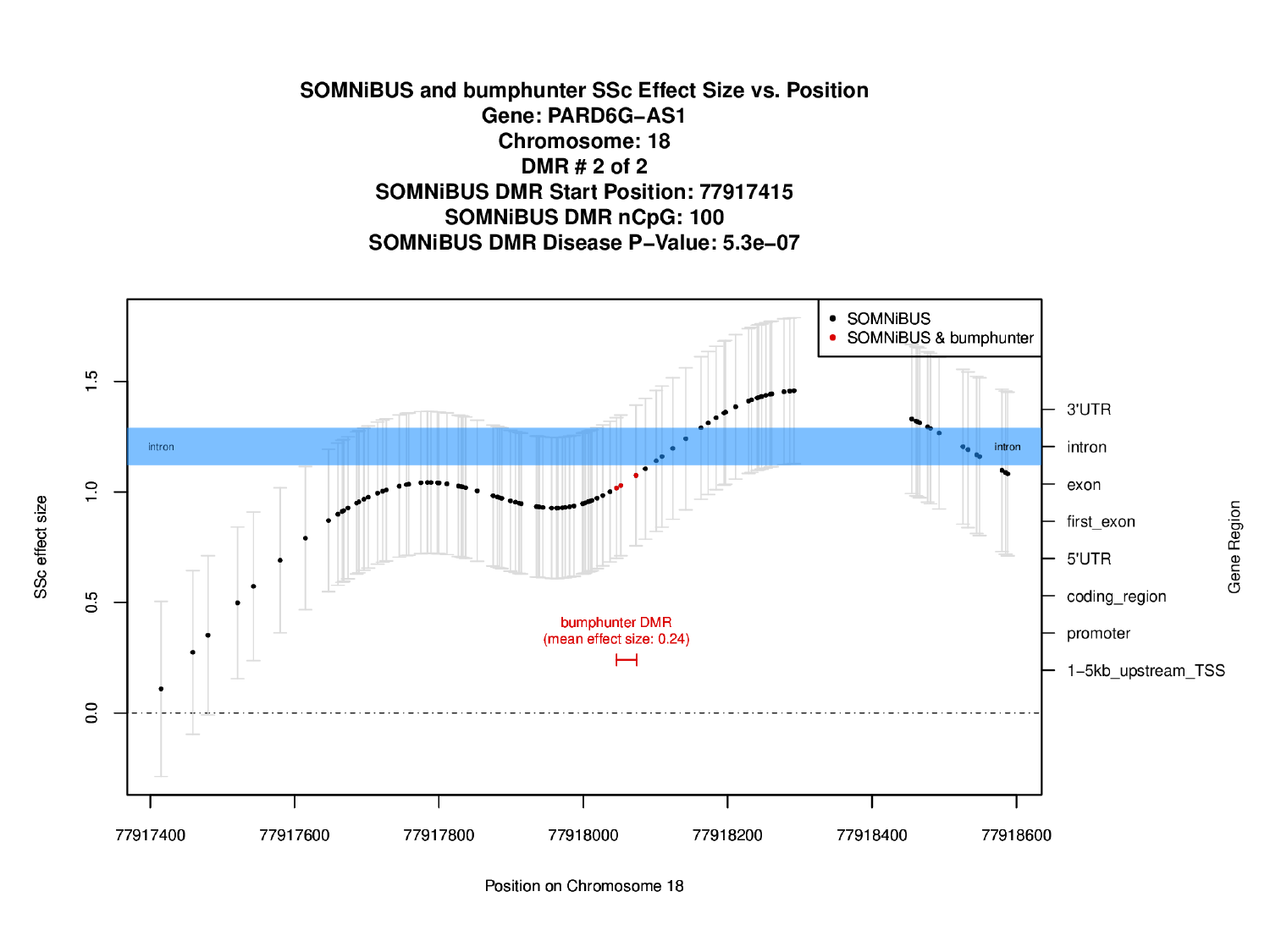

## Slide 28
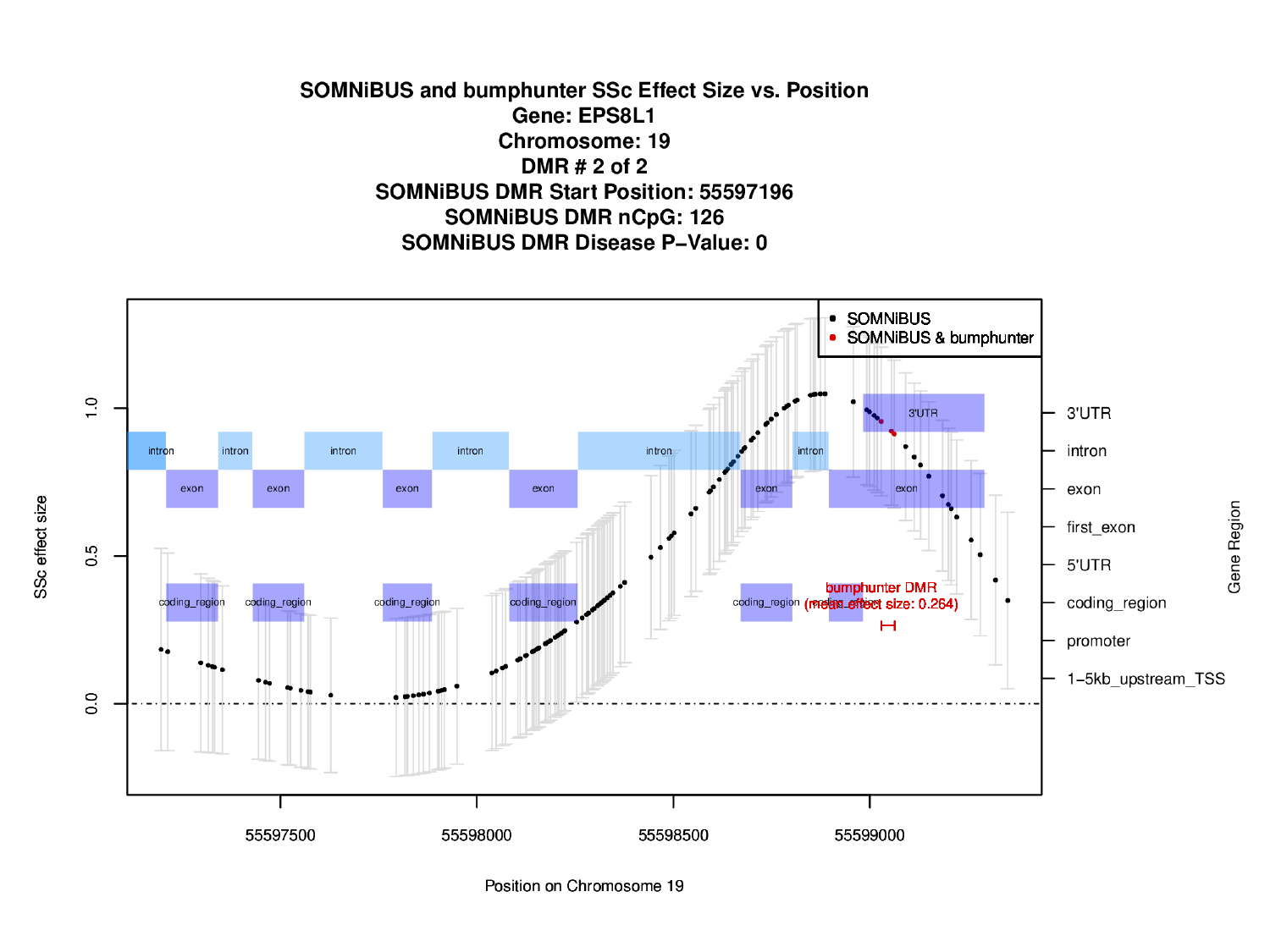

## Slide 29
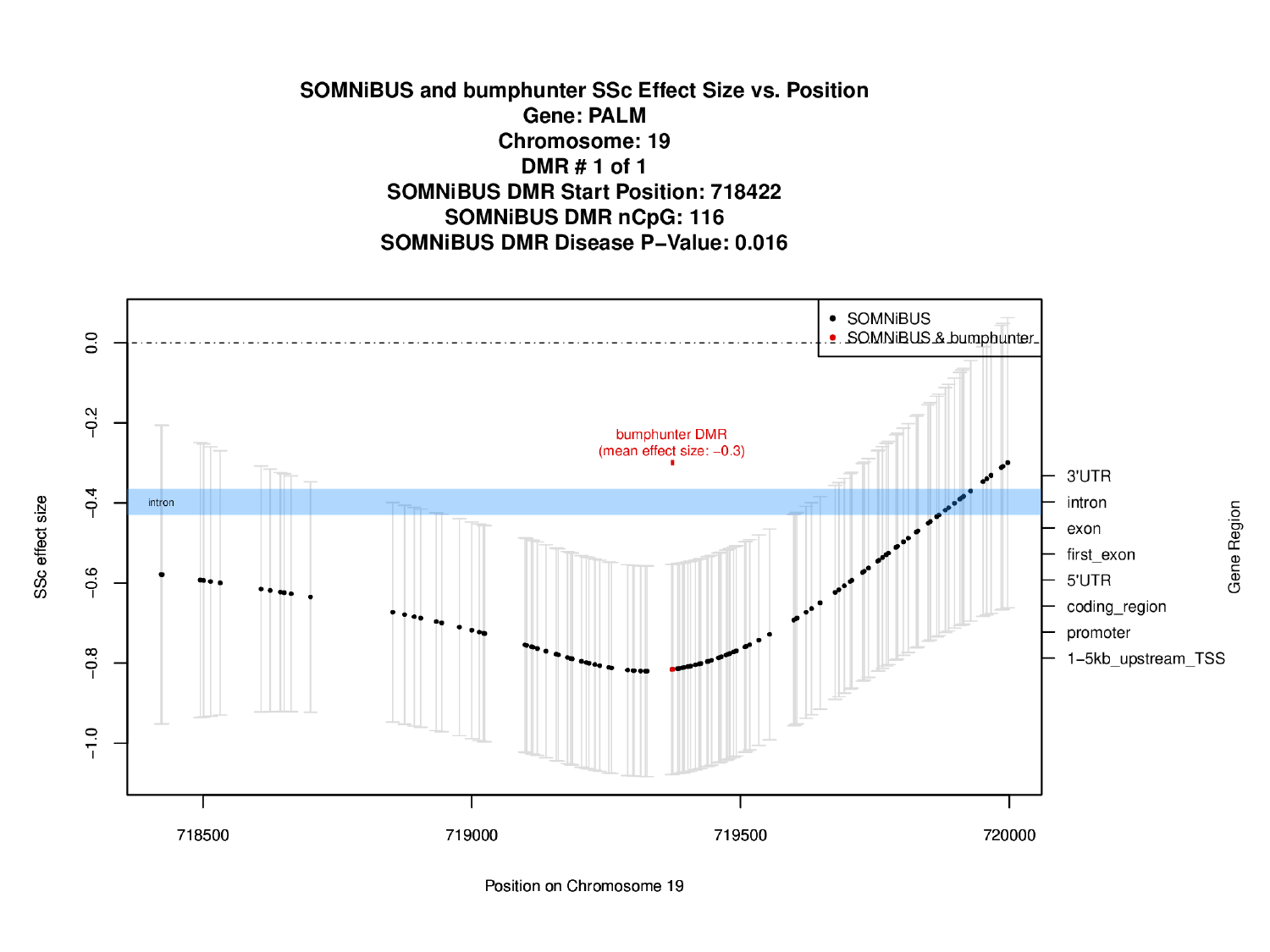

## Slide 30
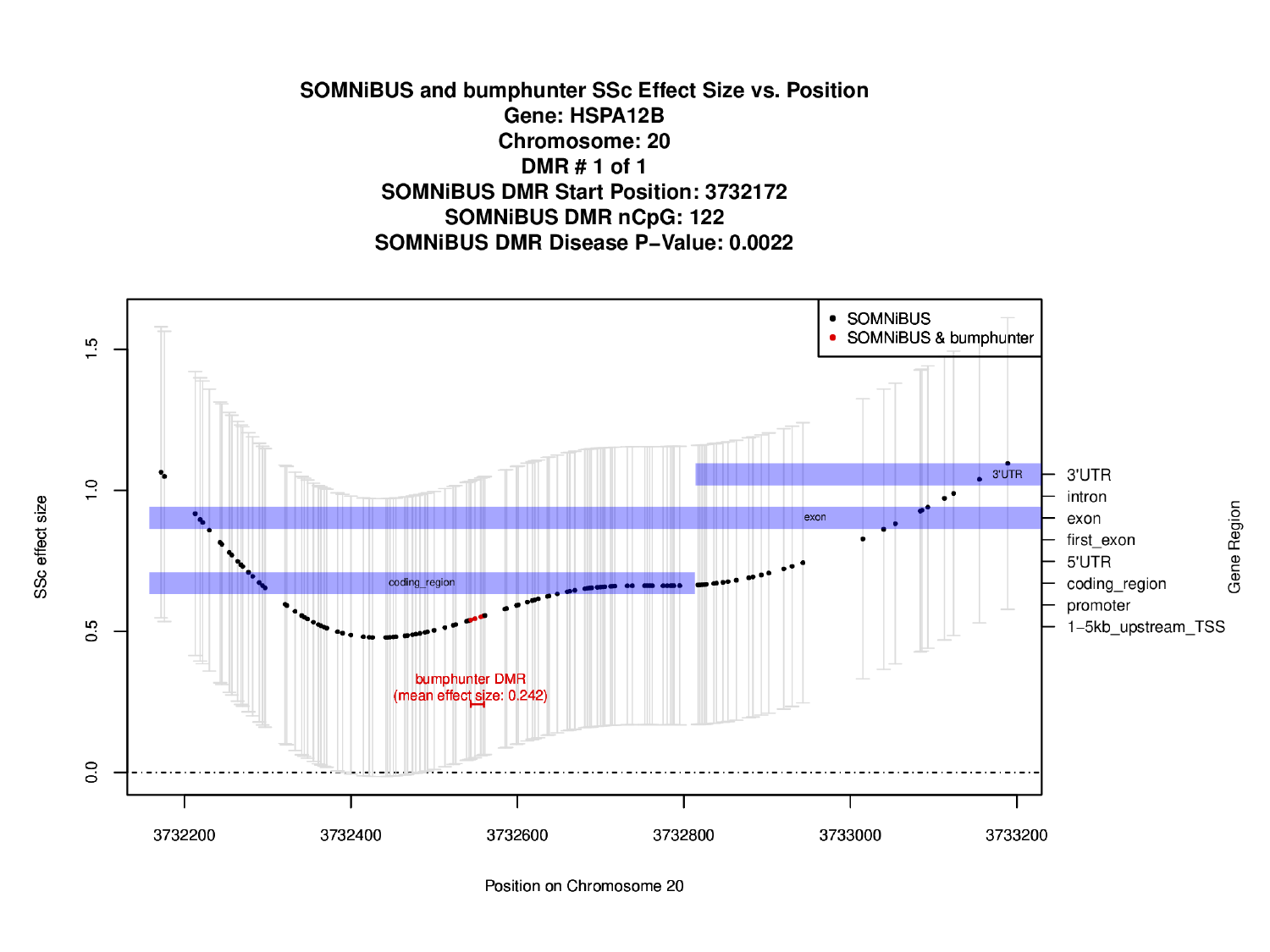

## Slide 31
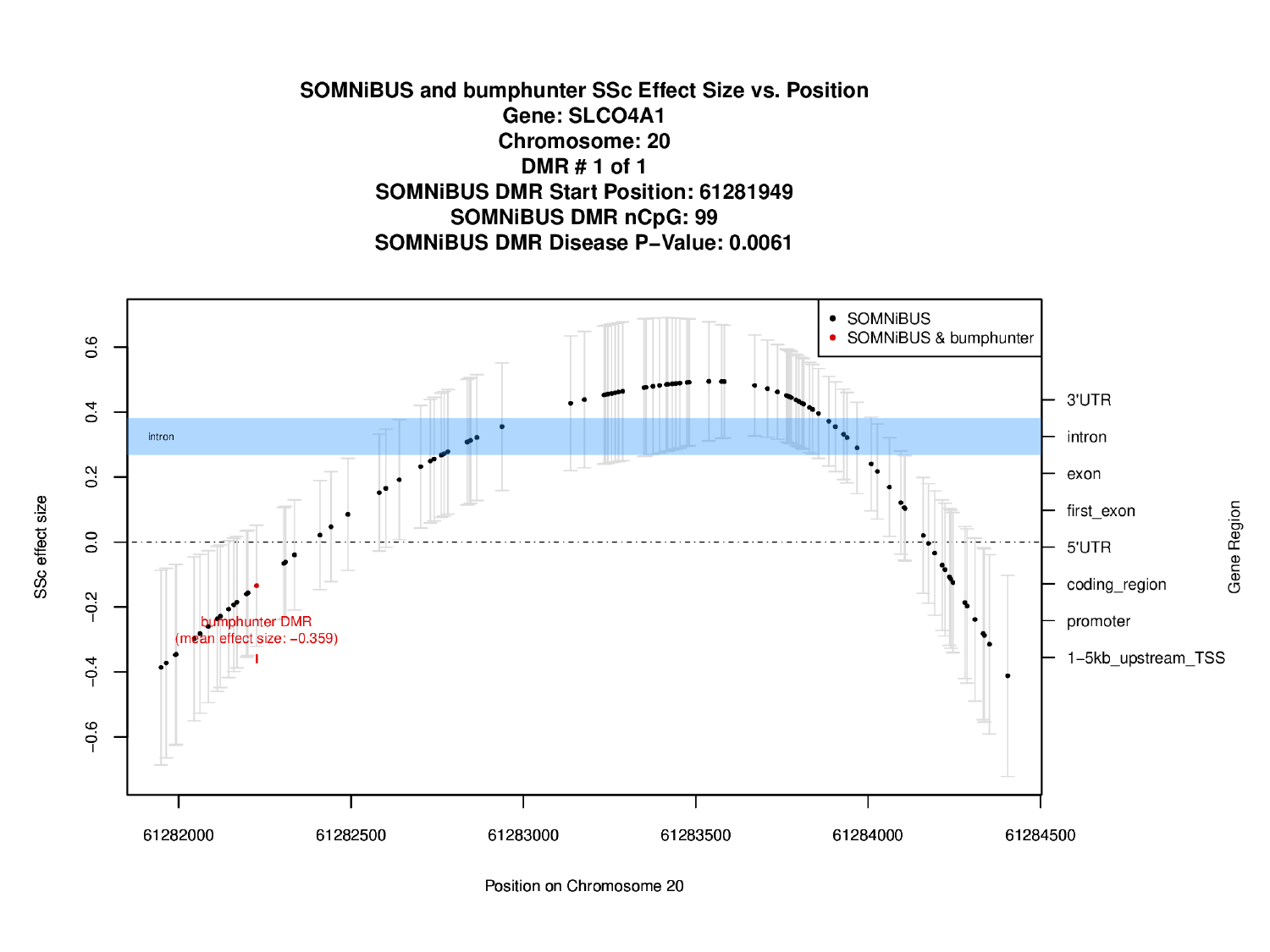

## Slide 32
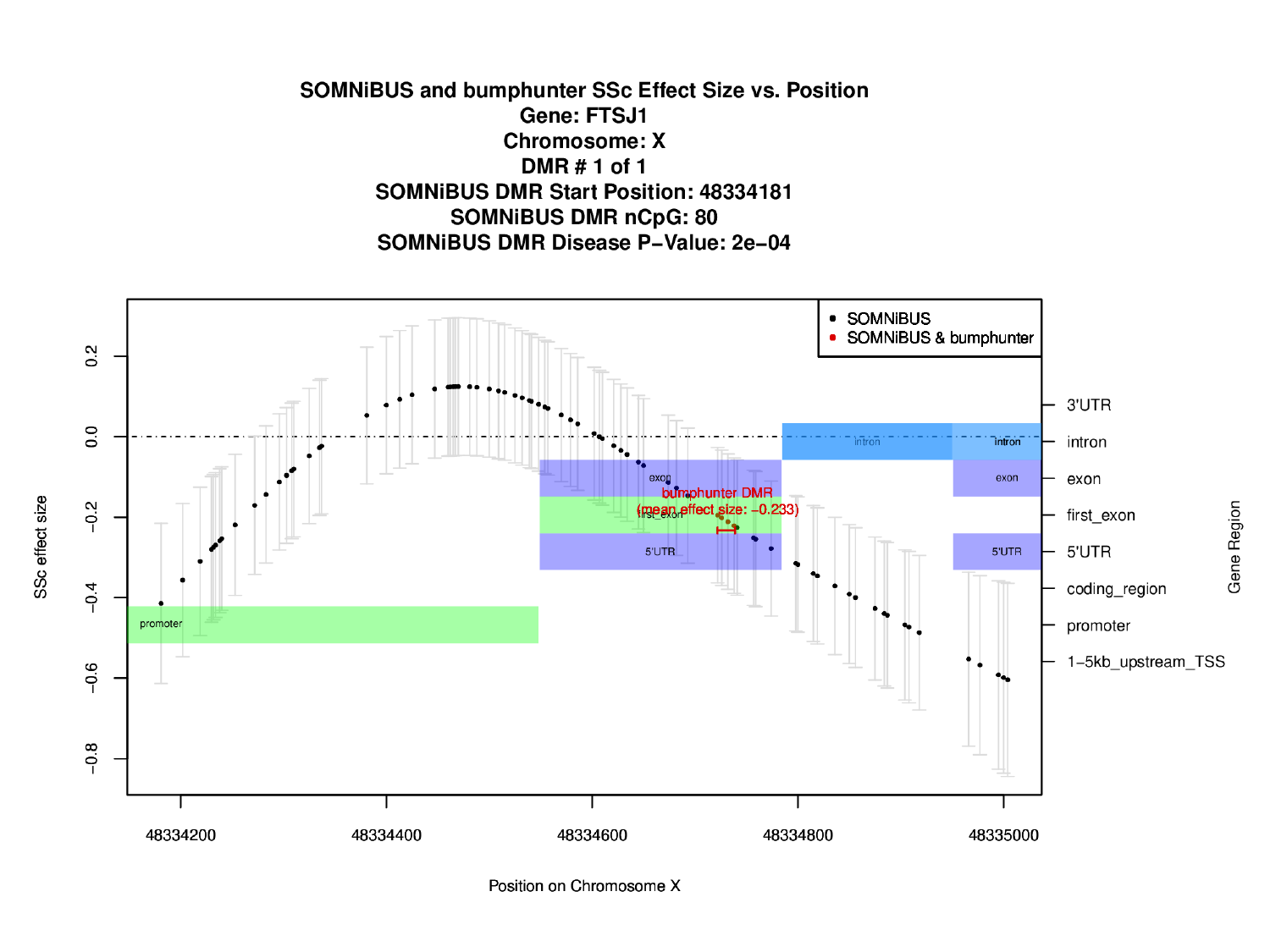

## Slide 33
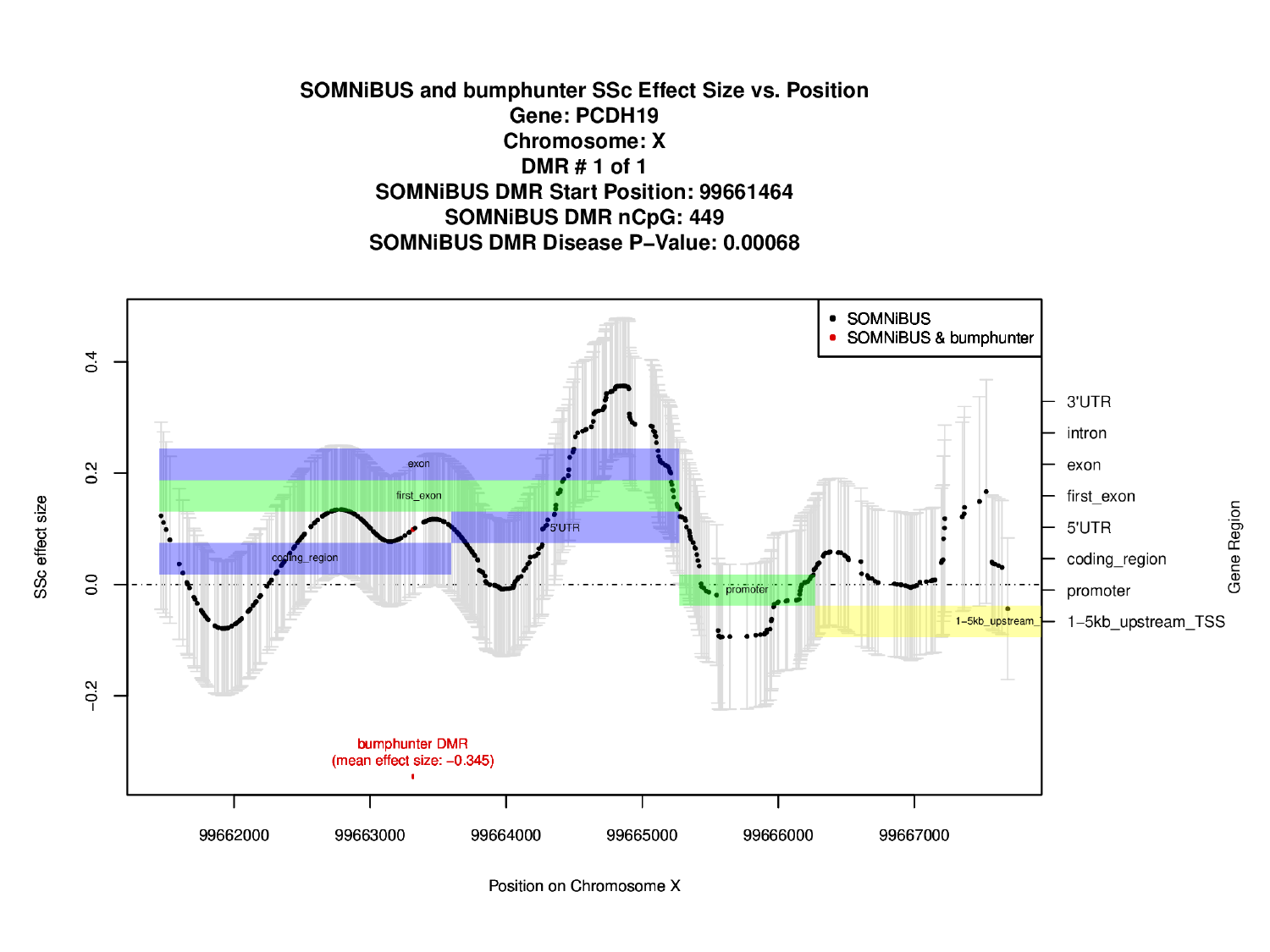

## Slide 34
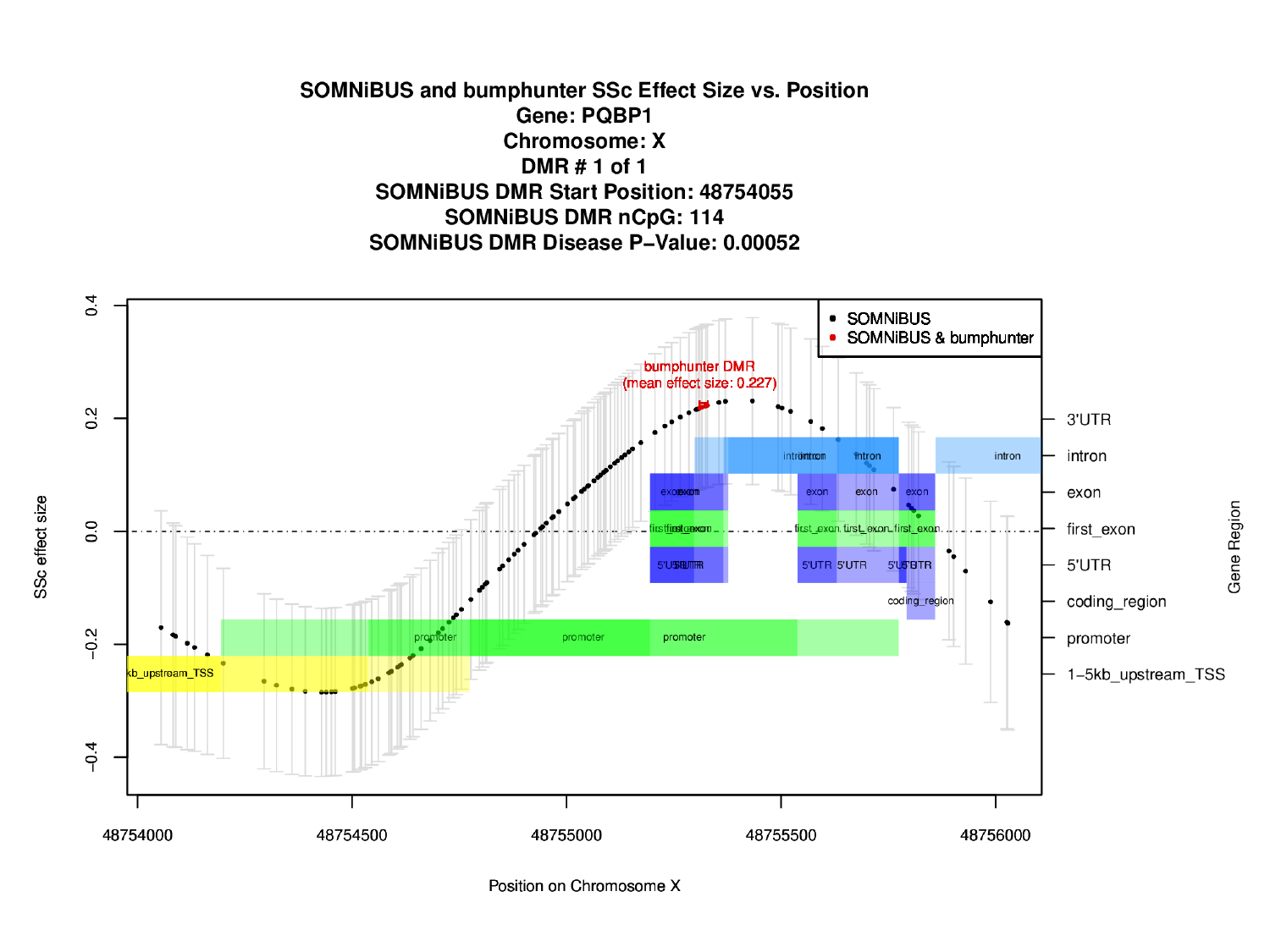

## Slide 35
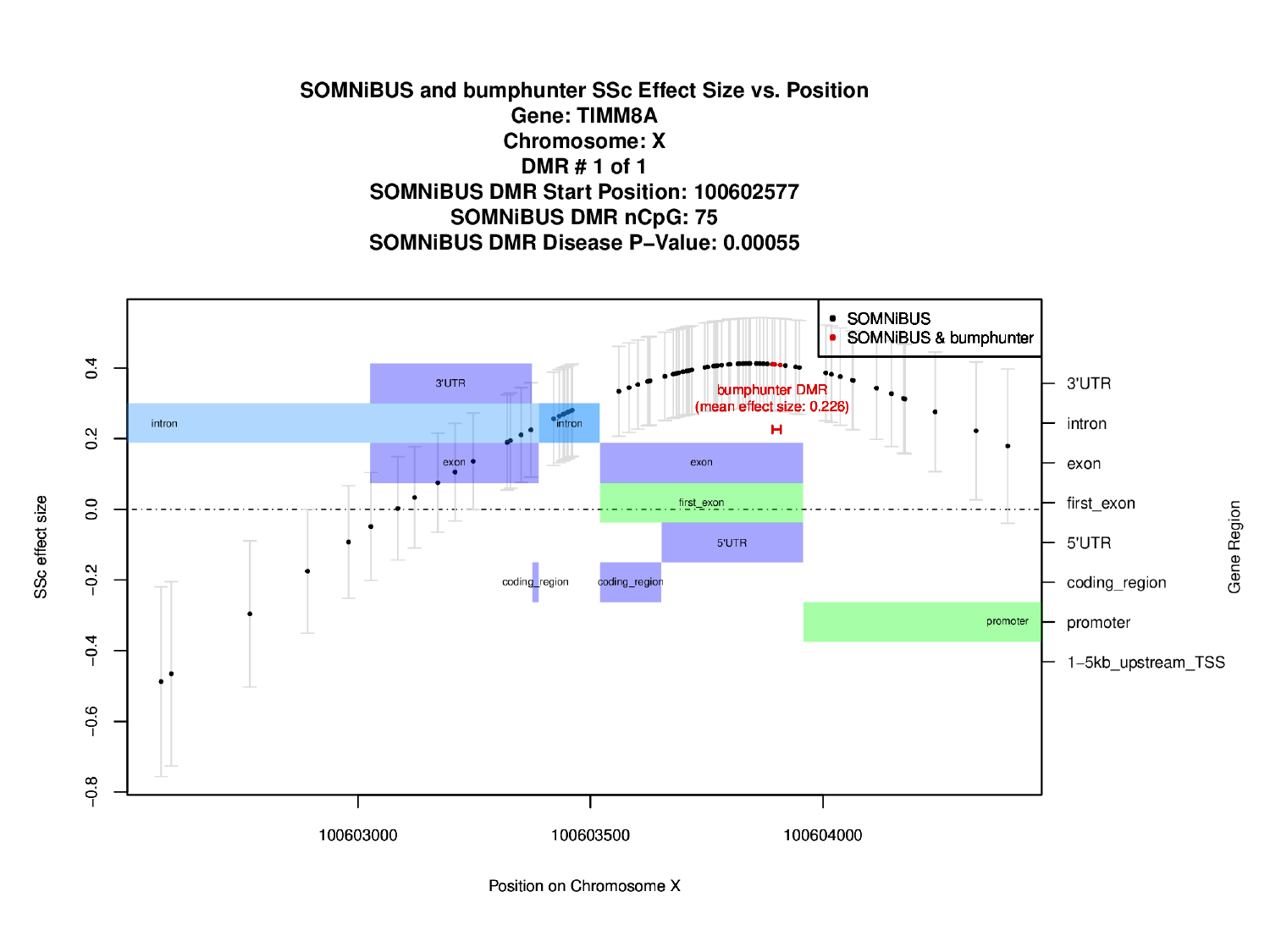

Supplement: Supplementary file 3 — Additional file 3: Nucleotide-level smoothed regional disease effect coefficients for all CpG regions identified by SOMNiBUS with p-value < 0.05 and identified by bumphunter with q-value < 0.05 [file 13148_2023_1513_MOESM3_ESM.pptx]
