# Supplementary material for: Novel insights into systemic sclerosis using a sensitive computational method to analyze whole-genome bisulfite sequencing data
Source: Clin Epigenetics. 2023 Jun 3;15:96. doi: 10.1186/s13148-023-01513-w (PMC10239181; doi:10.1186/s13148-023-01513-w)

## Slide 1
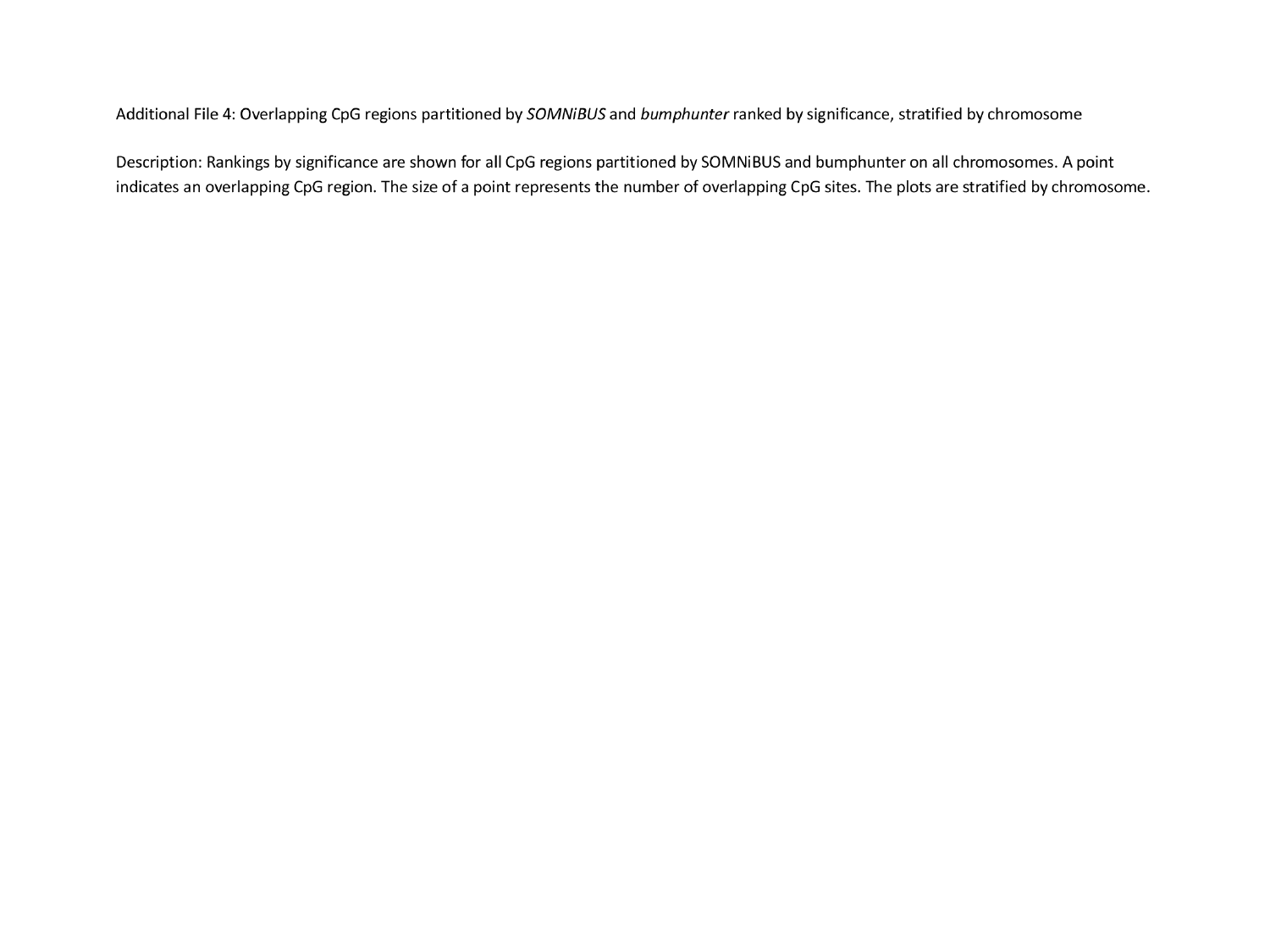

## Slide 2
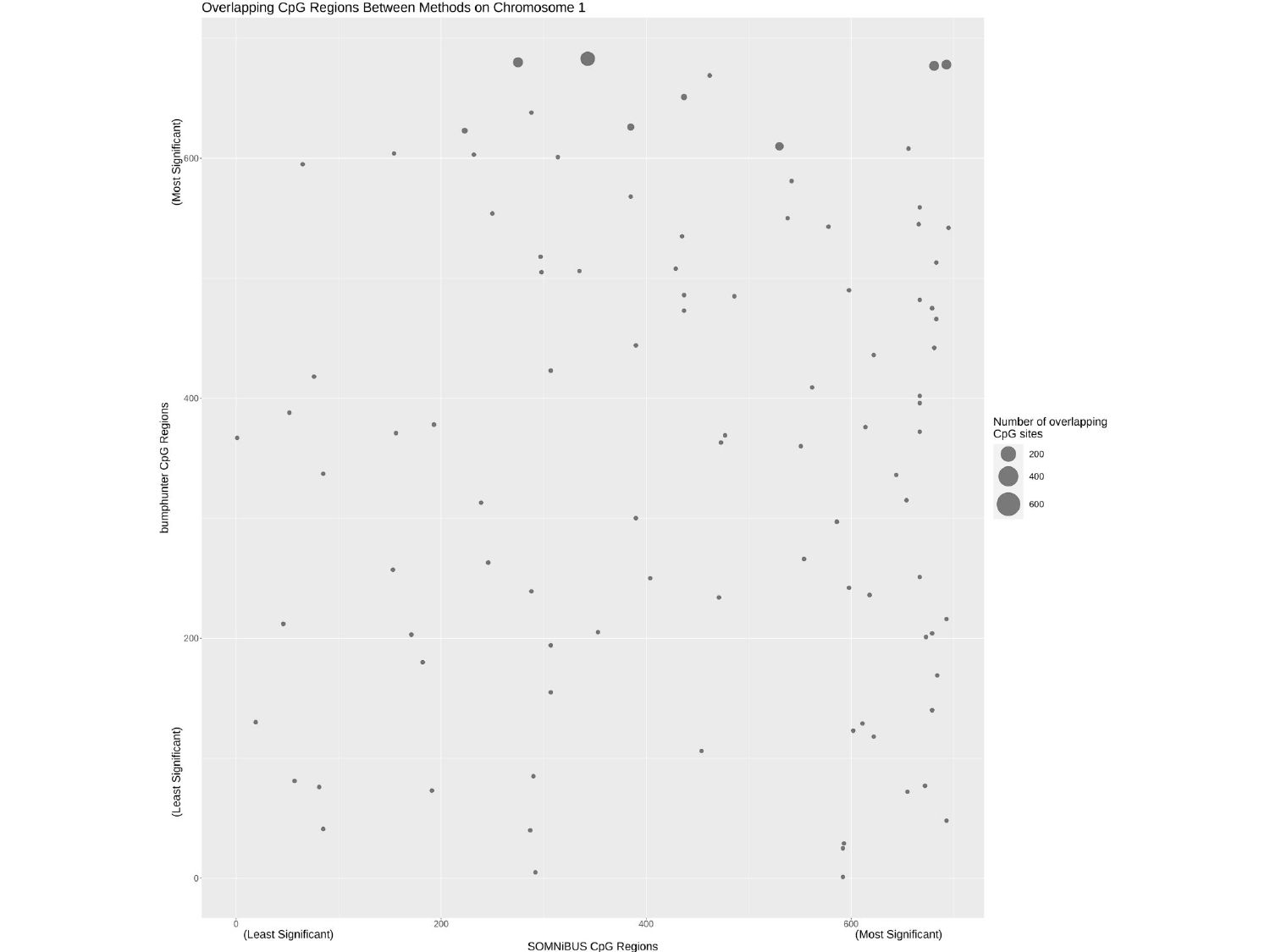

## Slide 3
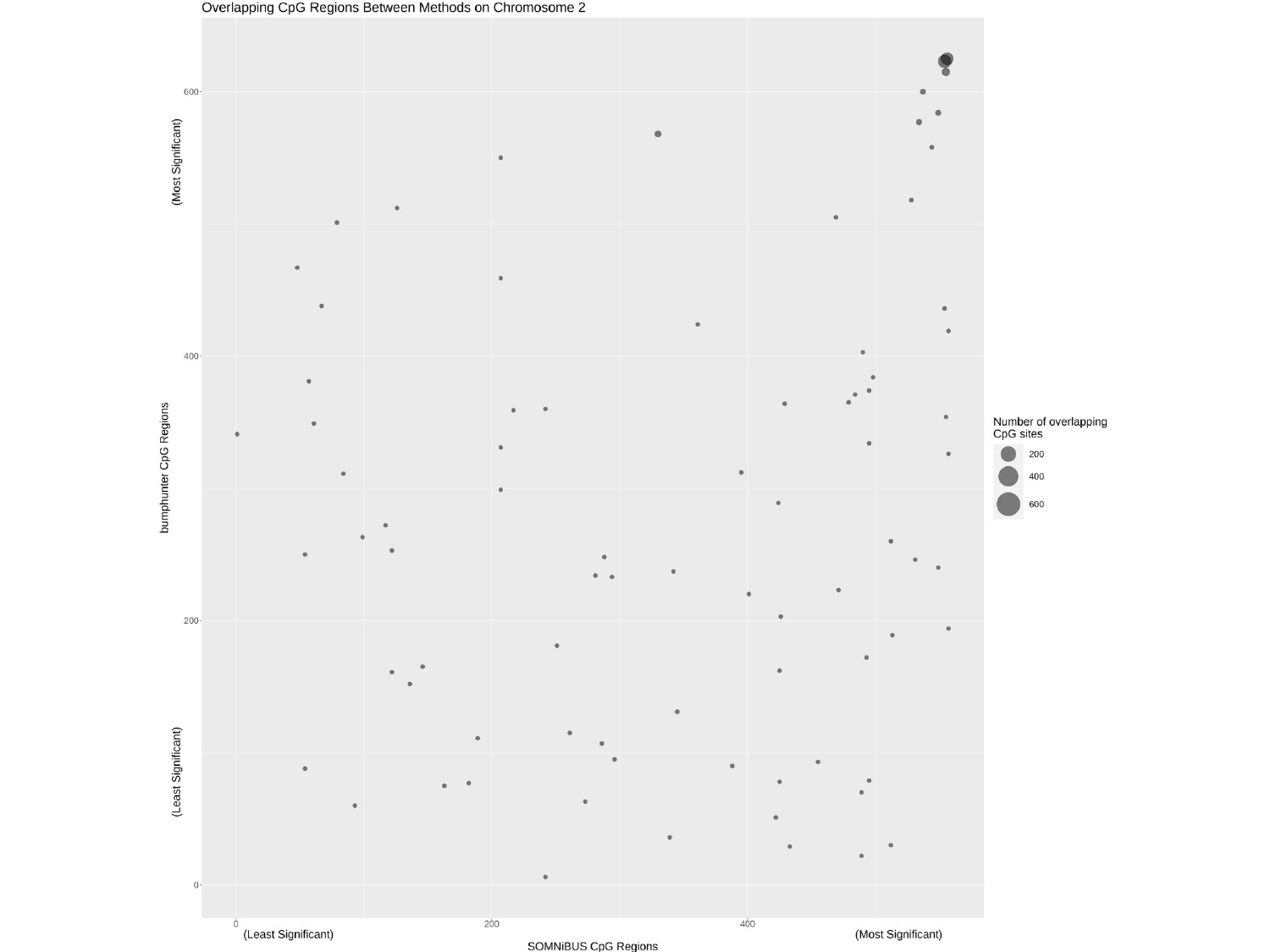

## Slide 4
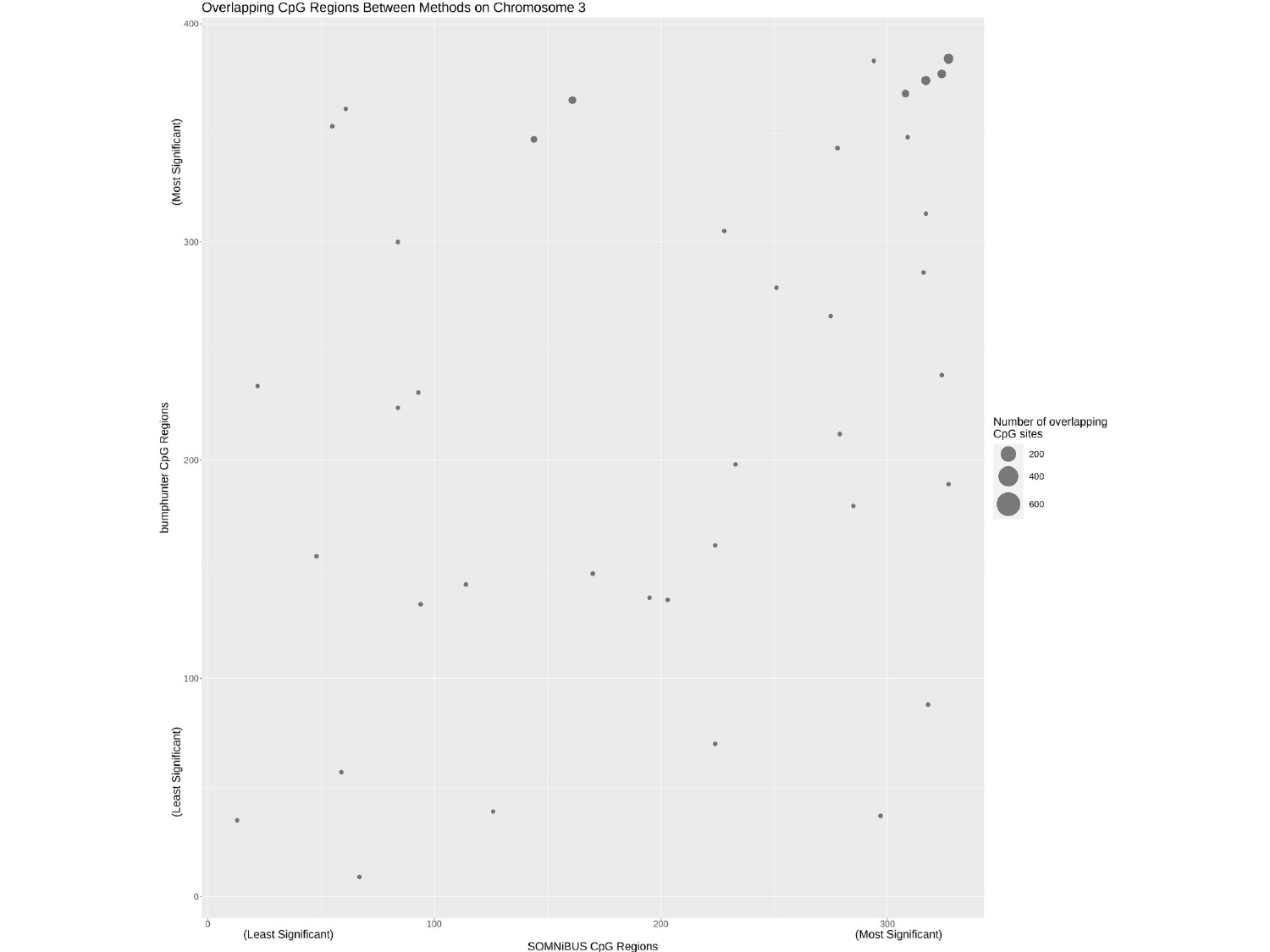

## Slide 5
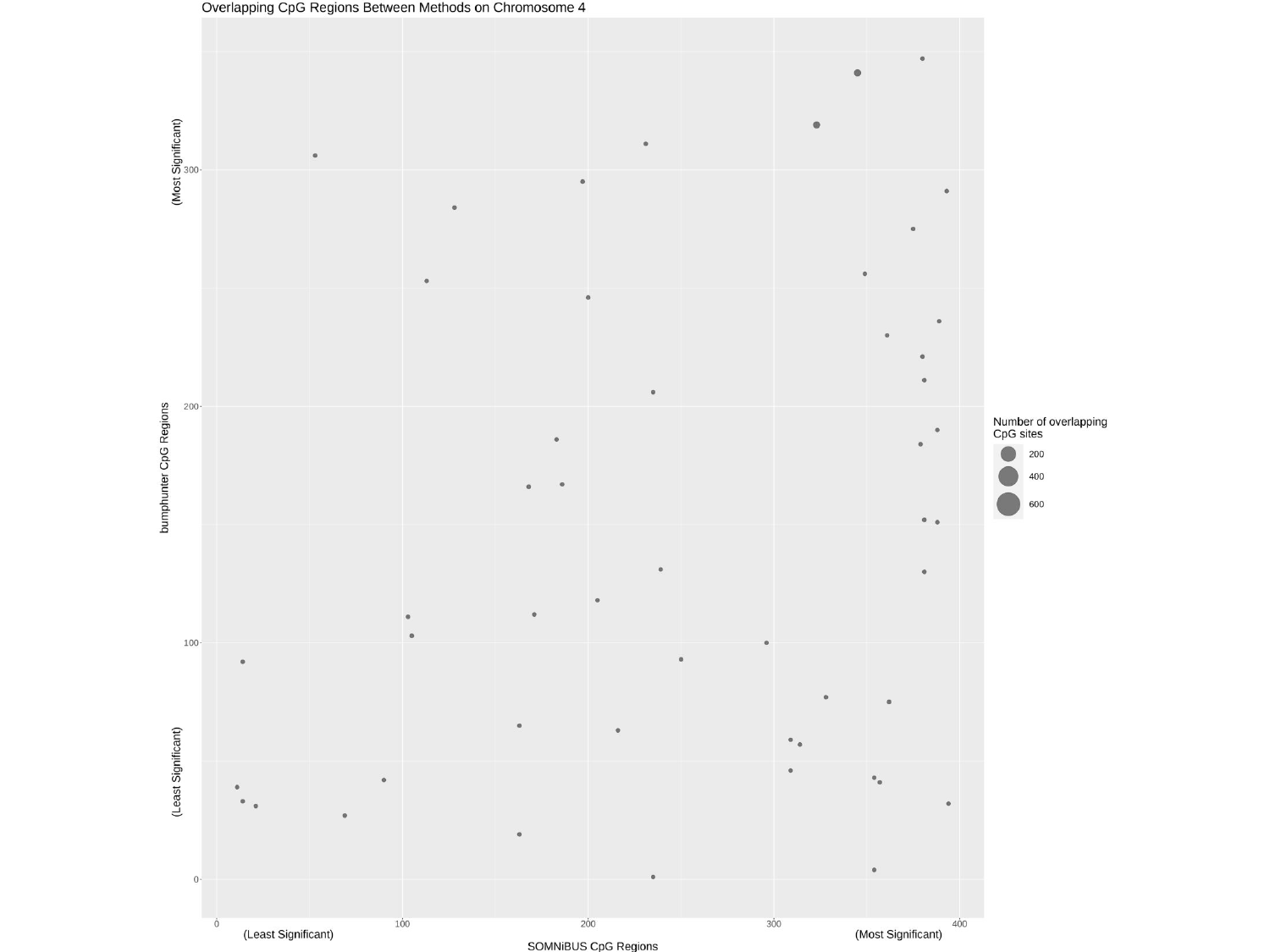

## Slide 6
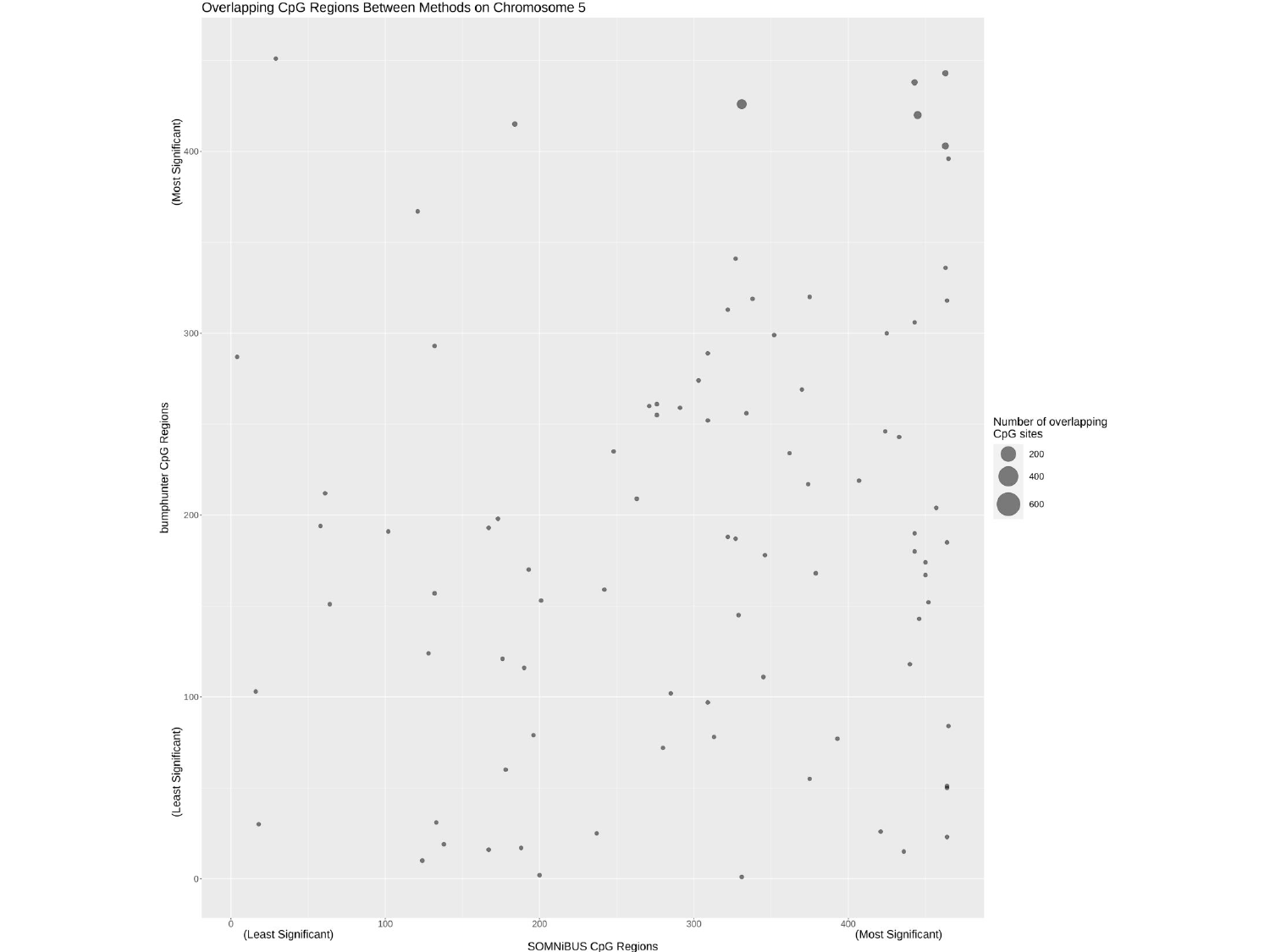

## Slide 7
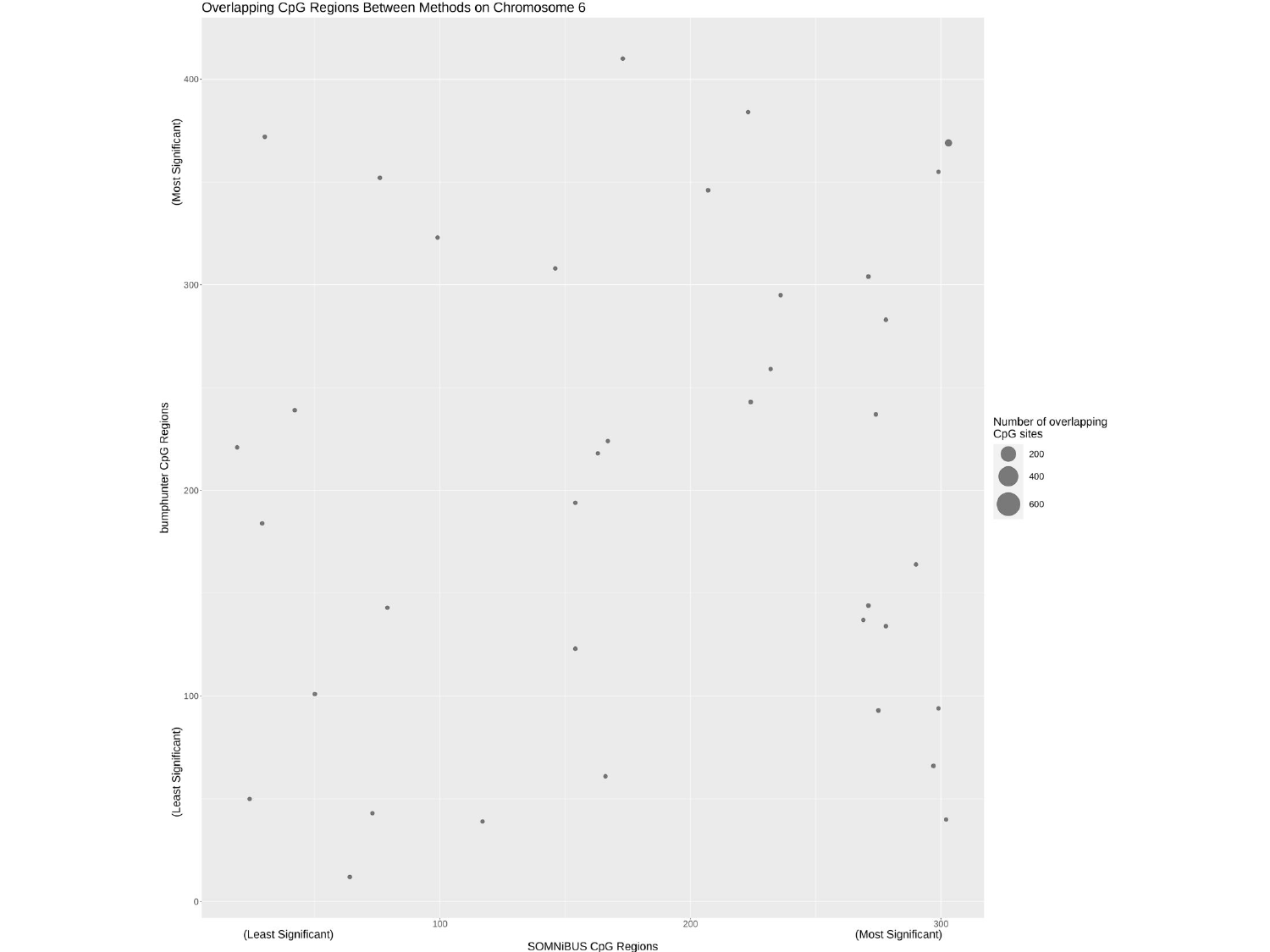

## Slide 8
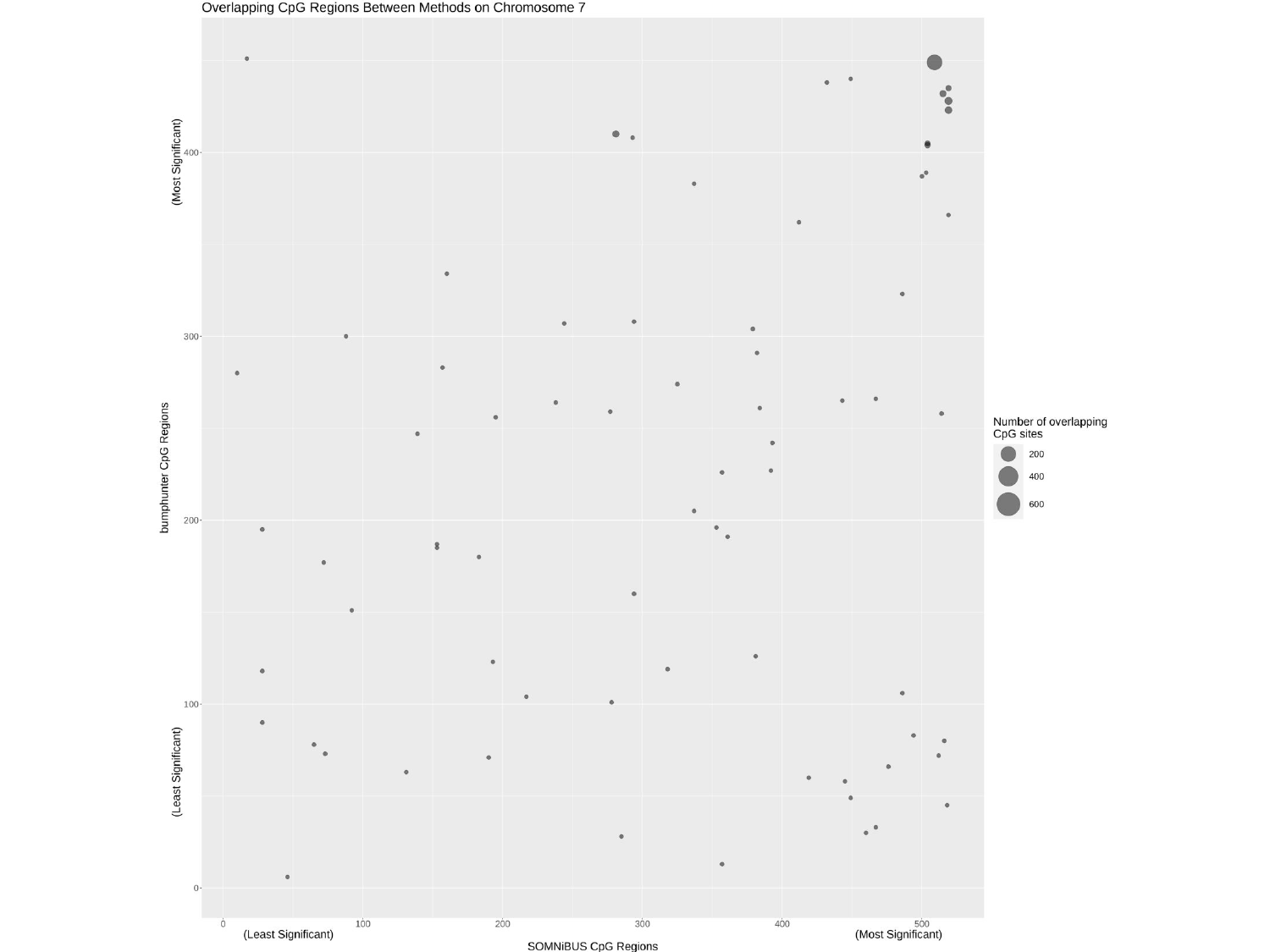

## Slide 9
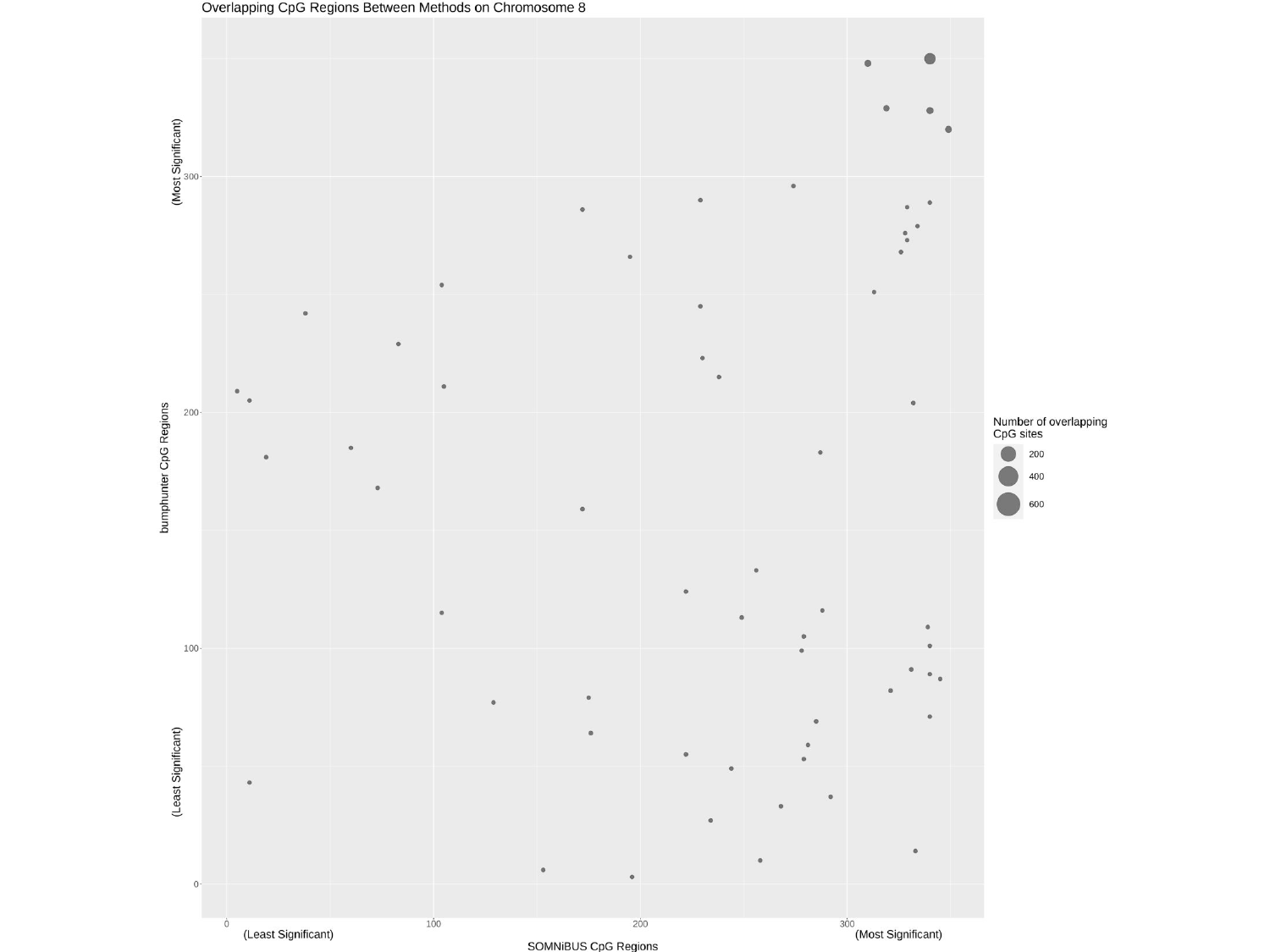

## Slide 10
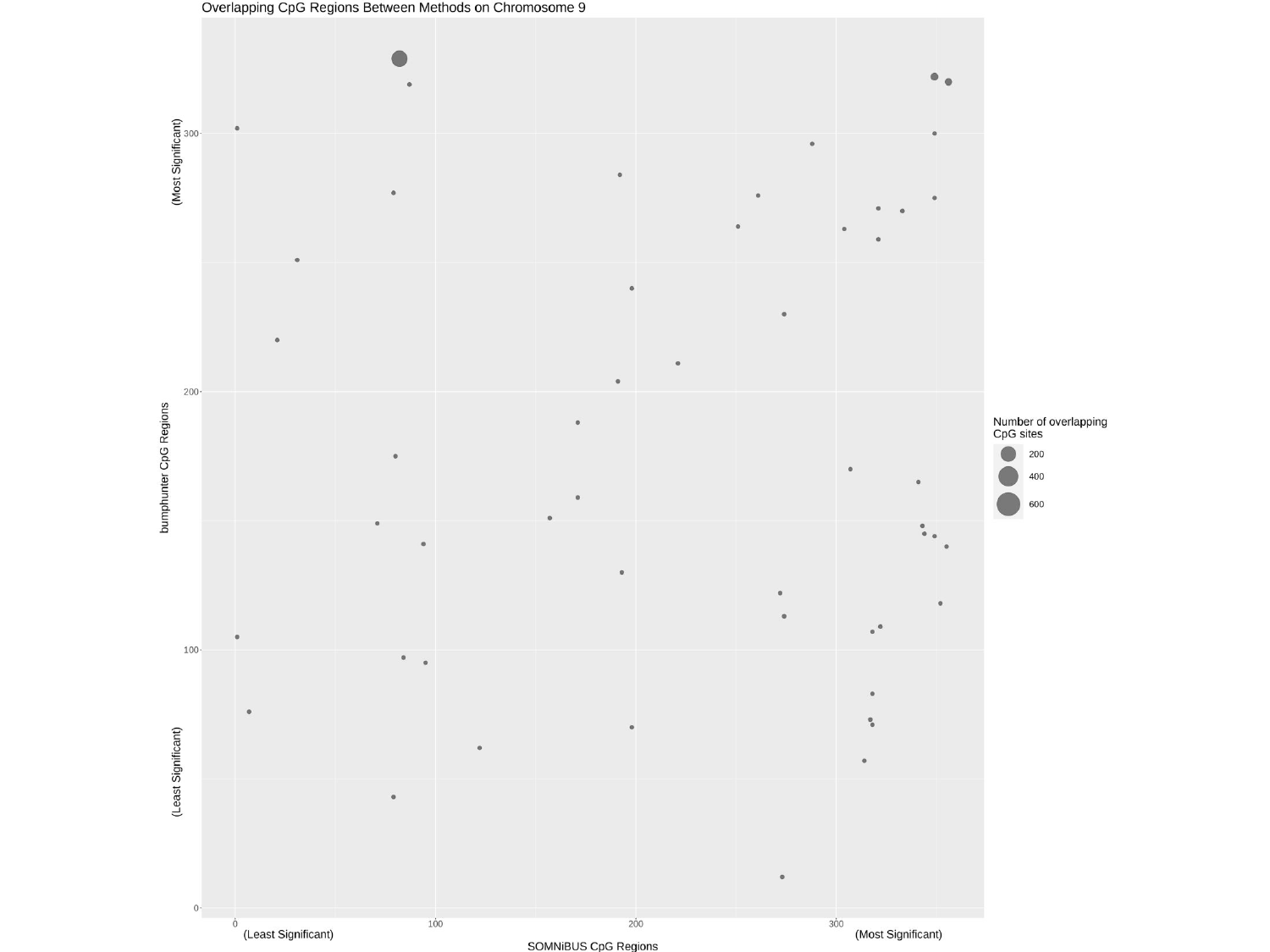

## Slide 11
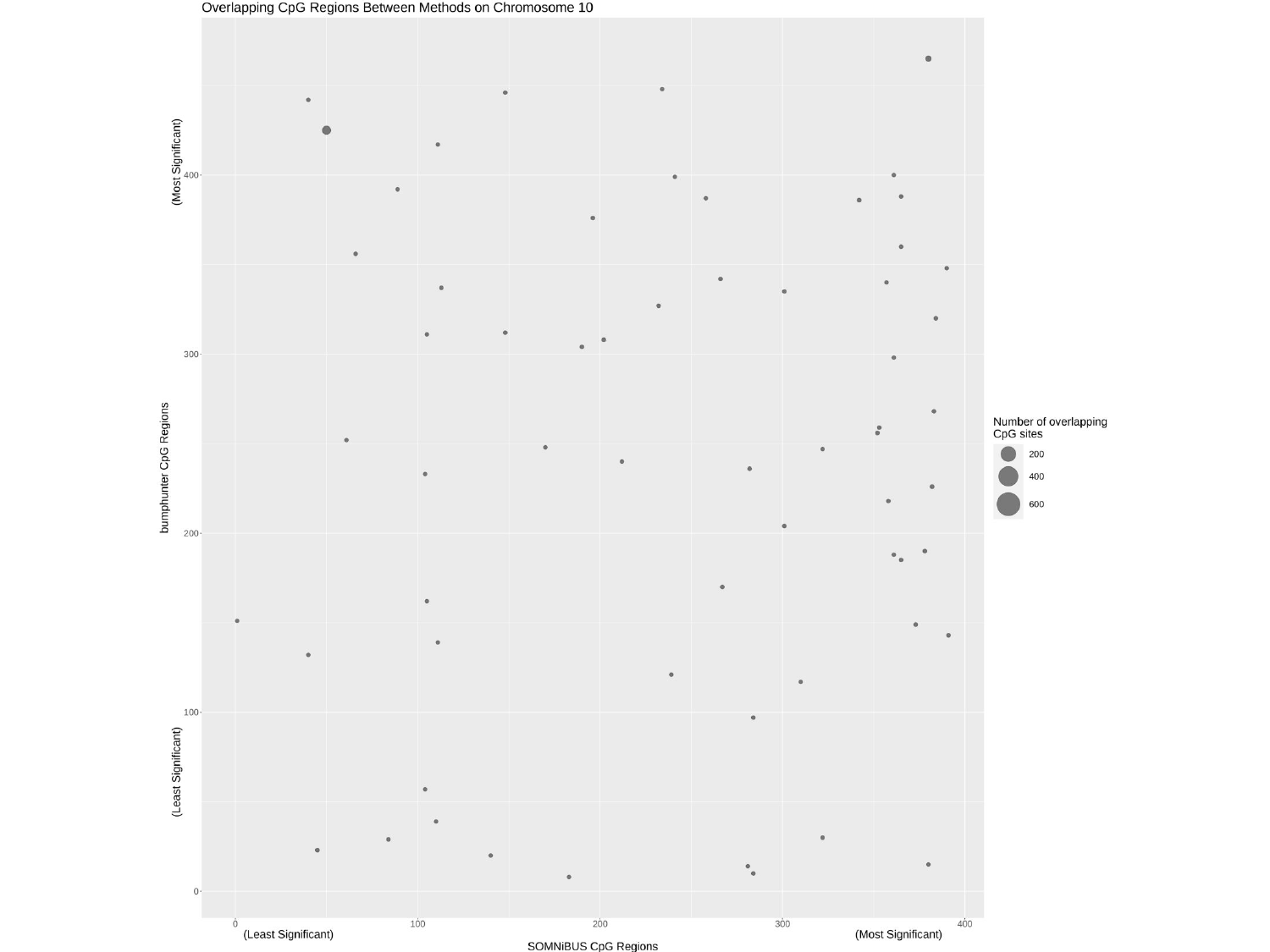

## Slide 12
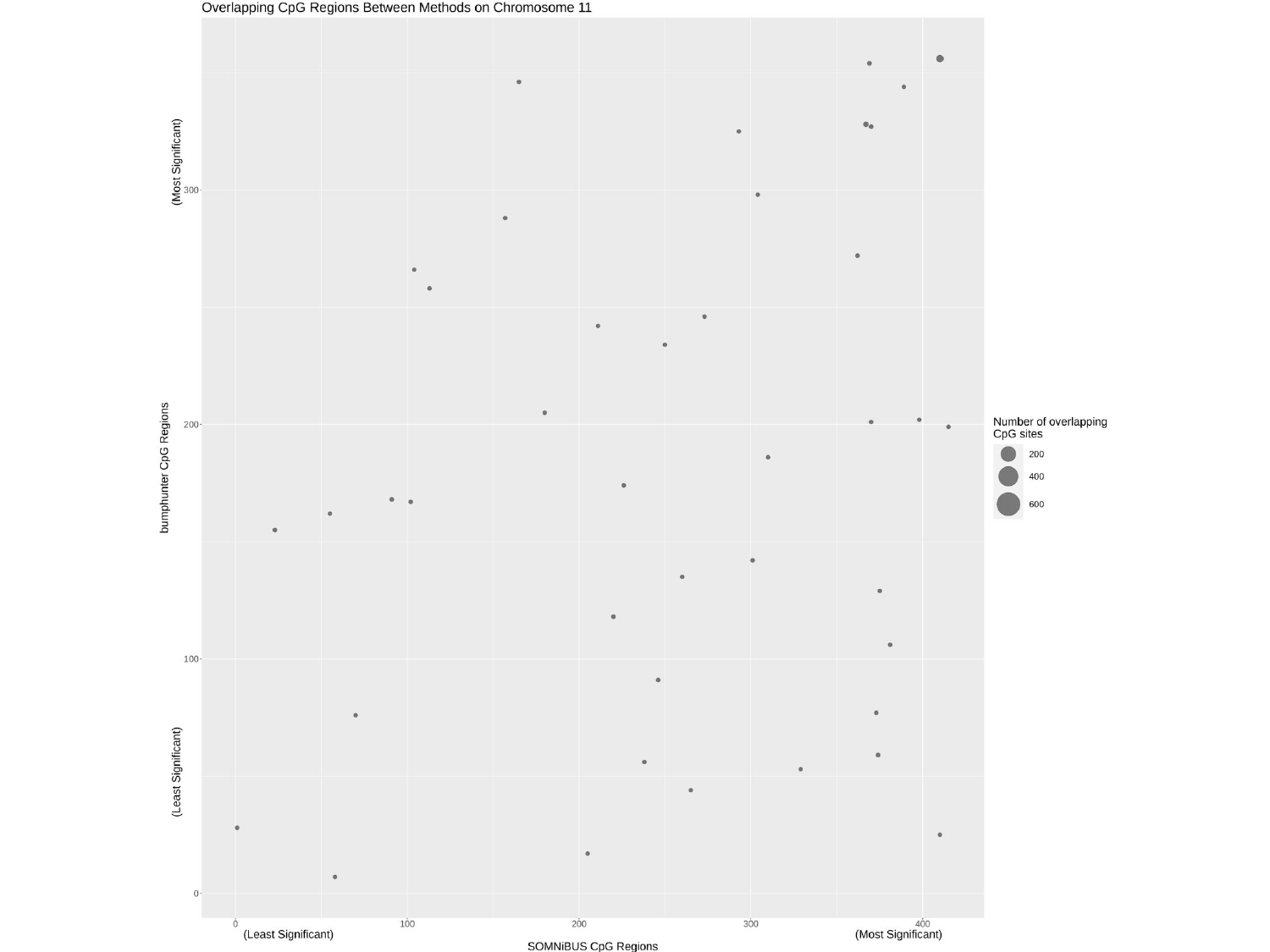

## Slide 13
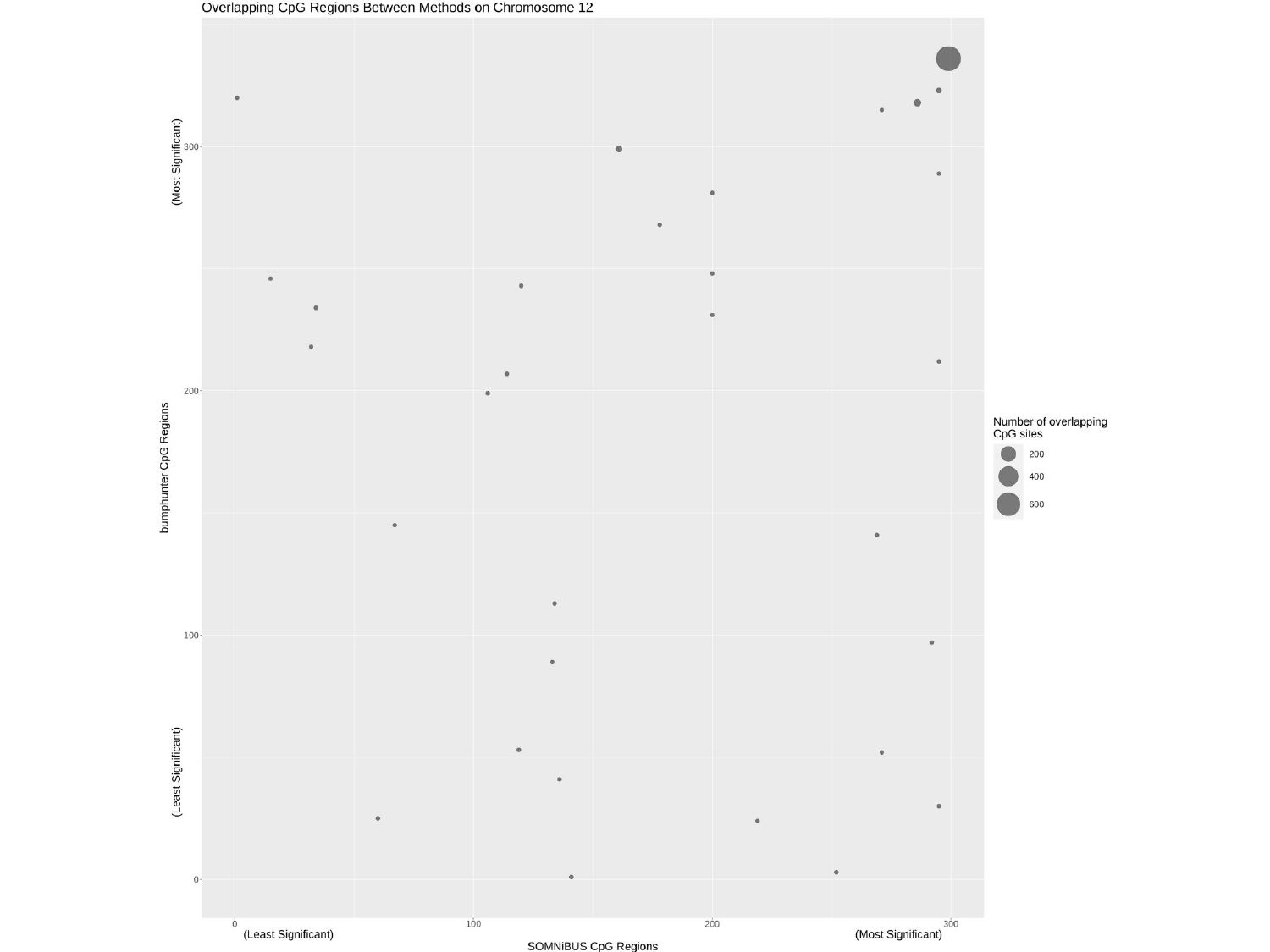

## Slide 14
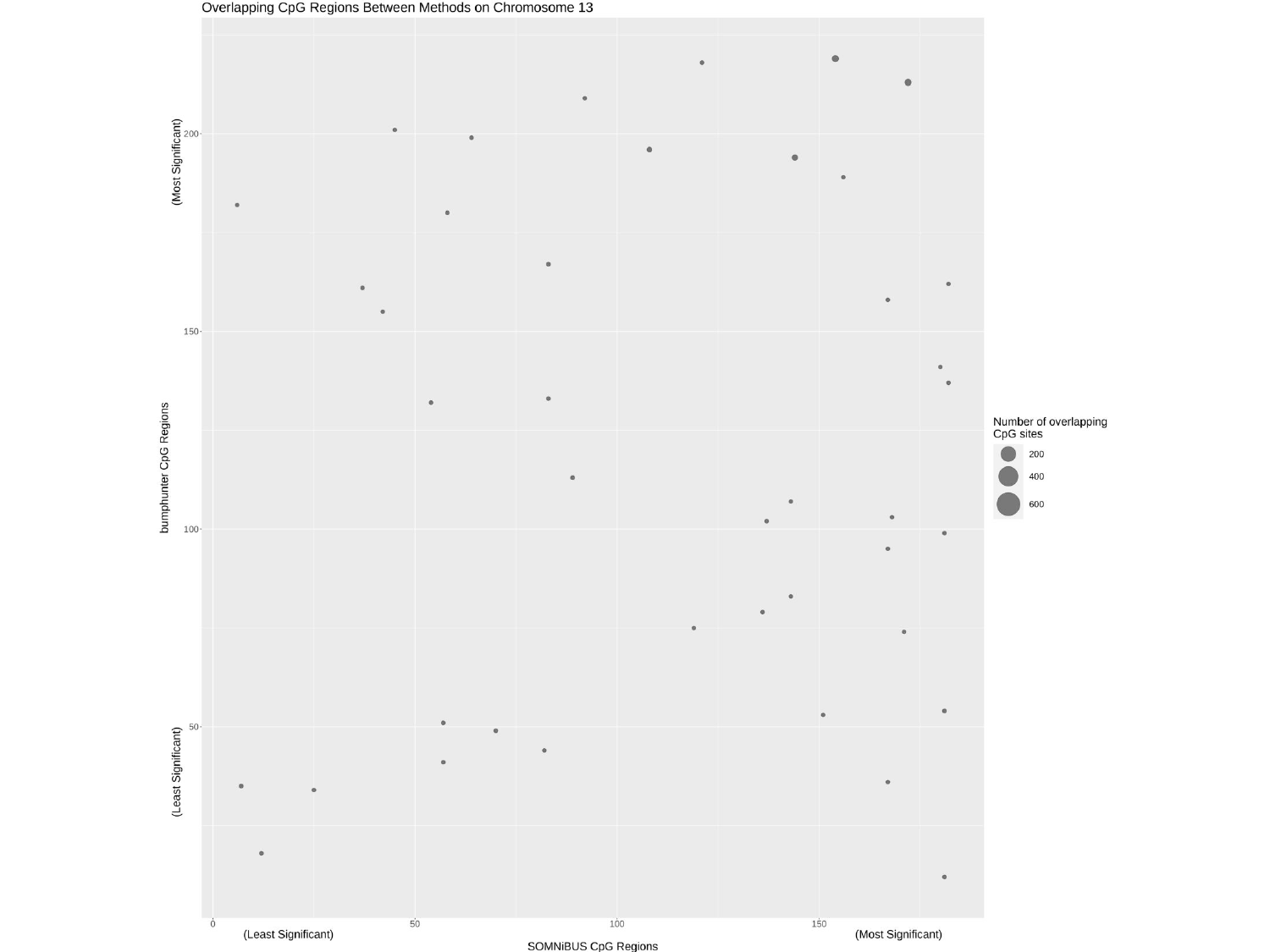

## Slide 15
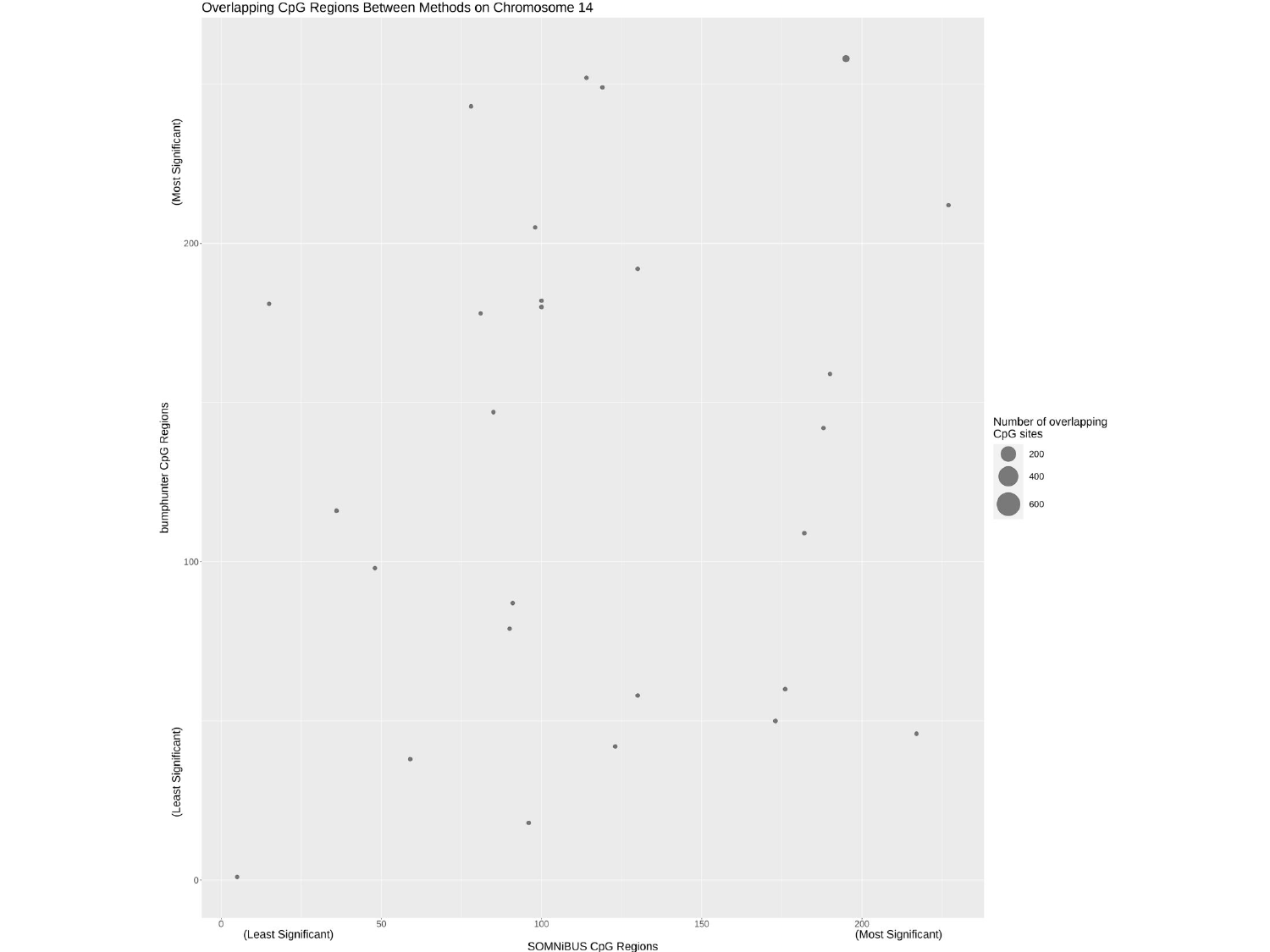

## Slide 16
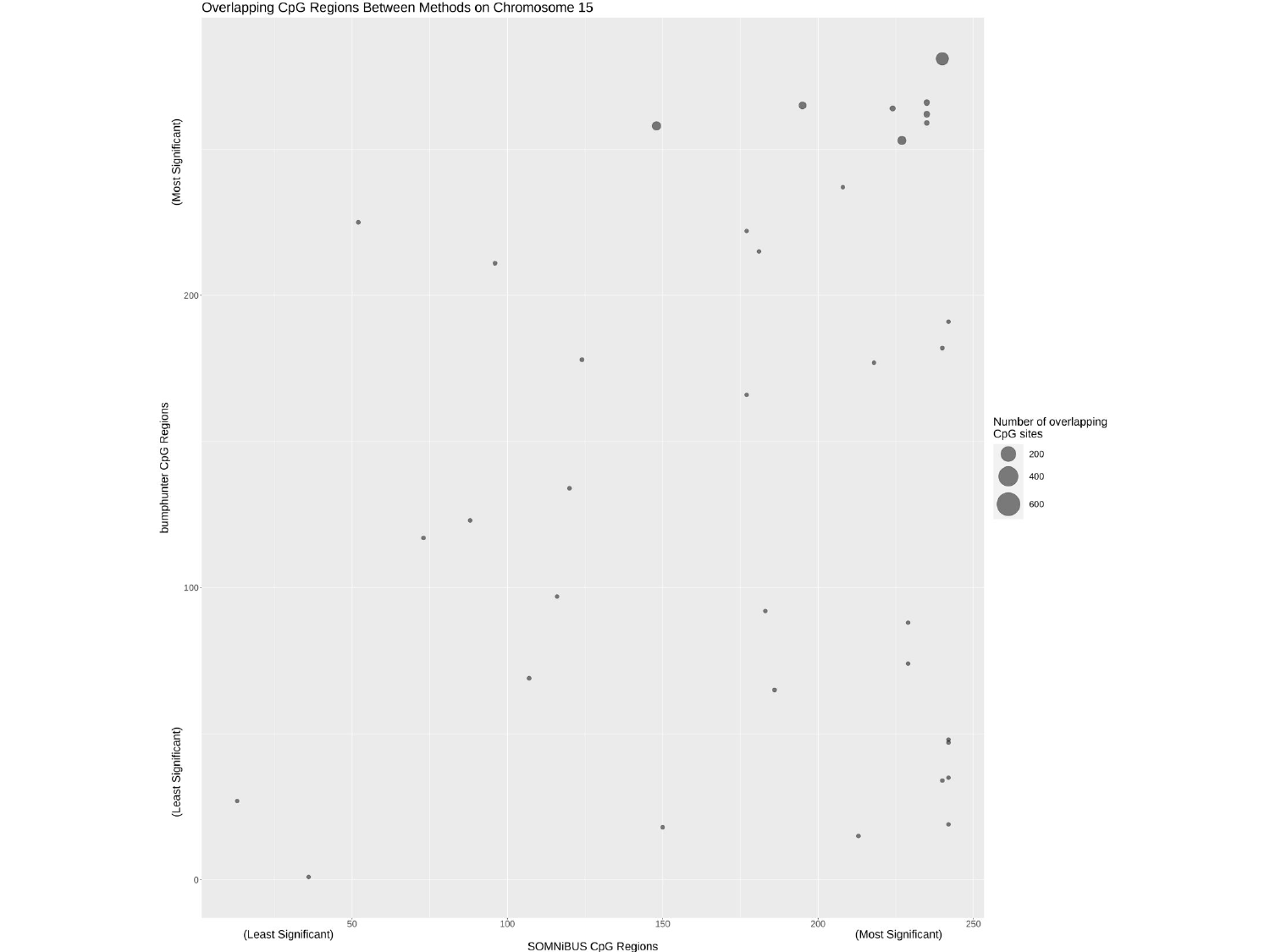

## Slide 17
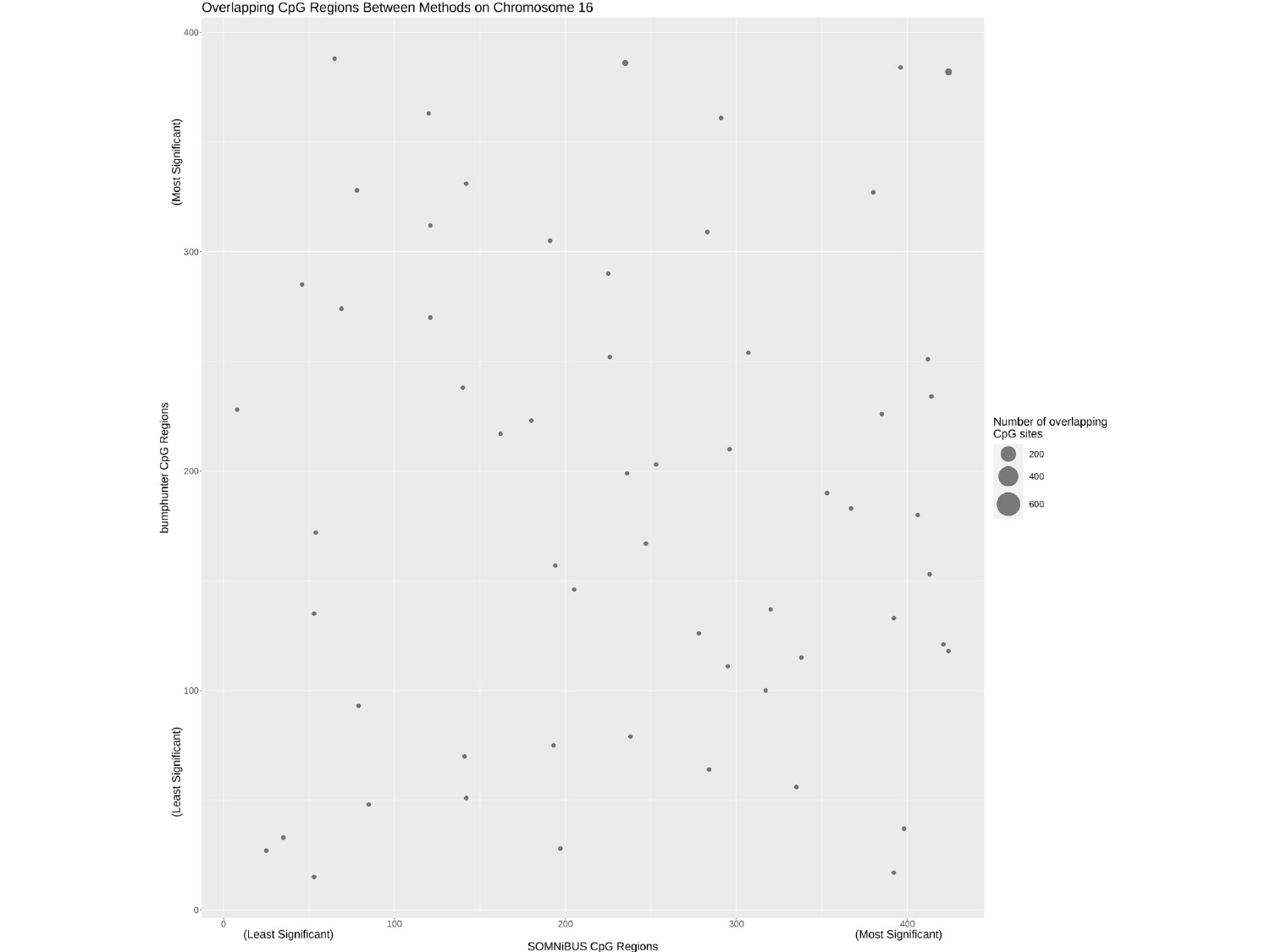

## Slide 18
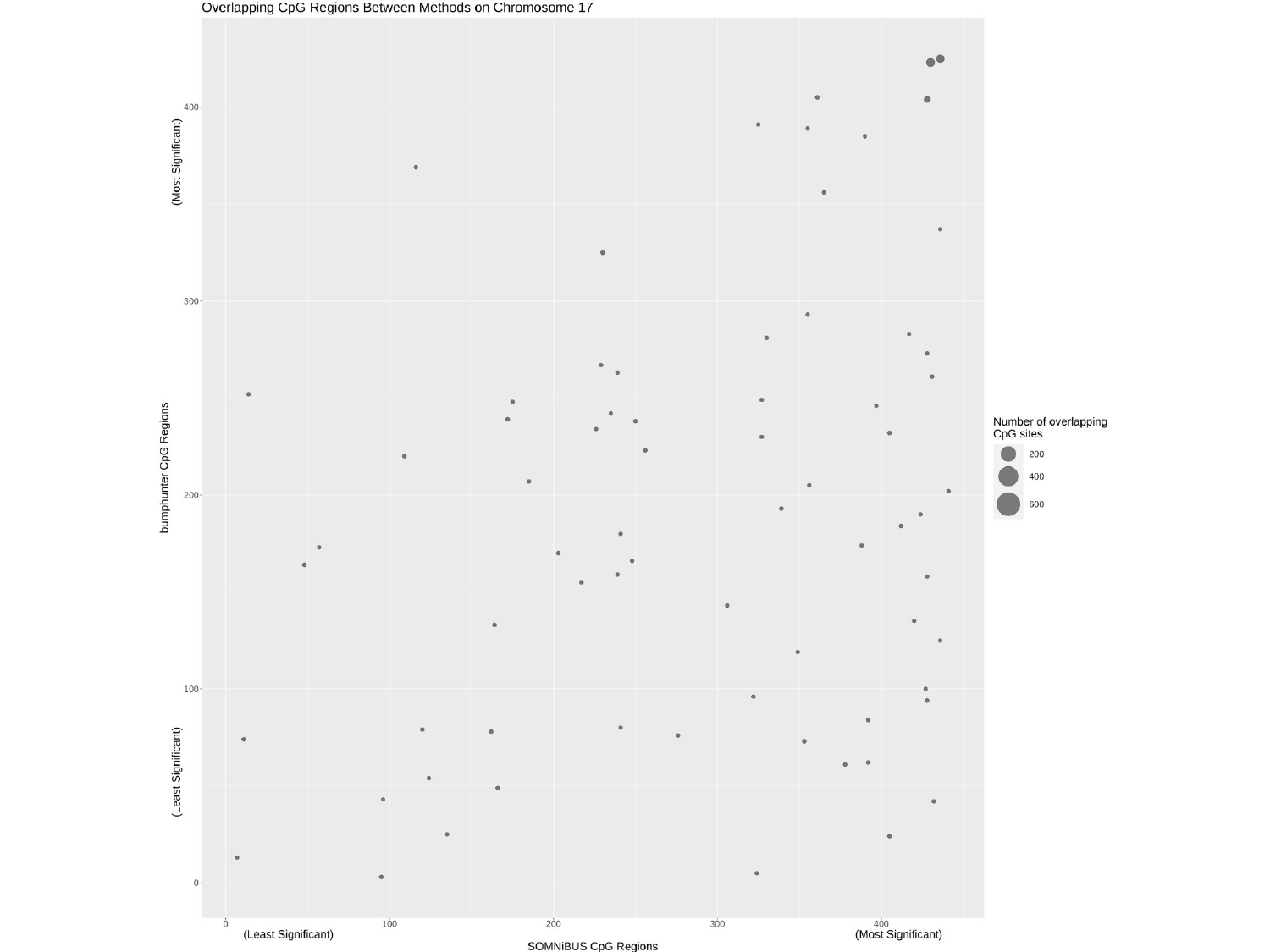

## Slide 19
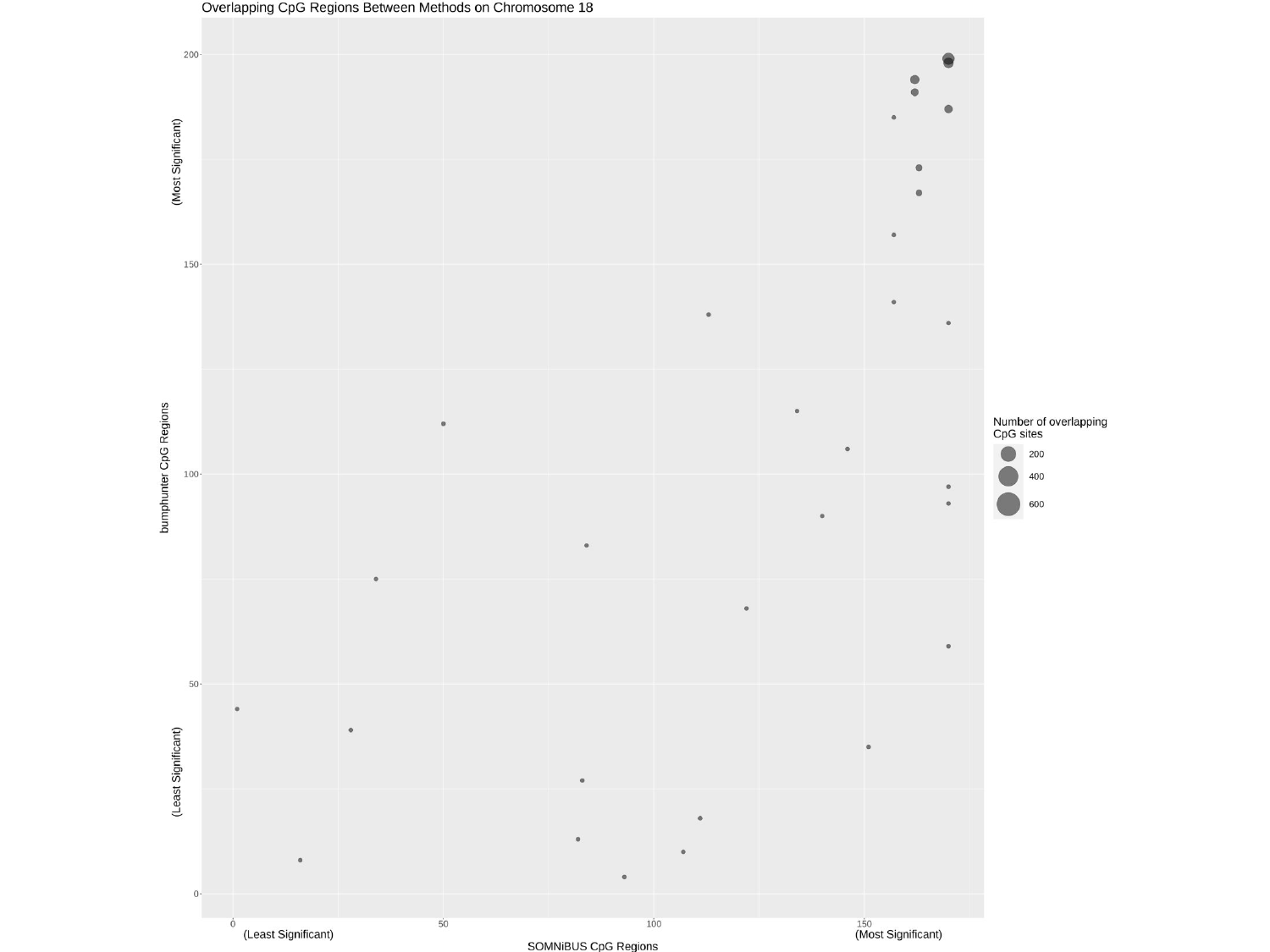

## Slide 20
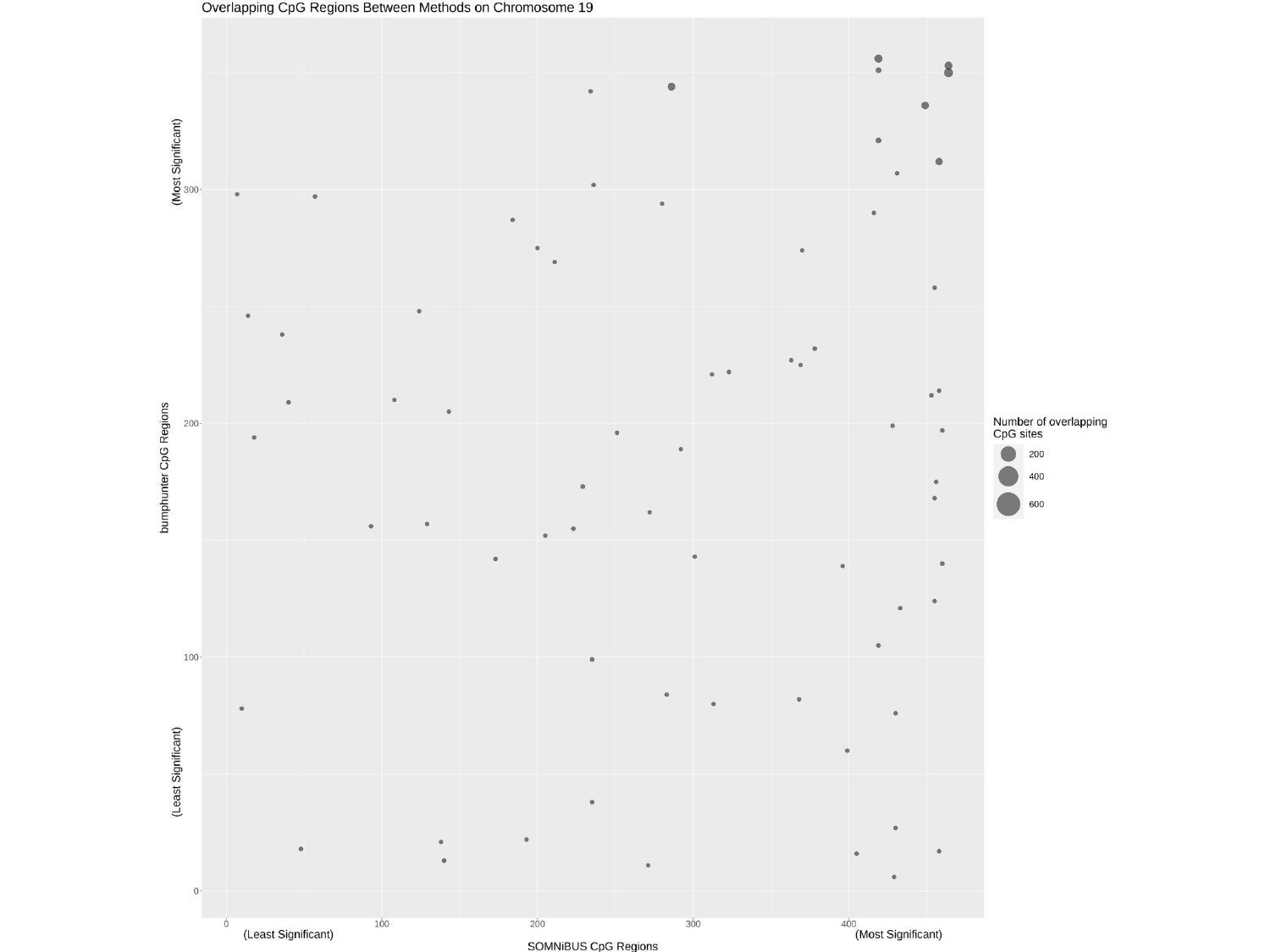

## Slide 21
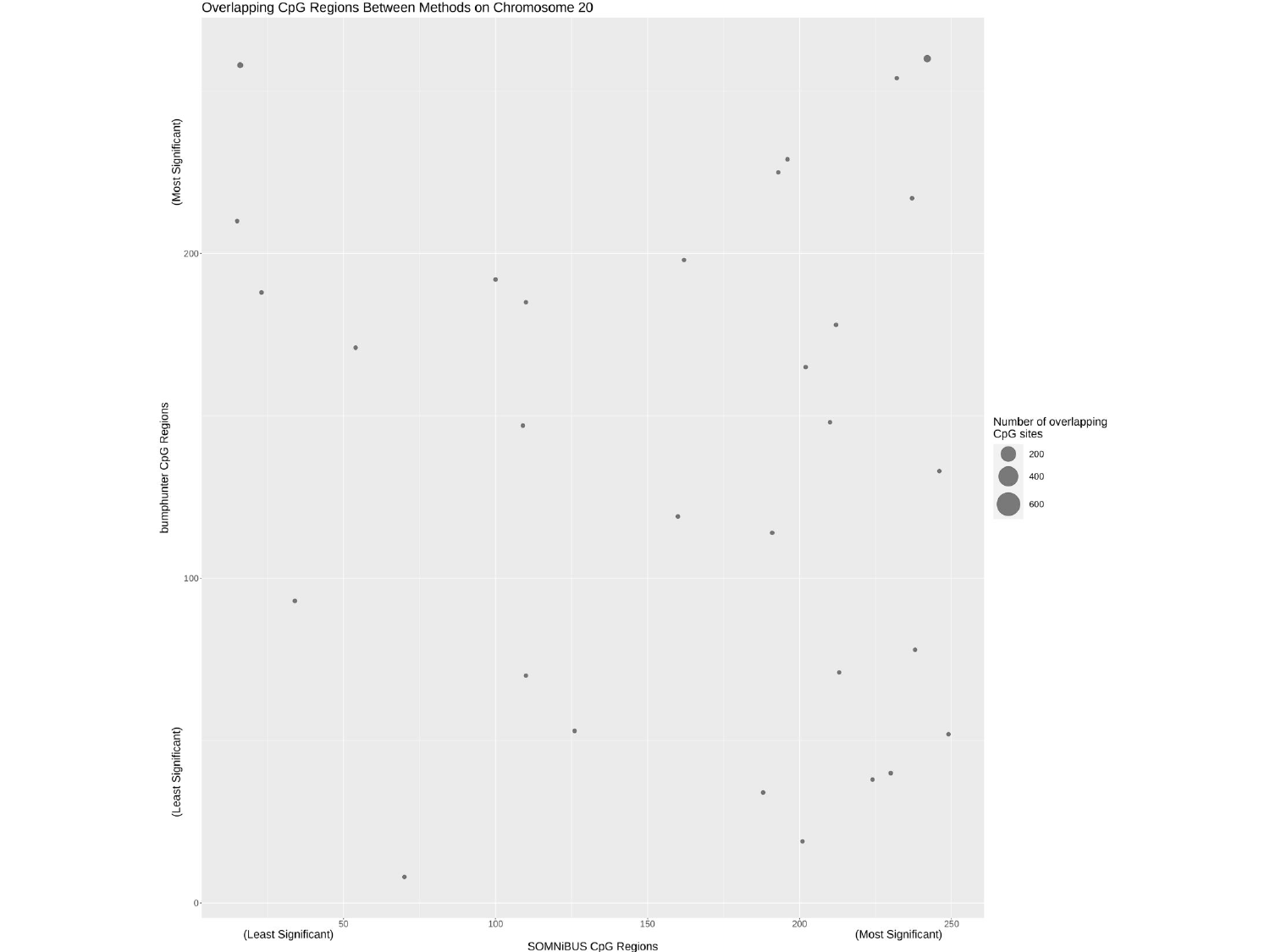

## Slide 22
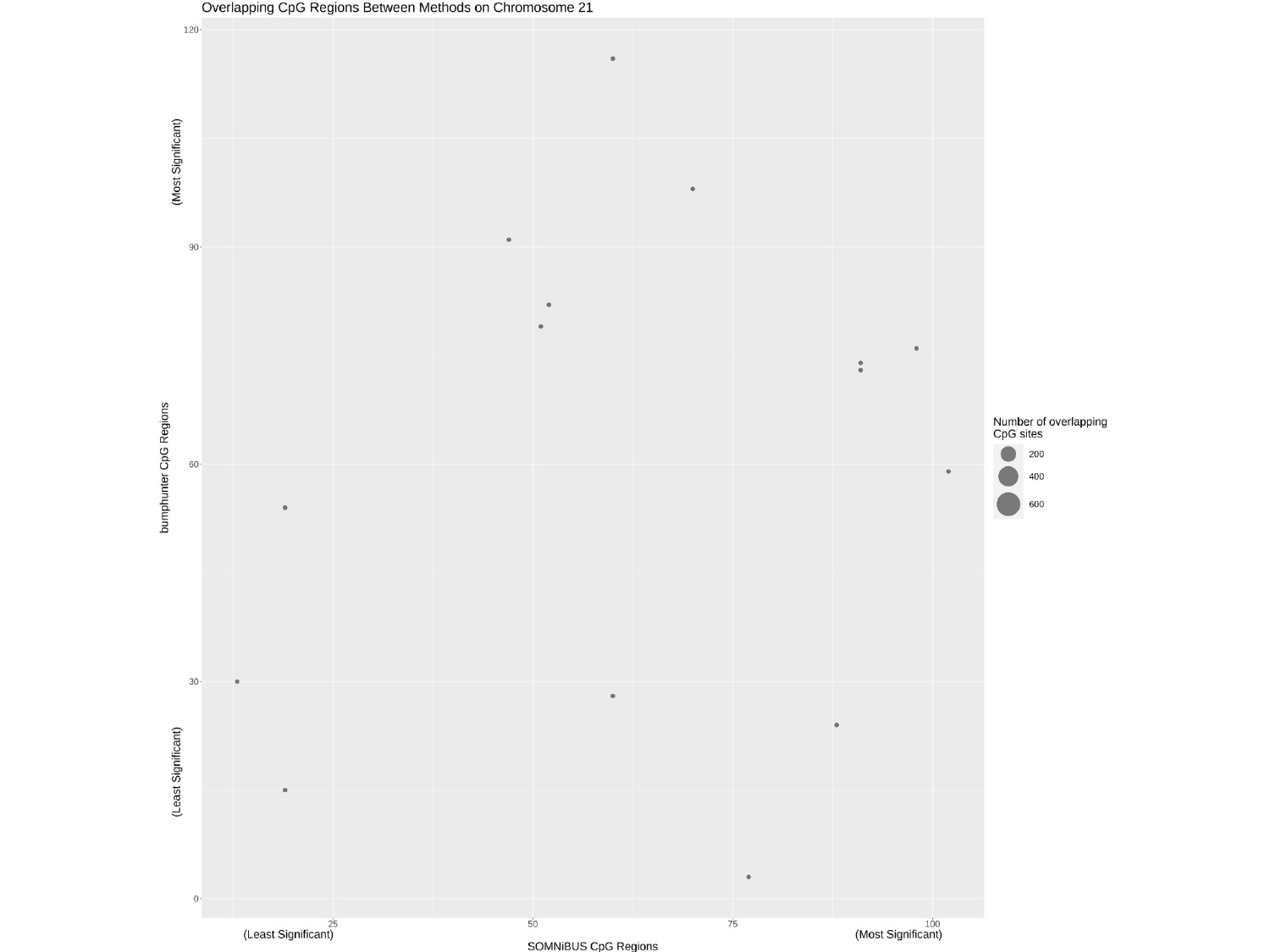

## Slide 23
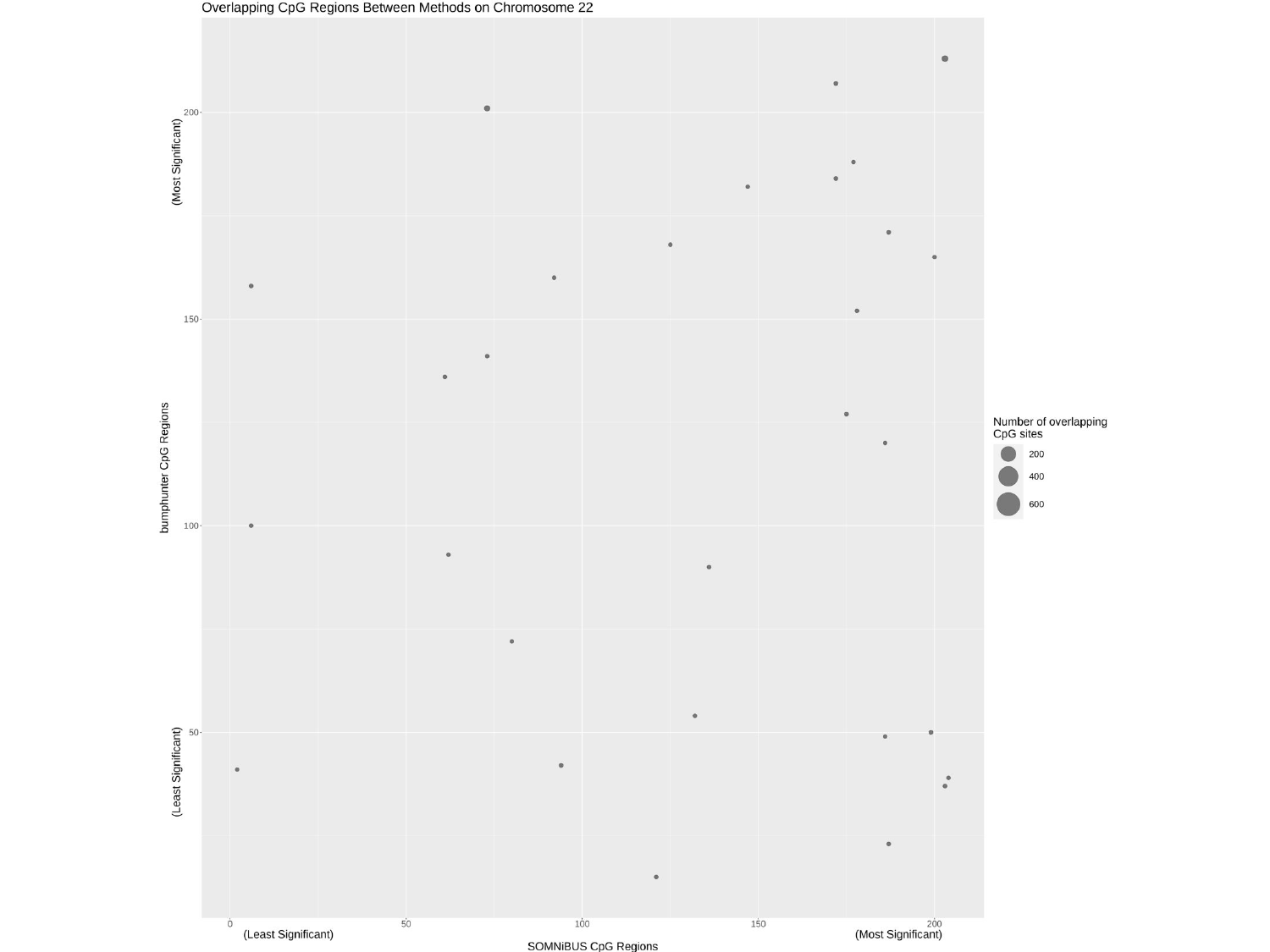

## Slide 24
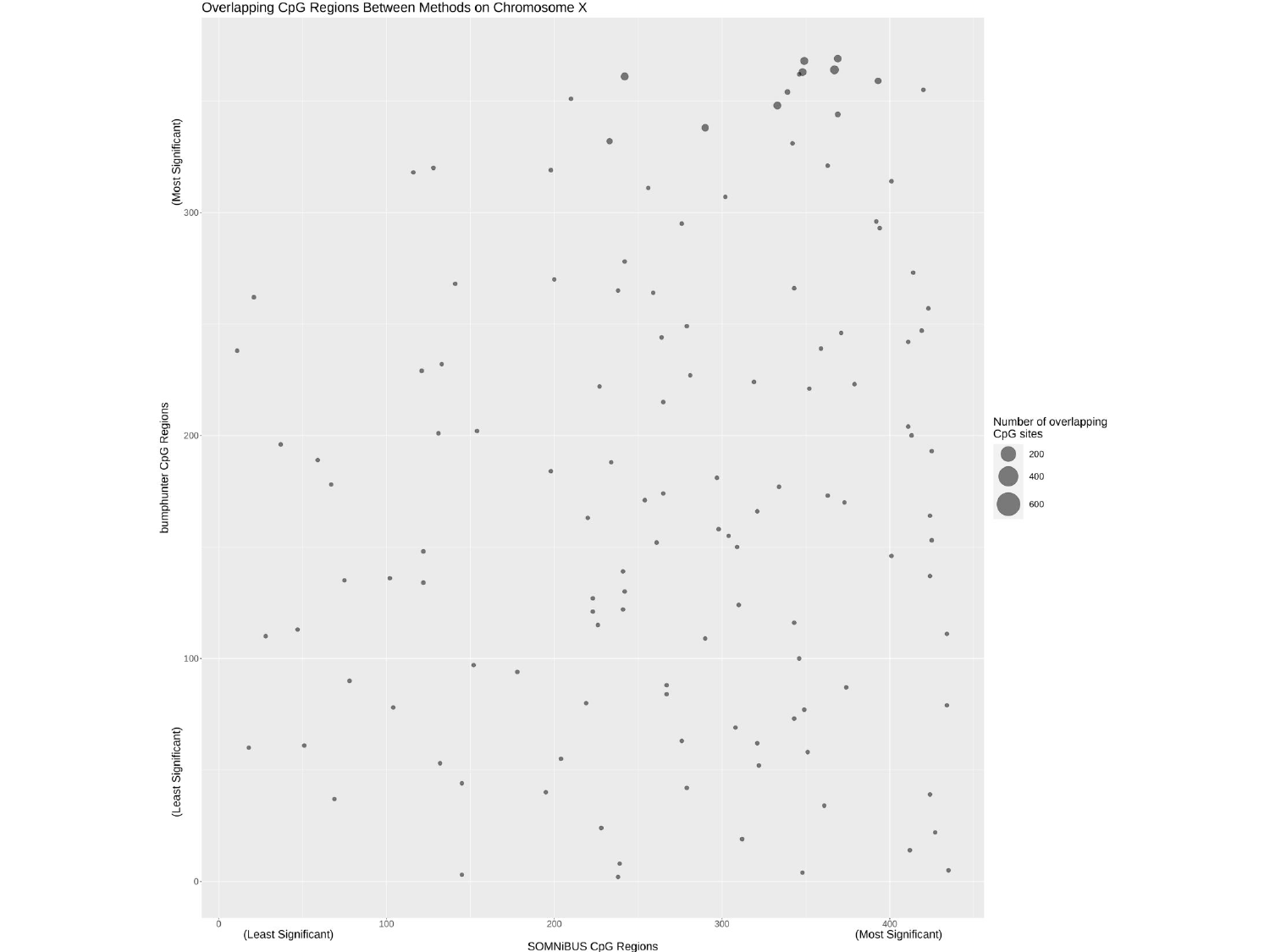

Supplement: Supplementary file 4 — Additional file 4: Overlapping CpG regions partitioned by SOMNiBUS and bumphunter ranked by significance, stratified by chromosome [file 13148_2023_1513_MOESM4_ESM.pptx]
