# Supplementary figures and images for: Novel insights into systemic sclerosis using a sensitive computational method to analyze whole-genome bisulfite sequencing data
Source: Clin Epigenetics. 2023 Jun 3;15:96. doi: 10.1186/s13148-023-01513-w (PMC10239181; doi:10.1186/s13148-023-01513-w)

## Slide 1
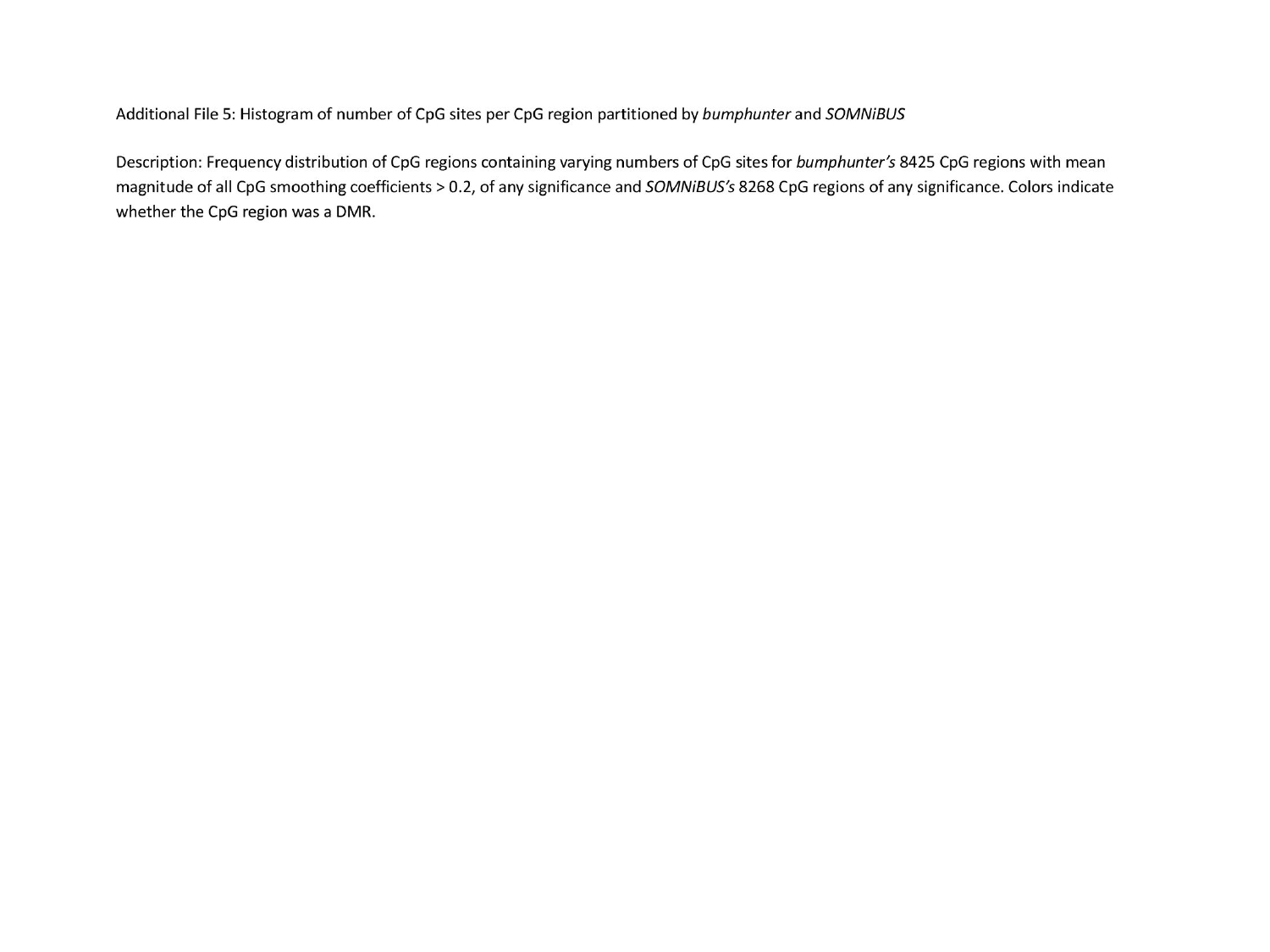

## Slide 2
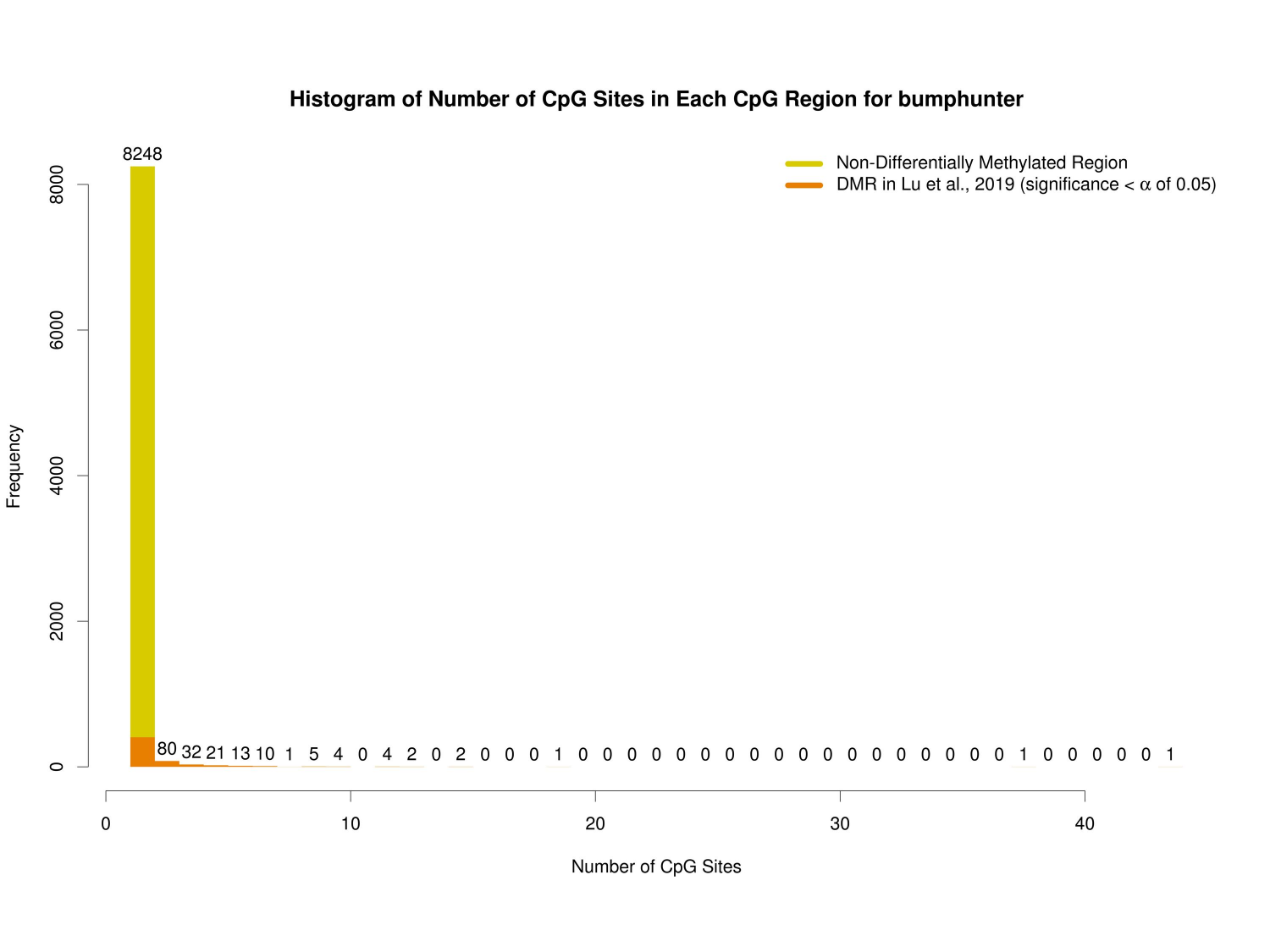

## Slide 3
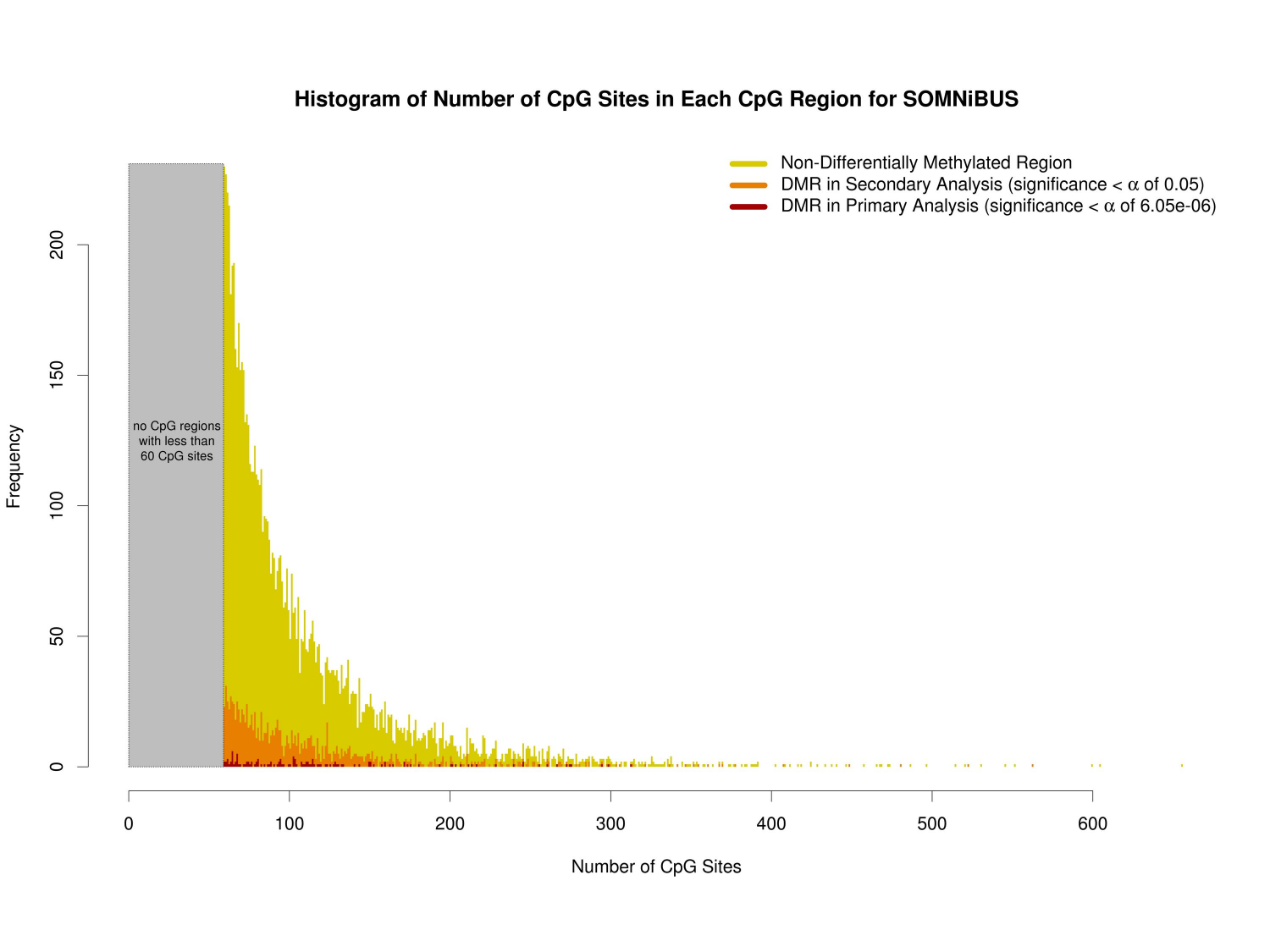

Supplement: Supplementary file 5 — Additional file 5: Histogram of number of CpG sites per CpG region partitioned by bumphunter and SOMNiBUS [file 13148_2023_1513_MOESM5_ESM.pptx]
